# Model for Predicting In-Hospital Mortality of Physical Trauma Patients Using Artificial Intelligence Techniques: Nationwide Population-Based Study in Korea

**DOI:** 10.2196/43757

**Published:** 2022-12-13

**Authors:** Seungseok Lee, Wu Seong Kang, Sanghyun Seo, Do Wan Kim, Hoon Ko, Joongsuck Kim, Seonghwa Lee, Jinseok Lee

**Affiliations:** 1 Department of Biomedical Engineering Kyung Hee University Yong-in Republic of Korea; 2 Department of Trauma Surgery Jeju Regional Trauma Center Cheju Halla General Hospital Jeju Republic of Korea; 3 Department of Radiology Wonkwang University Hospital Iksan Republic of Korea; 4 Department of Thoracic and Cardiovascular Surgery Chonnam National University Hospital Chonnam National University Medical School Gwangju Republic of Korea; 5 Department of Emergency Medicine Jeju Regional Trauma Center Cheju Halla General Hospital Jeju Republic of Korea

**Keywords:** artificial intelligence, trauma, mortality prediction, international classification of disease, injury, prediction model, severity score, emergency department, Information system, deep neural network

## Abstract

**Background:**

Physical trauma–related mortality places a heavy burden on society. Estimating the mortality risk in physical trauma patients is crucial to enhance treatment efficiency and reduce this burden. The most popular and accurate model is the Injury Severity Score (ISS), which is based on the Abbreviated Injury Scale (AIS), an anatomical injury severity scoring system. However, the AIS requires specialists to code the injury scale by reviewing a patient's medical record; therefore, applying the model to every hospital is impossible.

**Objective:**

We aimed to develop an artificial intelligence (AI) model to predict in-hospital mortality in physical trauma patients using the International Classification of Disease 10th Revision (ICD-10), triage scale, procedure codes, and other clinical features.

**Methods:**

We used the Korean National Emergency Department Information System (NEDIS) data set (N=778,111) compiled from over 400 hospitals between 2016 and 2019. To predict in-hospital mortality, we used the following as input features: ICD-10, patient age, gender, intentionality, injury mechanism, and emergent symptom, Alert/Verbal/Painful/Unresponsive (AVPU) scale, Korean Triage and Acuity Scale (KTAS), and procedure codes. We proposed the ensemble of deep neural networks (EDNN) via 5-fold cross-validation and compared them with other state-of-the-art machine learning models, including traditional prediction models. We further investigated the effect of the features.

**Results:**

Our proposed EDNN with all features provided the highest area under the receiver operating characteristic (AUROC) curve of 0.9507, outperforming other state-of-the-art models, including the following traditional prediction models: Adaptive Boosting (AdaBoost; AUROC of 0.9433), Extreme Gradient Boosting (XGBoost; AUROC of 0.9331), ICD-based ISS (AUROC of 0.8699 for an inclusive model and AUROC of 0.8224 for an exclusive model), and KTAS (AUROC of 0.1841). In addition, using all features yielded a higher AUROC than any other partial features, namely, EDNN with the features of ICD-10 only (AUROC of 0.8964) and EDNN with the features excluding ICD-10 (AUROC of 0.9383).

**Conclusions:**

Our proposed EDNN with all features outperforms other state-of-the-art models, including the traditional diagnostic code-based prediction model and triage scale.

## Introduction

Physical trauma–related mortality places a heavy burden on individuals and society. Accurately estimating mortality risk enhances treatment efficiency and reduces this burden. To date, there are various models to predict the severity of physical trauma patients [1-7]. Among them, the most popular and accurate model is the Injury Severity Score (ISS) developed in the 1970s and based on the Abbreviated Injury Scale (AIS), an anatomical injury severity scoring system [1,8]. However, the AIS requires specialists to code the injury scale by reviewing a patient's medical record; therefore, applying the model to every hospital is impossible. To overcome these shortcomings, the following International Classification of Diseases (ICD)–based severity models have been introduced: ICD-based Injury Severity Score (ICISS)[9], trauma mortality models using International Classification of Disease 10th Revision (ICD-10) (TMPM-ICD10) [10], and Mortality Ratio-adjusted Injury Severity Score (EMR-ISS) [11]. However, ICD-based models are not as accurate as AIS-based models [8]. Since 2016, all emergency medical institutions in Korea have introduced the Korean Triage and Acuity Scale (KTAS), an emergency department (ED) triage system composed of 5 levels [12]. However, the KTAS relies on the practitioner’s judgment and may introduce bias and be prone to human error [13].

Artificial intelligence (AI) is widely used to find complex associations between various features in medical applications [14-16], such as individual injuries and mortality. We recently proposed AI technology utilizing AIS codes that outperformed conventional ISS [1], providing a favorable area under the receiver operating characteristic (AUROC) of 0.908 [17]. Tran et al [18] also used AI technology for mortality prediction using the ICD-10 from the National Trauma Database (NTDB) data set, but the AUROC value was not as high as that of our previous proposed AI model.

We aimed to construct an AI model to predict in-hospital mortality in physical trauma patients using the National Emergency Department Information System (NEDIS) data set. We hypothesized that an AI model based on ICD-10 with other clinical features is a useful alternative. We compared the predictive performance of our model with other ICD-10-based models, such as the ICISS [9], EMR-ISS [11], and the AI-driven ICD-10-only based model. Finally, we deployed our AI-driven public website to predict in-hospital mortality in physical trauma patients to benefit end users.

## Methods

### Ethics Approval

This study was conducted according to the TRIPOD (Transparent Reporting of a Multivariable Model for Individual Prognosis or Diagnosis) statement [19]. NEDIS data were provided by the National Emergency Medical Center (data acquisition number N20212920825).

### Patients and Data Set for AI Model

The NEDIS data set was collected mandatorily from 2016 to 2019 from over 400 hospitals in South Korea. The inclusion criteria were as follows: (1) physical trauma patients (but not psychological) with a diagnostic code of S or T based on the Korean version of the ICD-10; (2) patients admitted to the intensive care unit (ICU) or general ward from the ED; and (3) patients admitted to the ICU or general ward after surgery or a procedure from the ED. The exclusion criteria were as follows: (1) patients without diagnostic codes starting with S or T (eg, S001, T063; all physical traumatic patients include S or T code. The S code represents the trauma in a single body region, and the T code represents the trauma in multiple or unspecified regions); (2) patients with diagnostic code of frostbite (T33-T35.6), intoxication (T36-T65), and unspecified injury or complication (T66-T78, T80-T88); (3) patients transferred to another hospital or discharged from the ED after treatment; (4) patients transferred to another hospital or discharged without notification to staffs at hospitals; (5) patients who died in the ED before ICU or general ward admission; and (6) missing information.

**Figure 1:**
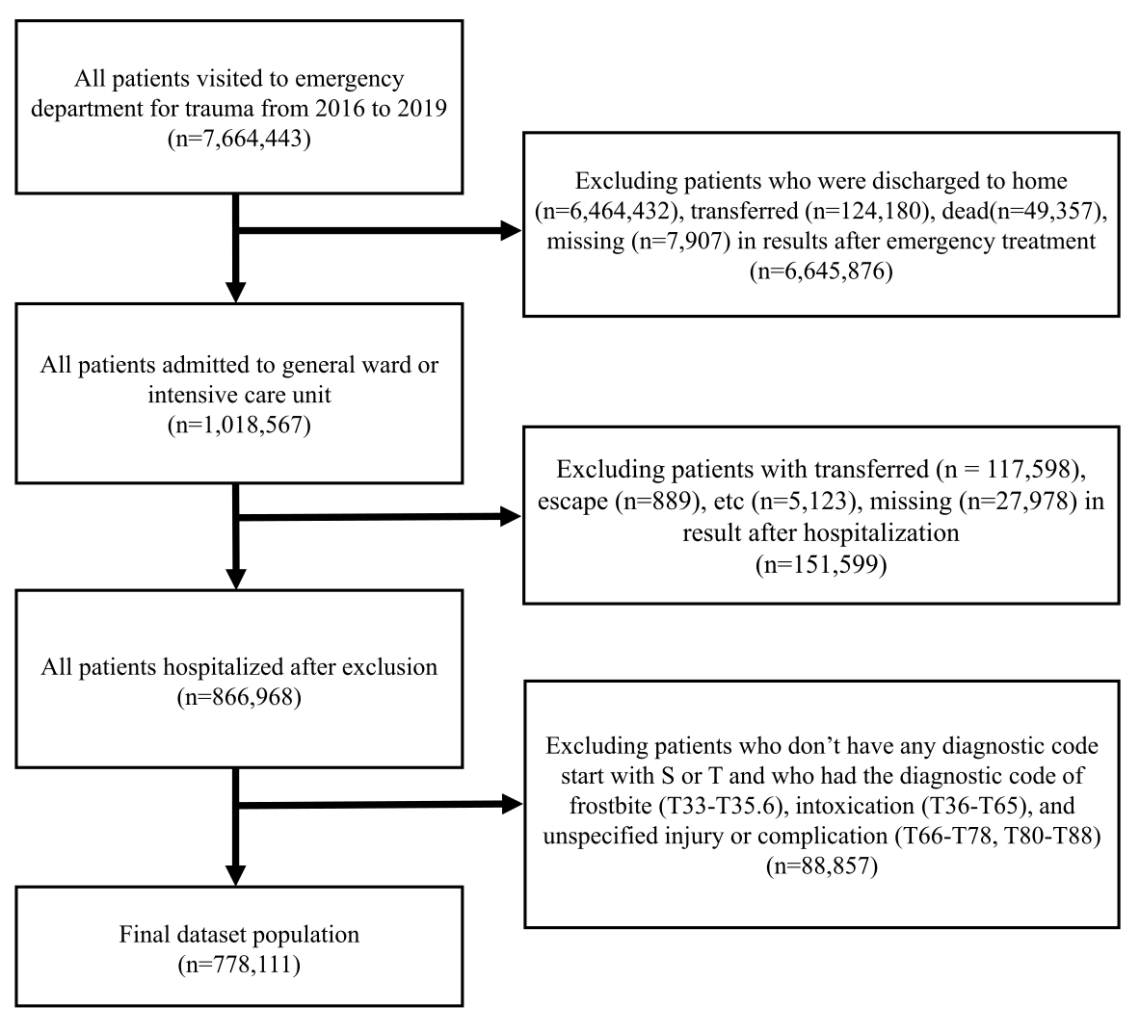
Flowchart of the patient selection process.

More specifically, we first collected 7,664,443 patients with a nondisease identifier comprising trauma patients. Since our primary outcome was to predict in-hospital mortality in trauma patients, we had to exclude unrelated patients. We then excluded all nonhospitalized patient information (n=6,464,432, 84.34%). The second most commonly excluded data were from patients transferred to another hospital (n=241,778, 3.15%). For transferred patients, the NEDIS policy of deidentification is to assign a new anonymous ID number; thus, the data is redundant. In addition, we excluded deceased ED patients (n=49,357, 0.64%) due to insufficient information about diagnostic codes, procedure codes, and other clinical features. Moreover, we excluded escaped patients during hospitalization (n=889, 0.01%) and patients with missing data (n=35,885, 4.68%), not including mortality information. A final total of 778,111 patient data were used for training and testing our AI model (Figure 1).

We used the following variables in NEDIS data: age, gender, intentionality, injury mechanism, emergent symptom, Alert/Verbal/Painful/Unresponsive (AVPU) scale, initial KTAS, altered KTAS, ICD-10 codes, procedure codes of surgical operation or interventional radiology, and in-hospital mortality. All included variables for the AI model are summarized in Table 1. A total of 938 AI model input features (categories) were considered from 10 variables. The AVPU scale is a simplified version of the Glasgow Coma Scale (GCS) [20,21] and includes 4 categories: A, alert; V, verbal responsive (drowsy); P, painful response (stupor, semicoma); and U, unresponsive (coma). KTAS was developed as a severity triage in the ED in 2012, based on the Canadian Triage and Acuity Scale (CTAS) [12]. KTAS is a standardized triage tool to avoid complexity and ambiguity and includes 5 categories: level 1, resuscitation; level 2, emergent; level 3, urgent; level 4, less urgent; level 5, nonurgent. According to NEDIS policy, KTAS should be conducted by a certified faculty, and the initial KTAS should be assessed within 2 minutes of ED admission. The altered KTAS should be assessed when the ED patient deteriorates before moving to the operating room, ICU, or general ward. Regarding ICD-10, we considered 856 codes starting with S or T. The procedure codes, which are used to claim from the National Health Insurance Review and Assessment Service, include surgery and angioembolization and are more specifically categorized as follows and summarized in Table S1 in Multimedia Appendix 1: (1) head procedure; (2) torso procedure-vascular; (3) torso procedure-abdomen; (4) torso procedure-chest; (5) torso procedure-heart; and (6) extracorporeal membrane oxygenation (ECMO). The primary outcome was in-hospital mortality, defined as a patient with a dead result code and discharged with medical futility in NEDIS.

**Table 1:** Included variables of the Korean National Emergency Department Information System (NEDIS) for the artificial intelligence (AI) model.

Value, n	Variables	Type	Description
1	Age	26 categories	5-year-old unit, classification
2	Gender	2 categories	M: male F: female
3	Intentionality	5 categories	1: accidental, unintentional 2: self-harm, suicide 3: violence, assault 4: other specified 5: unspecified 6: no data
4	Injury mechanism	16 categories	1: traffic accident-car 2: traffic accident-bike 3: traffic accident-motorcycle 4: traffic accident-etc 5: traffic accident-unspecified 6: fall 7: slip down 8: struck 9: firearm/cut/pierce 10: machine 11: fire, flames or heat 12: drowning or nearly 13: poisoning 14: choking, hanging 15: etc 16: unknown 17: no data
5	Emergent symptoms	2 categories	Y: emergency N: nonemergency
6	AVPU^a^ scale	4 categories	A: alert V: verbal response (drowsy) P: painful response (semicoma) U: unresponsive (coma) N: unknown
7	Initial KTAS^b^	7 categories	1: Level 1 (resuscitation) 2: Level 2 (emergency) 3: Level 3 (urgency) 4: Level 4 (less urgency) 5: Level 5 (nonurgency) 6: etc 7: unknown 8: no data
8	Altered KTAS	5 categories	1: Level 1 (resuscitation) 2: Level 2 (emergency) 3: Level 3 (urgency) 4: Level 4 (less urgency) 5: Level 5 (nonurgency) 6: etc 7: no data
9	Diagnostic code at discharge	865 categories	ICD-10^c^ codes starting S^d^ or T^e^
10	Procedure code after hospitalized	6 categories	Procedure code including surgery or interventional radiology

^a^AVPU: Alert/Verbal/Painful/Unresponsive.

^b^KTAS: Korean Triage and Acuity Scale.

^c^ICD-10: International Classification of Disease 10th Revision.

^d^Represents trauma in a single body region.

^e^Represents trauma in multiple or unspecified regions.

### Data Split, Data Balancing, and Cross-Validation

The data set in this study comprised both training and testing data (Table S2 in Multimedia Appendix 1). Data from 778,111 patients were divided into training and testing data with a ratio of 8:2 in a stratified fashion. The testing set was used only to independently test our developed AI model and not for training or internal validation.

We first performed 5-fold cross-validation using the training data to confirm its generalization ability. The training data set (n=622,488, 80%) was randomly shuffled and stratified into 5 equal groups, of which 4 groups were selected from training the model, and the remaining group was used for internal validation. This process was repeated 5 times by shifting the internal validation group. Our finalized AI model is described in the subsequent sections and was used to evaluate performance using the isolated testing data.

Since the number of survived patients (n=611,481, 98.23%) was much higher than that of deceased patients (n=11,007, 1.77%), we upsampled the survived patient data using the Synthetic Minority Oversampling Technique (SMOTE) during the model update [22]. By balancing the 2 groups, we prevented bias toward the survived patient data.

### Feature Analysis

To analyze the effects on mortality prediction from 914 features, we applied 3 machine-learning algorithms: Adaptive Boosting (AdaBoost) [23], Extreme Gradient Boosting (XGBoost) [24], and light gradient boosting machine (LightGBM) [25]. We also considered 4 ensemble models: AdaBoost with XGBoost, AdaBoost with LightGBM, XGBoost with LightGBM, and a combination of the 3 models. Finally, among 7 machine learning models, we chose the best prediction model and presented its feature importance analysis, listing features in the order that they contributed to the mortality prediction.

Performance evaluations were based on 5-fold cross-validation using 5 metrics: sensitivity, specificity, accuracy, balanced accuracy, and AUROC.

### AI Prediction Model Development and Statistical Analysis

**Figure 2:**
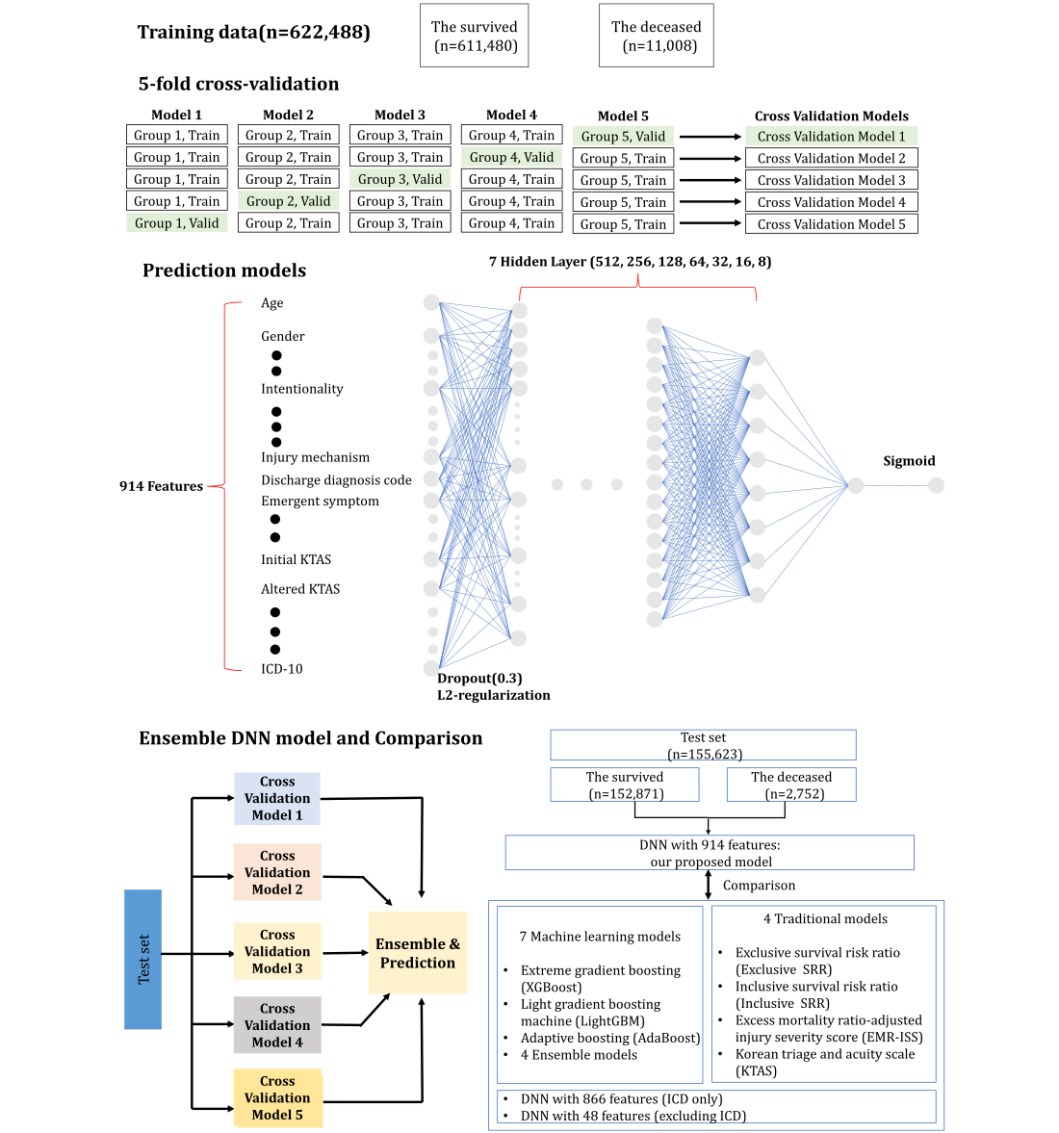
Process flow of our artificial intelligence (AI) model development: data, deep neural network (DNN) architecture, ensemble DNN model, and performance comparison. AdaBoost: Adaptive Boosting; EMR-ISS: mortality ratio-adjusted Injury Severity Score; ICD: International Classification of Diseases; KTAS: Korean Triage and Acuity Scale; LightGBM: light gradient boosting machine; SRR: survival risk ratio; XGBoost: Extreme Gradient Boosting.

We developed a deep neural network (DNN)–based AI model using 914 features, including ICD-10 as an input layer. To find the best model, we searched hyperparameters, such as layer depth and width for fully connected (FC) layers. The last FC output layer was fed into a sigmoid layer, which provided the mortality probability. After the hyperparameter search, we found the best model with a 9-layer DNN, which comprised an input layer, 7 FC layers as hidden layers, and an output layer. The input layer was fed into a series of 7 FC layers, consisting of 512, 256, 128, 64, 32, 16, and 8 nodes, respectively. We applied dropout with a rate of 0.3 and L2 regularization for the FC hidden layers. Figure 2 shows the process flow of the AI development and DNN architecture. The prediction performance of our proposed 9-layer DNN model was evaluated with 5-fold cross-validation. Subsequently, for the final DNN-based AI model, we adopted an ensemble approach to combine the 5 models from the 5-fold cross-validation. The 914 features were inputs to 5 cross-validation models, and each provided mortality probabilities. A total of 5 probabilities were averaged, known as soft voting. Based on the ensemble DNN model, the prediction performance was evaluated with the isolated testing data set (n=155,623, 20%).

We trained the models with an Adam optimizer and binary cross-entropy cost function with a learning rate of 0.001 and a batch size of 32. We implemented the models using Python (version 3.7.13) with TensorFlow (version 2.8.0), Keras (version 2.8.0), NumPy (version 1.21.6), Pandas (version 1.3.5), Matplotlib (version 3.5.1), and Scikit-learn (version 1.0.2). All statistical analyses were performed using R software version 4.1.2 (R Foundation for Statistical Computing). As appropriate, proportions were compared using the chi-square test or Fisher exact test. A *P* value <.05 was considered statistically significant.

### Conventional Metrics Based on Diagnostic Code

We applied conventional metrics based on ICD-10. ICISS utilizes survival risk ratios (SRRs) to calculate the probability of survival [9]. SRR is defined as the number of survived patients with a specific injury code divided by the number of all patients with the specific same injury code. A patient's probability of survival (Ps) is determined by multiplying all SRRs of the injury codes from the patient [9]. The traditional ICISS was calculated as the product of Ps for as many as 10 injuries [26]. Two different methods were performed to calculate ICISS. First, the inclusive SRR was calculated for each injury irrespective of the associated injury [9]. Second, the exclusive SRR was calculated by the number of survivors who had an isolated specific injury divided by the total number of patients who only had that injury [9]. Thus, patients with multiple injuries were excluded from the calculation of exclusive SRR [9]. Regarding EMR-ISS, an injury severity grade similar to AIS was produced from ICD-10 codes based on the quintile of the EMR for each ICD-10 code [11]. The EMR-ISS was calculated from 3 maximum severity grades using data from the National Health Insurance data set, the Industrial Accident Compensation Insurance data set, and the National Death Certificate database from 2001 to 2003 [11].

## Results

### Initial Findings

Of the 778,111 patients included in the final analysis, 13,760 (1.77%) died during hospitalization (13,667 had a deceased code, and 93 were discharged with a medical futility code). Table 2 shows a comparison of included variables between deceased and surviving patients, and Table S3 in Multimedia Appendix 1 shows the ICD-10 comparison between deceased and surviving patients.

**Table 2:** Comparison of included variables of the Korean National Emergency Department Information System (NEDIS) between deceased and survived patients.

Variables		Deceased (N=13,760), n (%)	Survived (N=764,351), n (%)	*P* value	
**Age (years)**					<.001
	<1	16 (0.1)	2139 (0.3)		
	1-4	63 (0.5)	9706 (1.3)		
	5-9	38 (0.3)	16,345 (2.1)		
	10-14	36 (0.3)	16,788 (2.2)		
	15-19	171 (1.2)	25,776 (3.4)		
	20-24	229 (1.7)	31,000 (4.1)		
	25-29	214 (1.6)	33,241 (4.3)		
	30-34	176 (1.3)	32,151 (4.2)		
	35-39	276 (2)	38,611 (5.1)		
	40-44	326 (2.4)	42,013 (5.5)		
	45-49	583 (4.2)	55,126 (7.2)		
	50-54	768 (5.6)	66,276 (8.7)		
	55-59	1055 (7.7)	78,447 (10.3)		
	60-64	1110 (8.1)	66,899 (8.8)		
	65-69	1155 (8.4)	52,900 (6.9)		
	70-74	1388 (10.1)	50,396 (6.6)		
	75-79	2028 (14.7)	58,334 (7.6)		
	80-84	1925 (14)	48,440 (6.3)		
	85-89	1320 (9.6)	27,670 (3.6)		
	90-94	665 (4.8)	9723 (1.3)		
	95-99	189 (1.4)	21,08 (0.3)		
	100-104	25 (0.2)	226 (0)		
	105-109	4 (0)	25 (0)		
	110-114	0 (0)	9 (0)		
	115-119	0 (0)	2 (0)		
**Procedure code**					
	Head procedure	2473 (18)	6419 (0.8)	<.001	
	Torso procedure-vascular	880 (6.4)	4961 (0.6)	<.001	
	Torso procedure-abdomen	810 (5.9)	4544 (0.6)	<.001	
	Torso procedure-chest	1209 (8.8)	7228 (0.9)	<.001	
	Torso procedure-heart	39 (0.3)	127 (0)	<.001	
	ECMO^a^	183 (1.3)	39 (0)	<.001	
**Initial KTAS^b^**					
	Level 1	3800 (27.6)	2812 (0.4)	<.001	
	Level 2	4209 (30.6)	49,234 (6.4)	<.001	
	Level 3	3306 (24)	270,574 (35.4)	<.001	
	Level 4	2020 (14.7)	346,663 (45.4)	<.001	
	Level 5	235 (1.7)	49,892 (6.5)	<.001	
	Not classified	4 (0)	301 (0)	.698	
	Unspecified	0 (0)	13 (0)	>.99	
	Missing data	186 (1.4)	44,862 (5.9)	<.001	
**Altered KTAS**					
	Level 1	2938 (21.4)	1921 (0.3)	<.001	
	Level 2	3173 (23.1)	35,356 (4.6)	<.001	
	Level 3	2784 (20.2)	241,201 (31.6)	<.001	
	Level 4	873 (6.3)	189,314 (24.8)	<.001	
	Level 5	108 (0.8)	27,355 (3.6)	<.001	
	Not classified	0 (0)	5 (0)	>.99	
	Missing data	3884 (28.2)	269,199 (35.2)	<.001	
**Intentionality**					
	Accidental, unintentional	12078 (87.8)	574,556 (75.2)	<.001	
	Suicide, intentional self-harm	248 (1.8)	6235 (0.8)	<.001	
	Assault, violence	113 (0.8)	12,989 (1.7)	<.001	
	Other specified	132 (1)	1694 (0.2)	<.001	
	Unspecified	548 (4)	12,225 (1.6)	<.001	
	Missing data	641 (4.7)	156,652 (20.5)	<.001	
**Injury mechanism**					
	Traffic accident-car	1154 (8.4)	98,320 (12.9)	<.001	
	Traffic accident-bike	450 (3.3)	20,692 (2.7)	<.001	
	Traffic accident-motorcycle	1020 (7.4)	31,957 (4.2)	<.001	
	Traffic accident-pedestrian, train, airplane, ship, etc	1925 (14)	35,898 (4.7)	<.001	
	Traffic accident-unknown	18 (0.1)	197 (0)	<.001	
	Fall down	2374 (17.3)	76,714 (10)	<.001	
	Slip down	3859 (28)	16,8677 (22.1)	<.001	
	Struck by person or object	713 (5.2)	60,518 (7.9)	<.001	
	Firearm/cut (sharp or object)/piece	159 (1.2)	39,515 (5.2)	<.001	
	Machine	54 (0.4)	16,991 (2.2)	<.001	
	Fire, flames, or heat	207 (1.5)	6587 (0.9)	<.001	
	Drowning or nearly drowning	20 (0.1)	203 (0)	<.001	
	Poisoning	62 (0.5)	1811 (0.2)	<.001	
	Choking, hanging	146 (1.1)	436 (0.1)	<.001	
	Others-rape, electric	323 (2.3)	35,461 (4.6)	<.001	
	Unknown	635 (4.6)	13,722 (1.8)	<.001	
	Missing data	641 (4.7)	156,652 (20.5)	<.001	
**Emergent symptom**					
	Yes	13351 (97)	69,7118 (91.2)	<.001	
	No	409 (3)	67,228 (8.8)	<.001	
	Unspecified	0 (0)	5 (0)	>.99	
**AVPU^c^ scale**					
	Alert	5403 (39.3)	579,669 (75.8)	<.001	
	Verbal response (drowsy)	1393 (10.1)	12,085 (1.6)	<.001	
	Painful response (stupor, semicoma)	3218 (23.4)	5581 (0.7)	<.001	
	Unresponsive (coma)	3049 (22.2)	847 (0.1)	<.001	
	Unspecified response	697 (5.1)	166,169 (21.7)	<.001	
**Sex**					
	Male	9050 (65.8)	434,280 (56.8)	<.001	

^a^ECMO: extracorporeal membrane oxygenation.

^b^KTAS: Korean Triage and Acuity Scale.

^c^AVPU: Alert/Verbal/Painful/Unresponsive.

### K-Fold Cross-Validation Results

Table 3 summarizes the 5-fold cross-validation results. Our model used all 914 features, including ICD-10, and provided the highest balanced accuracy (0.8718) and AUROC (0.9513) values. Among the machine learning models, AdaBoost provided the highest balanced accuracy (0.8603) and AUROC (0.9442). Any ensemble models from the combination of AdaBoost, XGBoost, and LightGBM did not improve accuracy above our model or AdaBoost. Compared to our model, traditional methods produced lower balanced accuracy and AUROC values. More specifically, inclusive SRR resulted in a lower balanced accuracy of 0.7888 and AUROC of 0.8266, while exclusive SRR resulted in 0.7931 and 0.8737, and EMR-ISS yielded 0.7571 and 0.6108, respectively. KTAS resulted in an even lower balanced accuracy of 0.5372 and AUROC of 0.1057.

Of the models considering 866 features of ICD-10 only, DNN demonstrated the highest balanced accuracy (0.8234) and AUROC (0.8975), followed by AdaBoost, the ensemble of AdaBoost and XGBoost, and the ensemble of AdaBoost and LightGBM. However, the models generated much lower balanced accuracy and AUROC values compared to models considering 48 features, excluding ICD-10.

**Table 3:** Results of the 5-fold cross-validation.

Model		Sensitivity, mean (SD)	Specificity, mean (SD)	Accuracy, mean (SD)	Balanced accuracy, mean (SD)	AUROC^a^, mean (SD)
**Using all 914 features (including ICD-10^b^)**						
	Proposed model (DNN^c^)	0.8599 (0.0151)	0.8838 (0.0097)	0.8834 (0.0093)	0.8718 (0.0036)	0.9513 (0.0023)
	AdaBoost^d^	0.818 (0.0100)	0.9025 (0.0006)	0.9010 (0.0005)	0.8603 (0.0048)	0.9442 (0.0020)
	XGBoost^e^	0.8105 (0.0085)	0.8865 (0.0011)	0.8854 (0.0010)	0.8485 (0.0037)	0.9354 (0.0018)
	LightGBM^f^	0.8112 (0.0080)	0.8861 (0.0018)	0.8848 (0.0016)	0.8486 (0.0032)	0.9354 (0.0019)
	AdaBoost+XGBoost	0.8109 (0.0073)	0.8882 (0.0013)	0.8868 (0.0012)	0.8496 (0.0034)	0.9367 (0.0017)
	AdaBoost+LightGBM	0.8118 (0.0081)	0.8875 (0.0014)	0.8862 (0.00130)	0.8497 (0.0035)	0.9367 (0.0018)
	XGBoost+LigtGBM	0.8104 (0.0079)	0.8865 (0.0010)	0.8851 (0.0009)	0.8484 (0.0035)	0.9354 (0.0018)
	AdaBoost+XGBoost+LightGBM	0.8107 (0.0075)	0.8871 (0.0011)	0.8857 (0.0010)	0.8489 (0.0033)	0.9361 (0.0018)
**Using 866 features (ICD-10 only)**						
	DNN	0.8294 (0.0153)	0.8175 (0.009)	0.8177 (0.0086)	0.8234 (0.0037)	0.8975 (0.0023)
	AdaBoost	0.7586 (0.0157)	0.8493 (0.0048)	0.8477 (0.0045)	0.8039 (0.0057)	0.8796 (0.0030)
	XGBoost	0.6575 (0.0141)	0.8939 (0.0035)	0.8897 (0.0032)	0.7757 (0.0055)	0.8627 (0.0033)
	LightGBM	0.6585 (0.0115)	0.8937 (0.0024)	0.8896 (0.0022)	0.7761 (0.0049)	0.8635 (0.0037)
	AdaBoost+XGBoost	0.6637 (0.0065)	0.8922 (0.0017)	0.8882 (0.0016)	0.7780 (0.0027)	0.8785 (0.0029)
	AdaBoost+LightGBM	0.6640 (0.0076)	0.8918 (0.0012)	0.8878 (0.0011)	0.7779 (0.0032)	0.8786 (0.0031)
	XGBoost+LigtGBM	0.6590 (0.0117)	0.8932 (0.0024)	0.8891 (0.0022)	0.7761 (0.0048)	0.8635 (0.0035)
	AdaBoost+XGBoost+LightGBM	0.6624 (0.0070)	0.8924 (0.0017)	0.8883 (0.0016)	0.7774 (0.0029)	0.8784 (0.0028)
**Using 48 features (excluding ICD-10)**						
	DNN	0.8003 (0.0266)	0.9072 (0.0161)	0.9053 (0.0154)	0.8537 (0.0068)	0.9398 (0.003)
	AdaBoost	0.8148 (0.0125)	0.8922 (0.0022)	0.8908 (0.0022)	0.8535 (0.0062)	0.9380 (0.0025)
	XGBoost	0.8294 (0.0056)	0.863 (0.0033)	0.8623 (0.0032)	0.8462 (0.0018)	0.9328 (0.0022)
	LightGBM	0.8323 (0.0044)	0.8619 (0.0032)	0.8614 (0.0032)	0.8471 (0.0018)	0.9328 (0.0021)
	AdaBoost+XGBoost	0.8303 (0.0058)	0.8635 (0.0029)	0.8630 (0.0028)	0.8469 (0.0019)	0.9337 (0.0022)
	AdaBoost+LightGBM	0.8314 (0.0052)	0.8634 (0.0028)	0.8628 (0.0027)	0.8474 (0.002)	0.9336 (0.0020)
	XGBoost+LigtGBM	0.8321 (0.0046)	0.8618 (0.0032)	0.8613 (0.0031)	0.847 (0.0020)	0.9328 (0.0021)
	AdaBoost+XGBoost+LightGBM	0.8312 (0.0052)	0.8630 (0.0024)	0.8624 (0.0024)	0.8471 (0.0022)	0.9333 (0.0021)
**Traditional methods^g^**						
	Inclusive SRR^h^	0.8953	0.6823	0.7893	0.7888	0.8266
	Exclusive SRR	0.8272	0.7590	0.7936	0.7931	0.8737
	EMR-ISS^i^	0.7867	0.7276	0.7572	0.7571	0.6108
	KTAS^j^	0.9353	0.1390	0.5495	0.5372	0.1057

^a^AUROC: area under the receiver operating characteristic.

^b^ICD-10: International Classification of Disease 10th Revision.

^c^DNN: deep neural network.

^d^AdaBoost: Adaptive Boosting.

^e^XGBoost: Extreme Gradient Boosting.

^f^LightGBM: light gradient boosting machine.

^g^Only yielded a single value, so no SD is reported.

^h^SRR: survival risk ratio.

^i^EMR-ISS: Mortality Ratio-adjusted Injury Severity Score.

^j^KTAS: Korean Triage and Acuity Scale.

### Ranked Feature Importance: Explainable AI

**Figure 3:**
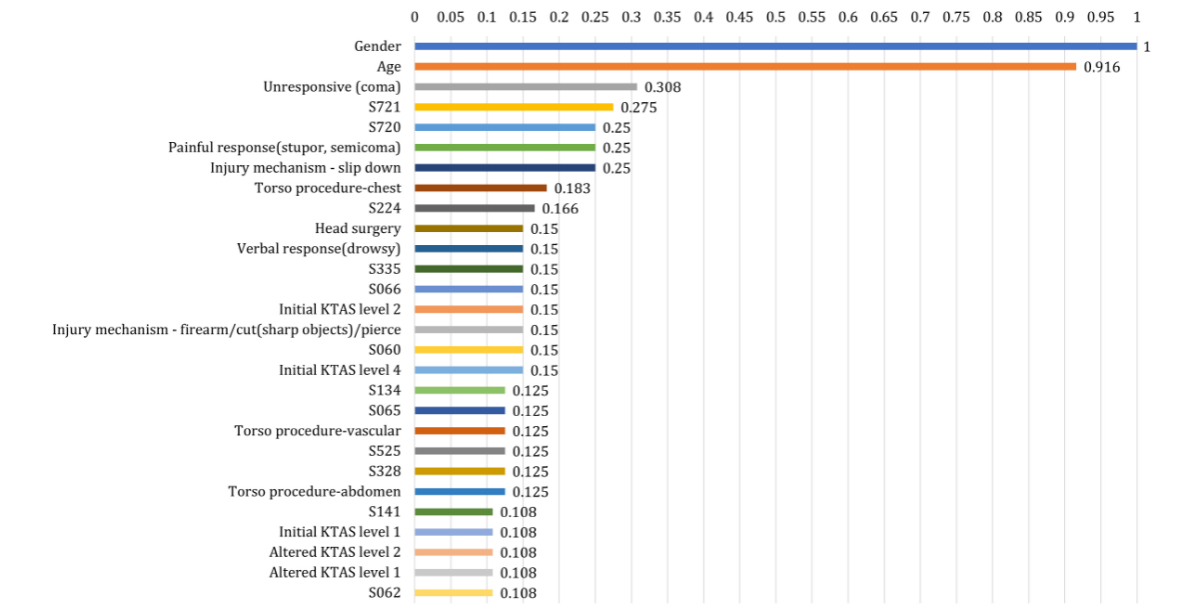
Results of the ranked normalized feature importance from the Adaptive Boosting (AdaBoost) model. KTAS: Korean Triage and Acuity Scale.

To analyze the effects of features, we first applied the data to 3 different machine learning algorithms: AdaBoost, XGBoost, and LightGBM. As summarized in Table 3, the AdaBoost model was the best classifier for predicting mortality in trauma patients. We then performed the feature importance analysis (see Figure 3 for ranked normalized feature importance) to confirm the contribution of each feature.

Based on the AdaBoost, gender had the highest importance value, followed by age, unresponsive (coma), S721 (pertrochanteric fracture of the femur), S720 (fracture of neck of femur), painful response (stupor, semicoma), injury mechanism-slip down, and torso procedure-chest. Among the 914 features, only 71 (7.77%) features had nonzero values indicating that the other 843 features did not contribute to mortality prediction. Table S4 in Multimedia Appendix 1 shows the complete ranked normalized feature importance values. All features with the highest importance value showed a statistically significant difference between the deceased and surviving group (Table 2 and Table S3 in Multimedia Appendix 1).

### Cross-Validation Result of DNNs Using a Different Set of Features According to Importance

We investigated the cross-validation performance from our DNN model with 2 input conditions: (1) the top 71 features having nonzero feature importance values from the AdaBoost, the best among the machine learning models; and (2) all 914 features (Table S5 in Multimedia Appendix 1). The DNN with all 914 features provided a higher balanced accuracy of 0.8718 and AUROC of 0.9513 compared to the DNN with the top 71 features, which had a balanced accuracy of 0.8389 and AUROC of 0.9386. Features with 0 values of feature importance can contribute to mortality prediction. Sensitivity increased by more than 0.1 for the former, whereas specificity decreased by less than 0.05. For the latter, sensitivity increased to 0.8599 from 0.7480, and specificity decreased to 0.8838 from 0.9299. Therefore, we considered all features in our AI model and validated the performance with the isolated testing data.

### Testing Data Results

With the testing data set (n=155,623), our proposed ensemble-based 9-layer DNN showed a sensitivity of 0.8768, specificity of 0.8625, accuracy of 0.8628, balanced accuracy of 0.8697, and AUROC of 0.9507. Furthermore, compared with the cross-validation results, the model was neither overfitted nor underfitted, with minimal differences between cross-validation and testing data results: sensitivity of 0.8599 versus 0.8768, specificity of 0.8838 versus 0.8625, accuracy of 0.8834 versus 0.8628, balanced accuracy 0.8718 versus 0.8697, and AUROC of 0.9513 versus 0.9507.

**Figure 4:**
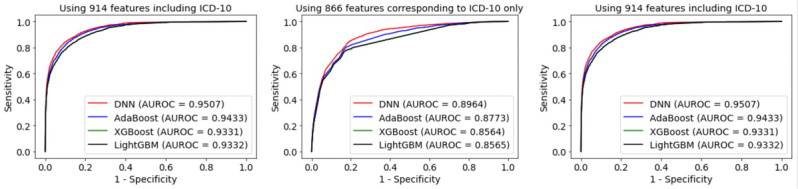
Area under the receiver operating characteristic (AUROC) curves for our model, Adaptive Boosting (AdaBoost), Extreme Gradient Boosting (XGBoost), and light gradient boosting machine (LightGBM): (left) using all 914 features including International Classification of Diseases 10th Revision (ICD-10), (middle) using 48 features excluding ICD-10, and (right) using 866 features with ICD-10 only. DNN: deep neural network.

**Figure 5:**
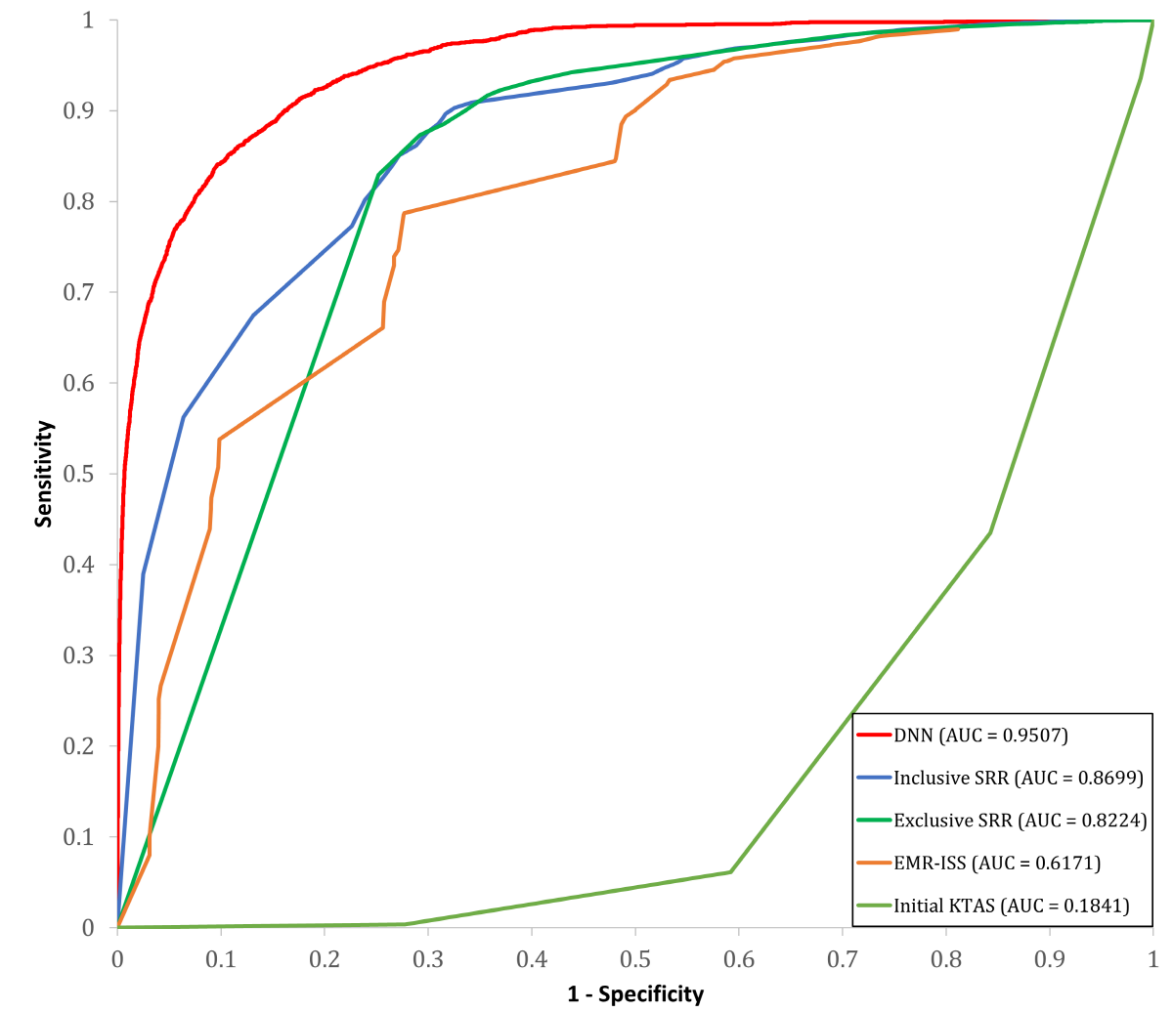
Area under the receiver operating characteristic (AUROC) curves of our model and 4 traditional models. AUC: area under the curve; DNN: deep neural network; EMR-ISS: mortality ratio-adjusted Injury Severity Score; KTAS: Korean Triage and Acuity Scale; SRR: survival risk ratio.

Our proposed ensemble of deep neural networks (EDNN) using all 914 features demonstrated higher values of balanced accuracy and AUROC than any other model (Table 4). Models with 48 features provided the next most accurate prediction results. These results showed the same trend as the cross-validation results. Figure 4 shows the AUROC curves for our model, AdaBoost, XGBoost, and LightGBM, which are plotted according to the following features: all 914 features, 48 features excluding ICD-10, and 866 features with ICD-10 only. Our model outperformed the traditional methods such as inclusive SRR, exclusive SRR, EMR-ISS, and KTAS. Figure 5 shows the AUROC curves for our model and 4 traditional models. The calculated inclusive SRR and exclusive SRR are shown in Table S6 in Multimedia Appendix 1. Finally, the model using the top 71 features from the AdaBoost also provided a lower balanced accuracy of 0.8245 and AUROC of 0.9194, similar to the cross-validation results.

**Table 4:** Comparison of the prediction performances of the prediction models on the test data set.

Model		Sensitivity	Specificity	Accuracy	Balanced accuracy	AUROC^a^
**Using all 914 features (including ICD-10^b^)**						
	Proposed model (DNN^c^)	0.8768	0.8625	0.8628	0.8697	0.9507
	AdaBoost^d^	0.8619	0.8655	0.8654	0.8637	0.9433
	XGBoost^e^	0.8292	0.8660	0.8653	0.8476	0.9331
	LightGBM^f^	0.8601	0.8365	0.8369	0.8483	0.9332
**Using 866 features (ICD-10 only)**						
	DNN	0.8365	0.8159	0.8162	0.8262	0.8964
	AdaBoost	0.7896	0.8319	0.8312	0.8108	0.8773
	XGBoost	0.7660	0.8348	0.8336	0.8004	0.8564
	LightGBM	0.7729	0.8285	0.8276	0.8007	0.8565
**Using 48 features (excluding ICD-10)**						
	DNN	0.8347	0.8784	0.8776	0.8565	0.9383
	AdaBoost	0.8354	0.8660	0.8655	0.8507	0.9363
	XGBoost	0.8339	0.8565	0.8561	0.8452	0.9318
	LightGBM	0.8299	0.8597	0.8592	0.8448	0.9318
**Traditional methods**						
	Inclusive SRR^g^	0.8964	0.6831	0.8926	0.7898	0.8699
	Exclusive SRR	0.8733	0.7078	0.8703	0.7905	0.8224
	EMR-ISS^h^	0.7874	0.7231	0.7863	0.7552	0.6171
	KTAS^i^	0.9359	0.0121	0.9178	0.4740	0.1841
**Others**						
	DNN using top 71 features from AdaBoost	0.7129	0.9362	0.9322	0.8245	0.9194

^a^AUROC: area under the receiver operating characteristic.

^b^ICD-10: International Classification of Disease 10th Revision.

^c^DNN: deep neural network.

^d^AdaBoost: Adaptive Boosting.

^e^XGBoost: Extreme Gradient Boosting.

^f^LightGBM: light gradient boosting machine.

^g^SRR: survival risk ratio.

^h^EMR-ISS: Mortality Ratio-adjusted Injury Severity Score.

^i^KTAS: Korean Triage and Acuity Scale.

### AI-Driven Public Website Development

We deployed our AI on a public website [27] to allow public access to the mortality prediction results in trauma patients (Figure S1 in Multimedia Appendix 1). Figure S1(a) shows a user's web interface to enter information. A user inputs age, gender, intentionality, injury mechanism, emergent symptoms, AVPU scale, initial KTAS, altered KTAS, torso procedures (chest, abdomen, vascular, and heart), head surgery, ECMO, and ICD-10 codes. Especially for ICD codes, a user can input multiple codes with a comma (eg, S072, S224, T083). As shown in Figure S1(b), after entering information in the web application, the user can immediately obtain the mortality results. The prediction results also include the probability of mortality.

## Discussion

### Principal Findings

Our AI model outperformed traditional ICD-10-based models and KTAS. Traditional methods produced high sensitivity and low specificity, with substantial bias in predicting mortality. Prediction performance was optimal when using all features, including ICD-10, as input features. The similarity between the cross-validation result and the testing data set indicates that overfitting or underfitting was minimal. In terms of ranked normalized feature importance, gender had the highest value, followed by age, coma, femur fracture, stupor, slip down, rib fracture, and head procedure. We used a population-based data set from all types of ED in South Korea, producing more robust and reliable results. To the best of our knowledge, our study is the first to demonstrate an AI model that drastically outperforms conventional ICD-based models and triage scales using a population-based data set. Our future goal is to construct a more comprehensive model incorporating both NEDIS-based and AIS-based AI [17].

Our proposed AI model has several advantages in clinical practice. First, a specialist is not required for AIS coding, so our AI model does not require additional burden. Second, our AI model demonstrates the ability to augment the KTAS provider's decision. Third, the feature importance used may benefit clinical decision-making and future research. Deep learning is generally considered a “black box,” hence the feature importance analysis based on a machine learning algorithm provides meaningful insight to clinicians and researchers. Finally, we aspire for the global application of our model and have produced a publicly available web application for hospitals to utilize for the benefit of the entire trauma system [28,29].

Currently, ISS and ICISS are the most popular risk estimation models of trauma-related mortality. More complex models containing physiologic and demographic parameters are available [2,4,5,7], but none supersedes ISS or ICISS [1,9]. ISS is simple to use, but AIS coding is time consuming and expensive, whereas ICISS utilizes diagnostic code to claim charges. Therefore, ICISS is more useful for population-based data sets than ISS [8]. The results from ICISS in our study were comparable to those from previous studies [26,30]. We also applied EMR-ISS to the NEDIS data set, which showed good performance in a previous study [11] but poor accuracy here.

Recently, several AI models were proposed to predict trauma-related mortality. Previously, in a multicenter retrospective study in South Korea, we investigated a deep learning model using the AIS code for predicting mortality [17]. We reanalyzed the ISS system and redefine 46 new regions to discriminate the risk among different internal organs. The DNN with 46 features from the 46 new regions produced the highest accuracy. We found that the AI model can augment the performance of the AIS system. Recently, Tran et al [18] reported a machine-learning model that predicted trauma-related mortality using ICD-10. The authors used the NTDB data set and compared machine learning with ISS and TMPM10 [10], an ICD-10-based metric. However, the accuracy of each model was comparable. In this study, our AI model drastically outperformed ICISS and EMR-ISS. Kwon et al [31], in a retrospective observational study using a NEDIS data set including trauma and nontrauma patients, reported a deep learning-based model that showed a higher accuracy than KTAS for predicting in-hospital mortality. To the best of our knowledge, our AI model is the most accurate model and outperforms both diagnostic code-based metrics and triage scales in trauma patients.

### Limitations and Future Works

Our study has several limitations. First, this is a retrospective study and may induce substantial selection and survival bias; further prospective trials and validation are needed. Second, we used procedure codes as 1 of the input features. However, they are not practically available during ED admission. Thus, in a prospective study, unconfirmed procedure codes may be used for predicting in-hospitality mortality. Third, in this study, we did not consider physiological signals, such as blood pressure, heart rate, and body temperature. We tried to train and develop an AI model using the information of physiological signals. However, the model’s performance was poor because limited physiological signals were recorded in NEDIS; only blood pressure, heart rate, and temperature values at the time of admission were recorded. We believe that time-series physiological signals, such as electrocardiogram, photoplethysmogram, and blood pressure waveform, could improve our proposed model. Fourth, due to the structure of the NEDIS data set, some data, such as age, are collected as categorized data instead of continuous data. Thus, our proposed AI model could enhance the prediction performance with age as a continuous value. Fifth, some categorized input variables in the injury mechanism may appear inappropriate. For instance, the term “traffic accident-pedestrian, train, airplane, ship, etc” is considered 1 variable. However, pedestrians are not associated with an airplane and a ship. In addition, pedestrians have the highest mortality in road traffic collisions. Thus, the term should be separated into multiple variables. In future work, we plan to separate the variable into multiple categories and investigate the impact of each category. Sixth, we could not compare the prediction performances from our AI model with those from AIS code-based approaches such as ISS and NISS, as NEDIS does not provide AIS codes. Recently, we presented an AI model using AIS codes to predict in-hospital mortality [17]. The model outperformed conventional methods such as ISS and NISS for all accuracy metrics of sensitivity, specificity, balanced accuracy, and AUROC. As in the previous study, this study used ICD-10 and several clinical features instead of AIS codes and showed that the AI model outperformed conventional methods. Our goal is to construct a more comprehensive model incorporating both NEDIS-based and AIS-based AI models. Finally, our data did not include other races or data from other countries. Currently, our public website includes the following text: “This AI model was trained and evaluated from Korean trauma patients and may not be applicable to patients in other countries.” Thus, future external validation is warranted, wherein we consider using global data to further improve our proposed AI model.

### Conclusions

Our proposed AI model shows high accuracy and outperforms traditional diagnostic code-based prediction models and triage scales. We believe that our population-based AI model can facilitaite better understanding and practice in physical trauma care. Moreover, this AI and data-driven prediction model may minimize the bias and workload of humans. However, future external validation and prospective studies are warranted to prove the true effect size.

## Appendix

Multimedia Appendix 1 Tables S1 to S6 and Figure S1.

## Abbreviations

AdaBoost: Adaptive Boosting
AI: artificial intelligence
AIS: Abbreviated Injury Scale
AUROC: area under the receiver operating characteristic
CTAS: Canadian Triage and Acuity Scale
DNN: deep neural network
ECMO: extracorporeal membrane oxygenation
ED: emergency department
EDNN: ensemble of deep neural networks
EMR-ISS: mortality ratio-adjusted Injury Severity Score
FC: fully connected
GCS: Glasgow Coma Scale
ICD: International Classification of Diseases
ICD-10: International Classification of Diseases 10th Revision
ICISS: International Classification of Diseases–based Injury Severity Score
ICU: intensive care unit
ISS: Injury Severity Score
KTAS: Korean Triage and Acuity Scale
LightGBM: light gradient boosting machine
NEDIS: National Emergency Department Information System
NTDB: National Trauma Database
Ps: probability of survival
SMOTE: Synthetic Minority Oversampling Technique
SRR: survival risk ratio
TMPM-ICD10: trauma mortality models using ICD-10
XGBoost: Extreme Gradient Boosting

## Data Availability

The data are not publicly available due to restrictions from National Emergency Department Information System (NEDIS) policy and belong to the National Emergency Medical Center (NEMC) of Korea. NEMC provides deidentified NEDIS data to researchers for nonprofit academic research. Details for accessing the raw data and guide are available on the NEMC website.

## References

[1] Baker SP, O'Neill B, Haddon W, Long WB (1974). The Injury Severity Score: a method for describing patients with multiple injuries and evaluating emergency care. J Trauma.

[2] Champion HR, Copes WS, Sacco WJ, Lawnick MM, Bain LW, Gann DS, Gennarelli T, Mackenzie E, Schwaitzberg S (1990). A new characterization of injury severity. J Trauma.

[3] Kuo SCH, Kuo P, Chen Y, Chien P, Hsieh H, Hsieh C (2017). Comparison of the new Exponential Injury Severity Score with the Injury Severity Score and the New Injury Severity Score in trauma patients: A cross-sectional study. PLoS One.

[4] Macleod J, Kobusingye O, Frost C, Lett R (2007). Kampala Trauma Score (KTS): is it a new triage tool?. East Cent Afr J Surg.

[5] Moore L, Hanley JA, Turgeon AF, Lavoie A, Eric B (2010). A new method for evaluating trauma centre outcome performance: TRAM-adjusted mortality estimates. Ann Surg.

[6] Moore L, Lavoie A, Le Sage N, Bergeron E, Emond M, Abdous B (2008). Consensus or data-derived anatomic injury severity scoring?. J Trauma.

[7] West TA, Rivara FP, Cummings P, Jurkovich GJ, Maier RV (2000). Harborview assessment for risk of mortality: an improved measure of injury severity on the basis of ICD-9-CM. J Trauma.

[8] Gagné M, Moore L, Sirois M-J, Simard M, Beaudoin C, Kuimi BLB (2017). Performance of International Classification of Diseases-based injury severity measures used to predict in-hospital mortality and intensive care admission among traumatic brain-injured patients. J Trauma Acute Care Surg.

[9] Bergeron E, Simons R, Linton C, Yang F, Tallon JM, Stewart TC, de Guia N, Stephens M (2007). Canadian benchmarks in trauma. J Trauma.

[10] Osler TM, Glance LG, Cook A, Buzas JS, Hosmer DW (2019). A trauma mortality prediction model based on the ICD-10-CM lexicon: TMPM-ICD10. J Trauma Acute Care Surg.

[11] Kim J, Shin SD, Im TH, Kug JL, Ko SB, Park JO, Ahn KO, Song KJ (2009). Development and validation of the Excess Mortality Ratio-adjusted Injury Severity Score using the International Classification of Diseases 10th Edition. Acad Emerg Med.

[12] Ryu J, Min M, Lee D, Yeom S, Lee S, Wang I, Cho S, Hwang S, Lee J, Kim Y (2019). Changes in relative importance of the 5-Level Triage System, Korean Triage and Acuity Scale, for the disposition of emergency patients induced by forced reduction in its level number: a multi-center registry-based retrospective cohort study. J Korean Med Sci.

[13] Lee JH, Park YS, Park IC, Lee HS, Kim JH, Park JM, Chung SP, Kim MJ (2019). Over-triage occurs when considering the patient's pain in Korean Triage and Acuity Scale (KTAS). PLoS One.

[14] Korndorffer JR, Hawn MT, Spain DA, Knowlton LM, Azagury DE, Nassar AK, Lau JN, Arnow KD, Trickey AW, Pugh CM (2020). Situating artificial intelligence in surgery: a focus on disease severity. Ann Surg.

[15] Madani A, Namazi B, Altieri MS, Hashimoto DA, Rivera AM, Pucher PH, Navarrete-Welton A, Sankaranarayanan G, Brunt LM, Okrainec A, Alseidi A (2022). Artificial intelligence for intraoperative guidance: using semantic segmentation to identify surgical anatomy during laparoscopic cholecystectomy. Ann Surg.

[16] Hashimoto DA, Rosman G, Rus D, Meireles OR (2018). Artificial intelligence in surgery: promises and perils. Ann Surg.

[17] Kang WS, Chung H, Ko H, Kim NY, Kim DW, Cho J, Shim H, Kim JG, Jang JY, Kim KW, Lee J (2021). Artificial intelligence to predict in-hospital mortality using novel anatomical injury score. Sci Rep.

[18] Tran Z, Zhang W, Verma A, Cook A, Kim D, Burruss S, Ramezani R, Benharash P (2022). The derivation of an International Classification of Diseases, Tenth Revision-based trauma-related mortality model using machine learning. J Trauma Acute Care Surg.

[19] Moons KGM, Altman DG, Reitsma JB, Ioannidis JPA, Macaskill P, Steyerberg EW, Vickers AJ, Ransohoff DF, Collins GS (2015). Transparent reporting of a multivariable prediction model for Individual Prognosis or Diagnosis (TRIPOD): explanation and elaboration. Ann Intern Med.

[20] Kelly CA, Upex A, Bateman DN (2004). Comparison of consciousness level assessment in the poisoned patient using the alert/verbal/painful/unresponsive scale and the Glasgow Coma Scale. Ann Emerg Med.

[21] McNarry AF, Goldhill DR (2004). Simple bedside assessment of level of consciousness: comparison of two simple assessment scales with the Glasgow Coma scale. Anaesthesia.

[22] Chawla NV, Bowyer KW, Hall LO, Kegelmeyer WP (2002). SMOTE: Synthetic Minority Over-sampling Technique. JAIR.

[23] Mathanker S, Weckler P, Bowser T, Wang N, Maness N (2011). AdaBoost classifiers for pecan defect classification. Comput Electron Agric.

[24] Chen T, Guestrin C (2016). XGBoost: a scalable tree boosting system.

[25] Ke G, Meng Q, Finley T, Wang T, Chen W, Ma W (2017). LightGBM: a highly efficient gradient boosting decision tree.

[26] Kim Y, Jung KY (2003). Utility of the international classification of diseases injury severity score: detecting preventable deaths and comparing the performance of emergency medical centers. J Trauma.

[27] In-hospital mortality prediction of trauma patients. AI Model.

[28] Lee HS, Sung WY, Lee JY, Lee WS, Seo SW (2021). Comparative evaluation of emergency medical service trauma patient transportation patterns before and after level 1 regional trauma center establishment: a retrospective single-center study. J Trauma Inj.

[29] Park Y, Lee GJ, Lee MA, Choi KK, Gwak J, Hyun SY, Jeon YB, Yoon Y, Lee J, Yu B (2021). Major causes of preventable death in trauma patients. J Trauma Inj.

[30] Berecki-Gisolf J, Tharanga Fernando D, D'Elia A (2022). International Classification of Disease-based Injury Severity Score (ICISS): a data linkage study of hospital and death data in Victoria, Australia. Injury.

[31] Kwon J, Lee Y, Lee Y, Lee S, Park H, Park J (2018). Validation of deep-learning-based triage and acuity score using a large national dataset. PLoS ONE.

