# Supplementary material for: Model for Predicting In-Hospital Mortality of Physical Trauma Patients Using Artificial Intelligence Techniques: Nationwide Population-Based Study in Korea
Source: J Med Internet Res. 2022 Dec 13;24(12):e43757. doi: 10.2196/43757 (PMC9795391; doi:10.2196/43757)
Supplement: Multimedia Appendix 1 [file jmir_v24i12e43757_app1.docx]

| **Table S1. Categorization of procedure code** | | |
| --- | --- | --- |
| **Code** | **Procedure code name** | **Categorization** |
| **Head** |  |  |
| N0322 | Burr Hole Drainage (SDH, EDH) | head procedure |
| N0323 | Burr Hole Drainage (intracerebral) | head procedure |
| N0324 | Burr Hole or Trephination (Others) | head procedure |
| N0331 | Craniotomy or Craniectomy (diagnostic) | head procedure |
| N0333 | Craniotomy or Craniectomy (decompression) | head procedure |
| S4601 | Neurorrhaphy (extracranial cerebral nerve) | head procedure |
| S4621 | Intracranial Hematoma Removal (SDH or EDH) | head procedure |
| S4622 | Intracerebral Hematoma Removal | head procedure |
| S4756 | CNS stereotactic OP (biopsy, suction, hematoma removal) | head procedure |
| **Abdomen** |  |  |
| M6611 | TEVAR (Aortic) Percutaneous | torso procedure-vascular |
| M6613 | EVAR (others) Percutaneous | torso procedure-vascular |
| M6644 | Arterial Embolization | torso procedure-vascular |
| O2033 | Resection of Aneurismectomy-Descending Thoracic Aorta | torso procedure-vascular |
| O2056 | Thrombectomy (Artery), Others | torso procedure-vascular |
| O2072 | Vessel Ligation (Following Laparotomy) | torso procedure-vascular |
| O2073 | Vessel Ligation (Others) | torso procedure-vascular |
| OA632 | Angioplasty with End-to-End Anastomosis (by Laparotomy) | torso procedure-vascular |
| OA637 | Angioplasty with Patch Graft (by Laparotomy - Autologous Vessel) | torso procedure-vascular |
| OA638 | Angioplasty with Patch Graft (Others - Artificial Vessel) | torso procedure-vascular |
| P2091 | Splenectomy - Total | torso procedure-abdomen |
| P2094 | Splenorrhaphy | torso procedure-abdomen |
| Q2440 | Exploratory Laparotomy | torso procedure-abdomen |
| Q2445 | Damage Control Laparotomy | torso procedure-abdomen |
| Q2540 | Simple Closure of Perforated Stomach | torso procedure-abdomen |
| Q2572 | Gastroenterostomy (Gastrojejunostomy) | torso procedure-abdomen |
| Q2573 | Gastroenterostomy (Roux-en-Y Gastrojejunostomy) | torso procedure-abdomen |
| Q0259 | Subtotal Gastrectomy | torso procedure-abdomen |
| Q0251 | Subtotal Gastrectomy (Partial) | torso procedure-abdomen |
| Q0253 | Subtotal Gastrectomy (Distal) | torso procedure-abdomen |
| Q0255 | Subtotal Gastrectomy (Pylorus preserving) | torso procedure-abdomen |
| Q0257 | Subtotal Gastrectomy (Wedge Resection) | torso procedure-abdomen |
| Q2601 | Esophagojejunostomy (including Roux-en-Y and loop) | torso procedure-abdomen |
| Q2650 | Small Bowel Segmental Resection (without lymphadenectomy) | torso procedure-abdomen |
| Q2651 | Small Bowel Segmental Resection (with lymphadenectomy) | torso procedure-abdomen |
| Q2671 | Hemicolectomy (Right or left) - without lymphadenectomy | torso procedure-abdomen |
| Q2673 | Colectomy (Segmental resection) - without lymphadenectomy | torso procedure-abdomen |
| Q2679 | Hartmann's operation (without lymphadenectomy) | torso procedure-abdomen |
| Q1262 | Subtotal Colectomy (without lymphadenectomy) | torso procedure-abdomen |
| Q2672 | Total Colectomy (without lymphadenectomy) | torso procedure-abdomen |
| Q2921 | Anterior Resection of Rectal and Sigmoid (without lymphadenectomy) | torso procedure-abdomen |
| Q2922 | Low Anterior Resection of Rectal and Sigmoid (without lymphadenectomy) | torso procedure-abdomen |
| Q2925 | Total Coloproctectomy with ileostomy (without lymphadenectomy) | torso procedure-abdomen |
| Q2680 | Intestinal Anastomosis | torso procedure-abdomen |
| Q2771 | Repair of Mesenteric injury - with bowel resection | torso procedure-abdomen |
| Q2773 | Closure of Perforation | torso procedure-abdomen |
| Q2774 | Repair of Mesenteric injury - without bowel resection | torso procedure-abdomen |
| Q2775 | Repair of Mesenteric injury - both mesenteric repair and bowel resection | torso procedure-abdomen |
| Q2791 | Enterostomy - Tube | torso procedure-abdomen |
| Q2792 | Enterostomy - Loop | torso procedure-abdomen |
| Q2793 | Enterostomy - End | torso procedure-abdomen |
| Q7221 | Hepatectomy - Wedge Resection | torso procedure-abdomen |
| Q7222 | Hepatectomy - Segmentectomy | torso procedure-abdomen |
| Q7240 | Suture of Liver laceration | torso procedure-abdomen |
| Q7380 | Cholecystectomy | torso procedure-abdomen |
| Q7511 | Drainage of Pancreatic Abscess | torso procedure-abdomen |
| Q7520 | Closure of Pancreas | torso procedure-abdomen |
| Q7563 | Pancreatectomy - Subtotal Pancreatectomy | torso procedure-abdomen |
| Q7565 | Pancreatectomy - Distal Pancreatectomy | torso procedure-abdomen |
| QA671 | Hemicolectomy (Right or left) - with lymphadenectomy | torso procedure-abdomen |
| QA672 | Total Colectomy (with lymphadenectomy) | torso procedure-abdomen |
| QA673 | Colectomy (Segmental resection) - with lymphadenectomy | torso procedure-abdomen |
| R3271 | Simple Total Nephrectomy | torso procedure-abdomen |
| R3290 | Partial Nephrectomy | torso procedure-abdomen |
| R3550 | Repair of Bladder Rupture | torso procedure-abdomen |
| **Chest** |  |  |
| N0532 | Open Reduction of Rib Fracture | torso procedure-chest |
| O1360 | Exploratory Thoracotomy | torso procedure-chest |
| O1401 | Wedge Resection of Lung - Single | torso procedure-chest |
| O1403 | Wedge Resection of Lung - 2 or 3 | torso procedure-chest |
| O1421 | Lobectomy of Lung - Single | torso procedure-chest |
| O1440 | Repair of Lung | torso procedure-chest |
| O1450 | Pleural Decortication | torso procedure-chest |
| O1510 | Closed thoracostomy | torso procedure-chest |
| O1541 | Open Reduction of Sternum fracture | torso procedure-chest |
| O1600 | Repair of Diaphragm | torso procedure-chest |
| O1660 | Repair of Cardiac Wound | torso procedure-heart |
| O1890 | Extracorporeal Circulation by Heart-Lung Machine | torso procedure-heart |
| O2071 | Vessel Ligation - following Thoracotomy | torso procedure-vascular |
| O2073 | Vessel Ligation - Others | torso procedure-vascular |
| O1903 | Extracorporeal Membrane Oxygenation (procedure day) | ECMO |
| O1904 | Extracorporeal Membrane Oxygenation (from 1 day after procedure) | ECMO |
| SDH, subdural hemorrhage; EDH, epidural hemorrhage; TEVAR. thoracic endovascular aortic repair; EVAR, endovascular aneurysm repair | | |

| **Table S2. Summary of training, validation, and testing datasets** | | | |
| --- | --- | --- | --- |
|  | **Deceased group** | **Survived group** | **Total** |
| **Training data** | 11,007 | 611,481 | 622,488 |
| **Testing data** | 2,753 | 152,870 | 155,623 |
| **Total** | 13,760 | 764,351 | 778,111 |

| **Table S3. Comparison of ICD-10 between deceased and survived patients** | | | | |
| --- | --- | --- | --- | --- |
| **no** | **ICD-10 code** | **Deceased (N=13760)** | **Survived (N=764351)** | **p** |
| 1 | T000 | 1 (0%) | 34 (0%) | 1.000 |
| 2 | S001 | 10 (0.1%) | 1878 (0.2%) | <.001 |
| 3 | T001 | 0 (0%) | 8 (0%) | 1.000 |
| 4 | S002 | 2 (0%) | 585 (0.1%) | 0.014 |
| 5 | T002 | 0 (0%) | 28 (0%) | 1.000 |
| 6 | S003 | 2 (0%) | 893 (0.1%) | <.001 |
| 7 | T003 | 0 (0%) | 43 (0%) | 0.763 |
| 8 | S004 | 3 (0%) | 315 (0%) | 0.366 |
| 9 | S005 | 3 (0%) | 784 (0.1%) | 0.005 |
| 10 | T006 | 0 (0%) | 70 (0%) | 0.503 |
| 11 | S007 | 4 (0%) | 430 (0.1%) | 0.247 |
| 12 | S008 | 144 (1%) | 16355 (2.1%) | <.001 |
| 13 | T008 | 1 (0%) | 680 (0.1%) | 0.002 |
| 14 | S009 | 54 (0.4%) | 3351 (0.4%) | 0.457 |
| 15 | T009 | 365 (2.7%) | 32096 (4.2%) | <.001 |
| 16 | S010 | 360 (2.6%) | 14788 (1.9%) | <.001 |
| 17 | T010 | 1 (0%) | 20 (0%) | 0.831 |
| 18 | S011 | 46 (0.3%) | 6289 (0.8%) | <.001 |
| 19 | T011 | 0 (0%) | 11 (0%) | 1.000 |
| 20 | S012 | 11 (0.1%) | 1619 (0.2%) | 0.001 |
| 21 | T012 | 0 (0%) | 34 (0%) | 0.895 |
| 22 | S013 | 29 (0.2%) | 1527 (0.2%) | 0.850 |
| 23 | T013 | 2 (0%) | 27 (0%) | 0.164 |
| 24 | S014 | 25 (0.2%) | 2690 (0.4%) | 0.001 |
| 25 | S015 | 37 (0.3%) | 5276 (0.7%) | <.001 |
| 26 | T016 | 0 (0%) | 16 (0%) | 1.000 |
| 27 | S017 | 17 (0.1%) | 1040 (0.1%) | 0.781 |
| 28 | S018 | 234 (1.7%) | 11643 (1.5%) | 0.100 |
| 29 | T018 | 1 (0%) | 29 (0%) | 1.000 |
| 30 | S019 | 26 (0.2%) | 1196 (0.2%) | 0.398 |
| 31 | T019 | 47 (0.3%) | 870 (0.1%) | <.001 |
| 32 | S020 | 1138 (8.3%) | 7570 (1%) | <.001 |
| 33 | T020 | 1 (0%) | 2 (0%) | 0.050 |
| 34 | S021 | 1027 (7.5%) | 8468 (1.1%) | <.001 |
| 35 | T021 | 1 (0%) | 105 (0%) | 0.783 |
| 36 | S022 | 204 (1.5%) | 11410 (1.5%) | 0.950 |
| 37 | T022 | 0 (0%) | 13 (0%) | 1.000 |
| 38 | S023 | 148 (1.1%) | 7036 (0.9%) | 0.066 |
| 39 | T023 | 0 (0%) | 22 (0%) | 1.000 |
| 40 | S024 | 378 (2.7%) | 10051 (1.3%) | <.001 |
| 41 | T024 | 0 (0%) | 2 (0%) | 1.000 |
| 42 | S025 | 18 (0.1%) | 2916 (0.4%) | <.001 |
| 43 | T025 | 1 (0%) | 15 (0%) | 0.681 |
| 44 | S026 | 124 (0.9%) | 5572 (0.7%) | 0.022 |
| 45 | T026 | 1 (0%) | 18 (0%) | 0.775 |
| 46 | S027 | 119 (0.9%) | 887 (0.1%) | <.001 |
| 47 | T027 | 0 (0%) | 16 (0%) | 1.000 |
| 48 | S028 | 292 (2.1%) | 7427 (1%) | <.001 |
| 49 | T028 | 6 (0%) | 62 (0%) | <.001 |
| 50 | S029 | 624 (4.5%) | 4073 (0.5%) | <.001 |
| 51 | T029 | 11 (0.1%) | 37 (0%) | <.001 |
| 52 | S030 | 3 (0%) | 43 (0%) | 0.059 |
| 53 | T030 | 0 (0%) | 24 (0%) | 1.000 |
| 54 | S031 | 0 (0%) | 46 (0%) | 0.726 |
| 55 | T031 | 0 (0%) | 91 (0%) | 0.378 |
| 56 | S032 | 16 (0.1%) | 2126 (0.3%) | <.001 |
| 57 | T032 | 0 (0%) | 6 (0%) | 1.000 |
| 58 | S033 | 0 (0%) | 1 (0%) | 1.000 |
| 59 | T033 | 0 (0%) | 8 (0%) | 1.000 |
| 60 | S034 | 0 (0%) | 392 (0.1%) | 0.014 |
| 61 | T034 | 0 (0%) | 1 (0%) | 1.000 |
| 62 | S035 | 0 (0%) | 34 (0%) | 0.895 |
| 63 | T038 | 0 (0%) | 83 (0%) | 0.420 |
| 64 | T039 | 0 (0%) | 204 (0%) | 0.099 |
| 65 | S040 | 4 (0%) | 300 (0%) | 0.703 |
| 66 | T040 | 0 (0%) | 1 (0%) | 1.000 |
| 67 | S041 | 3 (0%) | 63 (0%) | 0.213 |
| 68 | T041 | 0 (0%) | 6 (0%) | 1.000 |
| 69 | S042 | 1 (0%) | 39 (0%) | 1.000 |
| 70 | T042 | 1 (0%) | 33 (0%) | 1.000 |
| 71 | S043 | 0 (0%) | 53 (0%) | 0.649 |
| 72 | T043 | 3 (0%) | 54 (0%) | 0.134 |
| 73 | S044 | 0 (0%) | 19 (0%) | 1.000 |
| 74 | T044 | 0 (0%) | 2 (0%) | 1.000 |
| 75 | S045 | 1 (0%) | 375 (0%) | 0.044 |
| 76 | S046 | 0 (0%) | 23 (0%) | 1.000 |
| 77 | T047 | 0 (0%) | 1 (0%) | 1.000 |
| 78 | S048 | 0 (0%) | 28 (0%) | 1.000 |
| 79 | T048 | 0 (0%) | 3 (0%) | 1.000 |
| 80 | S049 | 4 (0%) | 29 (0%) | <.001 |
| 81 | T049 | 2 (0%) | 13 (0%) | 0.016 |
| 82 | S050 | 8 (0.1%) | 946 (0.1%) | 0.040 |
| 83 | T050 | 0 (0%) | 3 (0%) | 1.000 |
| 84 | S051 | 19 (0.1%) | 3244 (0.4%) | <.001 |
| 85 | T051 | 0 (0%) | 7 (0%) | 1.000 |
| 86 | S052 | 4 (0%) | 675 (0.1%) | 0.029 |
| 87 | T052 | 0 (0%) | 1 (0%) | 1.000 |
| 88 | S053 | 6 (0%) | 1539 (0.2%) | <.001 |
| 89 | T053 | 0 (0%) | 1 (0%) | 1.000 |
| 90 | S054 | 0 (0%) | 123 (0%) | 0.252 |
| 91 | T054 | 0 (0%) | 2 (0%) | 1.000 |
| 92 | S055 | 0 (0%) | 634 (0.1%) | 0.001 |
| 93 | T055 | 2 (0%) | 2 (0%) | <.001 |
| 94 | S056 | 3 (0%) | 367 (0%) | 0.230 |
| 95 | T056 | 0 (0%) | 1 (0%) | 1.000 |
| 96 | S057 | 0 (0%) | 12 (0%) | 1.000 |
| 97 | S058 | 4 (0%) | 1651 (0.2%) | <.001 |
| 98 | T058 | 0 (0%) | 1 (0%) | 1.000 |
| 99 | S059 | 4 (0%) | 254 (0%) | 0.976 |
| 100 | T059 | 0 (0%) | 10 (0%) | 1.000 |
| 101 | S060 | 289 (2.1%) | 87315 (11.4%) | <.001 |
| 102 | T060 | 2 (0%) | 8 (0%) | 0.001 |
| 103 | S061 | 298 (2.2%) | 243 (0%) | <.001 |
| 104 | T061 | 2 (0%) | 24 (0%) | 0.122 |
| 105 | S062 | 1342 (9.8%) | 7642 (1%) | <.001 |
| 106 | T062 | 0 (0%) | 4 (0%) | 1.000 |
| 107 | S063 | 629 (4.6%) | 6371 (0.8%) | <.001 |
| 108 | T063 | 0 (0%) | 2 (0%) | 1.000 |
| 109 | S064 | 757 (5.5%) | 8562 (1.1%) | <.001 |
| 110 | T064 | 0 (0%) | 47 (0%) | 0.714 |
| 111 | S065 | 5977 (43.4%) | 33901 (4.4%) | <.001 |
| 112 | T065 | 1 (0%) | 1 (0%) | 0.013 |
| 113 | S066 | 3133 (22.8%) | 15360 (2%) | <.001 |
| 114 | S067 | 48 (0.3%) | 15 (0%) | <.001 |
| 115 | S068 | 966 (7%) | 4957 (0.6%) | <.001 |
| 116 | T068 | 71 (0.5%) | 745 (0.1%) | <.001 |
| 117 | S069 | 118 (0.9%) | 374 (0%) | <.001 |
| 118 | S070 | 5 (0%) | 130 (0%) | 0.168 |
| 119 | S071 | 2 (0%) | 15 (0%) | 0.027 |
| 120 | S078 | 2 (0%) | 30 (0%) | 0.210 |
| 121 | S079 | 0 (0%) | 4 (0%) | 1.000 |
| 122 | S080 | 5 (0%) | 164 (0%) | 0.378 |
| 123 | T080 | 28 (0.2%) | 1065 (0.1%) | 0.061 |
| 124 | S081 | 2 (0%) | 63 (0%) | 0.741 |
| 125 | T081 | 0 (0%) | 2 (0%) | 1.000 |
| 126 | S088 | 0 (0%) | 15 (0%) | 1.000 |
| 127 | S090 | 4 (0%) | 65 (0%) | 0.037 |
| 128 | T090 | 0 (0%) | 155 (0%) | 0.172 |
| 129 | S091 | 5 (0%) | 1138 (0.1%) | <.001 |
| 130 | T091 | 0 (0%) | 58 (0%) | 0.600 |
| 131 | S092 | 3 (0%) | 244 (0%) | 0.675 |
| 132 | T092 | 2 (0%) | 513 (0.1%) | 0.027 |
| 133 | T093 | 53 (0.4%) | 677 (0.1%) | <.001 |
| 134 | T094 | 0 (0%) | 65 (0%) | 0.541 |
| 135 | T095 | 0 (0%) | 16 (0%) | 1.000 |
| 136 | S097 | 30 (0.2%) | 191 (0%) | <.001 |
| 137 | S098 | 73 (0.5%) | 1031 (0.1%) | <.001 |
| 138 | T098 | 0 (0%) | 4 (0%) | 1.000 |
| 139 | S099 | 43 (0.3%) | 1557 (0.2%) | 0.007 |
| 140 | T099 | 0 (0%) | 86 (0%) | 0.404 |
| 141 | S100 | 1 (0%) | 81 (0%) | 1.000 |
| 142 | T100 | 0 (0%) | 16 (0%) | 1.000 |
| 143 | S101 | 1 (0%) | 32 (0%) | 1.000 |
| 144 | T101 | 0 (0%) | 5 (0%) | 1.000 |
| 145 | S107 | 0 (0%) | 18 (0%) | 1.000 |
| 146 | S108 | 18 (0.1%) | 623 (0.1%) | 0.065 |
| 147 | S109 | 10 (0.1%) | 905 (0.1%) | 0.154 |
| 148 | S110 | 3 (0%) | 55 (0%) | 0.142 |
| 149 | T110 | 2 (0%) | 109 (0%) | 1.000 |
| 150 | S111 | 0 (0%) | 18 (0%) | 1.000 |
| 151 | T111 | 2 (0%) | 109 (0%) | 1.000 |
| 152 | S112 | 2 (0%) | 27 (0%) | 0.164 |
| 153 | T112 | 0 (0%) | 24 (0%) | 1.000 |
| 154 | T113 | 0 (0%) | 2 (0%) | 1.000 |
| 155 | T114 | 0 (0%) | 4 (0%) | 1.000 |
| 156 | T115 | 0 (0%) | 30 (0%) | 0.966 |
| 157 | T116 | 1 (0%) | 15 (0%) | 0.681 |
| 158 | S117 | 3 (0%) | 95 (0%) | 0.557 |
| 159 | S118 | 2 (0%) | 312 (0%) | 0.191 |
| 160 | T118 | 0 (0%) | 3 (0%) | 1.000 |
| 161 | S119 | 21 (0.2%) | 627 (0.1%) | 0.007 |
| 162 | T119 | 0 (0%) | 52 (0%) | 0.659 |
| 163 | S120 | 52 (0.4%) | 438 (0.1%) | <.001 |
| 164 | T120 | 0 (0%) | 52 (0%) | 0.659 |
| 165 | S121 | 88 (0.6%) | 1145 (0.1%) | <.001 |
| 166 | T121 | 2 (0%) | 15 (0%) | 0.027 |
| 167 | S122 | 124 (0.9%) | 2602 (0.3%) | <.001 |
| 168 | S127 | 99 (0.7%) | 996 (0.1%) | <.001 |
| 169 | S128 | 5 (0%) | 154 (0%) | 0.310 |
| 170 | S129 | 177 (1.3%) | 2428 (0.3%) | <.001 |
| 171 | S130 | 15 (0.1%) | 787 (0.1%) | 0.932 |
| 172 | T130 | 0 (0%) | 157 (0%) | 0.168 |
| 173 | S131 | 130 (0.9%) | 985 (0.1%) | <.001 |
| 174 | T131 | 6 (0%) | 248 (0%) | 0.631 |
| 175 | S132 | 0 (0%) | 4 (0%) | 1.000 |
| 176 | T132 | 1 (0%) | 69 (0%) | 1.000 |
| 177 | S133 | 0 (0%) | 29 (0%) | 0.986 |
| 178 | T133 | 0 (0%) | 6 (0%) | 1.000 |
| 179 | S134 | 125 (0.9%) | 103489 (13.5%) | <.001 |
| 180 | T134 | 0 (0%) | 12 (0%) | 1.000 |
| 181 | S135 | 0 (0%) | 15 (0%) | 1.000 |
| 182 | T135 | 0 (0%) | 72 (0%) | 0.489 |
| 183 | S136 | 8 (0.1%) | 2286 (0.3%) | <.001 |
| 184 | T136 | 5 (0%) | 9 (0%) | <.001 |
| 185 | T138 | 0 (0%) | 2 (0%) | 1.000 |
| 186 | T139 | 0 (0%) | 85 (0%) | 0.409 |
| 187 | S140 | 7 (0.1%) | 525 (0.1%) | 0.530 |
| 188 | T140 | 42 (0.3%) | 6071 (0.8%) | <.001 |
| 189 | S141 | 212 (1.5%) | 3075 (0.4%) | <.001 |
| 190 | T141 | 16 (0.1%) | 3690 (0.5%) | <.001 |
| 191 | S142 | 3 (0%) | 185 (0%) | 1.000 |
| 192 | T142 | 1 (0%) | 117 (0%) | 0.682 |
| 193 | S143 | 3 (0%) | 354 (0%) | 0.259 |
| 194 | T143 | 6 (0%) | 835 (0.1%) | 0.028 |
| 195 | S144 | 0 (0%) | 4 (0%) | 1.000 |
| 196 | T144 | 1 (0%) | 40 (0%) | 1.000 |
| 197 | S145 | 0 (0%) | 5 (0%) | 1.000 |
| 198 | T145 | 7 (0.1%) | 73 (0%) | <.001 |
| 199 | S146 | 4 (0%) | 53 (0%) | 0.012 |
| 200 | T146 | 4 (0%) | 1084 (0.1%) | <.001 |
| 201 | T147 | 1 (0%) | 282 (0%) | 0.114 |
| 202 | T148 | 0 (0%) | 9 (0%) | 1.000 |
| 203 | T149 | 0 (0%) | 101 (0%) | 0.332 |
| 204 | S150 | 18 (0.1%) | 53 (0%) | <.001 |
| 205 | T150 | 0 (0%) | 212 (0%) | 0.090 |
| 206 | S151 | 13 (0.1%) | 47 (0%) | <.001 |
| 207 | T151 | 0 (0%) | 77 (0%) | 0.456 |
| 208 | S152 | 2 (0%) | 43 (0%) | 0.426 |
| 209 | S153 | 5 (0%) | 36 (0%) | <.001 |
| 210 | S157 | 1 (0%) | 15 (0%) | 0.681 |
| 211 | S158 | 6 (0%) | 110 (0%) | 0.015 |
| 212 | T158 | 0 (0%) | 21 (0%) | 1.000 |
| 213 | S159 | 3 (0%) | 76 (0%) | 0.346 |
| 214 | T159 | 0 (0%) | 86 (0%) | 0.404 |
| 215 | S170 | 0 (0%) | 4 (0%) | 1.000 |
| 216 | T170 | 0 (0%) | 12 (0%) | 1.000 |
| 217 | T171 | 0 (0%) | 57 (0%) | 0.610 |
| 218 | T172 | 7 (0.1%) | 394 (0.1%) | 1.000 |
| 219 | T173 | 3 (0%) | 68 (0%) | 0.262 |
| 220 | T174 | 5 (0%) | 50 (0%) | <.001 |
| 221 | T175 | 6 (0%) | 185 (0%) | 0.244 |
| 222 | S178 | 0 (0%) | 6 (0%) | 1.000 |
| 223 | T178 | 6 (0%) | 31 (0%) | <.001 |
| 224 | S179 | 0 (0%) | 7 (0%) | 1.000 |
| 225 | T179 | 76 (0.6%) | 176 (0%) | <.001 |
| 226 | T180 | 0 (0%) | 19 (0%) | 1.000 |
| 227 | T181 | 7 (0.1%) | 2078 (0.3%) | <.001 |
| 228 | T182 | 0 (0%) | 479 (0.1%) | 0.006 |
| 229 | T183 | 0 (0%) | 218 (0%) | 0.085 |
| 230 | T184 | 0 (0%) | 91 (0%) | 0.378 |
| 231 | T185 | 0 (0%) | 180 (0%) | 0.129 |
| 232 | T188 | 0 (0%) | 25 (0%) | 1.000 |
| 233 | T189 | 3 (0%) | 731 (0.1%) | 0.008 |
| 234 | T190 | 0 (0%) | 16 (0%) | 1.000 |
| 235 | T191 | 0 (0%) | 23 (0%) | 1.000 |
| 236 | T192 | 0 (0%) | 16 (0%) | 1.000 |
| 237 | T193 | 1 (0%) | 1 (0%) | 0.013 |
| 238 | S197 | 2 (0%) | 10 (0%) | 0.005 |
| 239 | S198 | 5 (0%) | 87 (0%) | 0.023 |
| 240 | T198 | 0 (0%) | 12 (0%) | 1.000 |
| 241 | S199 | 8 (0.1%) | 242 (0%) | 0.139 |
| 242 | T199 | 0 (0%) | 7 (0%) | 1.000 |
| 243 | S200 | 0 (0%) | 37 (0%) | 0.847 |
| 244 | T200 | 2 (0%) | 170 (0%) | 0.754 |
| 245 | S201 | 0 (0%) | 27 (0%) | 1.000 |
| 246 | T201 | 1 (0%) | 135 (0%) | 0.556 |
| 247 | S202 | 150 (1.1%) | 28885 (3.8%) | <.001 |
| 248 | T202 | 75 (0.5%) | 2723 (0.4%) | <.001 |
| 249 | S203 | 2 (0%) | 187 (0%) | 0.642 |
| 250 | T203 | 30 (0.2%) | 102 (0%) | <.001 |
| 251 | S204 | 2 (0%) | 107 (0%) | 1.000 |
| 252 | T204 | 0 (0%) | 10 (0%) | 1.000 |
| 253 | T205 | 0 (0%) | 4 (0%) | 1.000 |
| 254 | T206 | 1 (0%) | 10 (0%) | 0.485 |
| 255 | S207 | 1 (0%) | 36 (0%) | 1.000 |
| 256 | T207 | 0 (0%) | 4 (0%) | 1.000 |
| 257 | S208 | 7 (0.1%) | 591 (0.1%) | 0.340 |
| 258 | S210 | 0 (0%) | 17 (0%) | 1.000 |
| 259 | T210 | 3 (0%) | 62 (0%) | 0.204 |
| 260 | S211 | 7 (0.1%) | 337 (0%) | 0.865 |
| 261 | T211 | 0 (0%) | 57 (0%) | 0.610 |
| 262 | S212 | 2 (0%) | 264 (0%) | 0.305 |
| 263 | T212 | 58 (0.4%) | 1737 (0.2%) | <.001 |
| 264 | T213 | 59 (0.4%) | 269 (0%) | <.001 |
| 265 | T214 | 0 (0%) | 1 (0%) | 1.000 |
| 266 | T215 | 0 (0%) | 4 (0%) | 1.000 |
| 267 | T216 | 1 (0%) | 7 (0%) | 0.336 |
| 268 | S217 | 0 (0%) | 29 (0%) | 0.986 |
| 269 | T217 | 0 (0%) | 1 (0%) | 1.000 |
| 270 | S218 | 0 (0%) | 75 (0%) | 0.469 |
| 271 | S219 | 4 (0%) | 311 (0%) | 0.647 |
| 272 | S220 | 436 (3.2%) | 27307 (3.6%) | 0.012 |
| 273 | T220 | 3 (0%) | 75 (0%) | 0.336 |
| 274 | S221 | 72 (0.5%) | 1480 (0.2%) | <.001 |
| 275 | T221 | 0 (0%) | 36 (0%) | 0.863 |
| 276 | S222 | 184 (1.3%) | 7555 (1%) | <.001 |
| 277 | T222 | 13 (0.1%) | 1428 (0.2%) | 0.017 |
| 278 | S223 | 194 (1.4%) | 15193 (2%) | <.001 |
| 279 | T223 | 34 (0.2%) | 279 (0%) | <.001 |
| 280 | S224 | 1666 (12.1%) | 48951 (6.4%) | <.001 |
| 281 | T224 | 0 (0%) | 1 (0%) | 1.000 |
| 282 | S225 | 90 (0.7%) | 516 (0.1%) | <.001 |
| 283 | T225 | 0 (0%) | 1 (0%) | 1.000 |
| 284 | T226 | 1 (0%) | 8 (0%) | 0.389 |
| 285 | T227 | 1 (0%) | 2 (0%) | 0.050 |
| 286 | S228 | 9 (0.1%) | 144 (0%) | <.001 |
| 287 | S229 | 1 (0%) | 60 (0%) | 1.000 |
| 288 | S230 | 0 (0%) | 30 (0%) | 0.966 |
| 289 | T230 | 3 (0%) | 158 (0%) | 1.000 |
| 290 | S231 | 7 (0.1%) | 97 (0%) | <.001 |
| 291 | T231 | 1 (0%) | 70 (0%) | 1.000 |
| 292 | S232 | 0 (0%) | 21 (0%) | 1.000 |
| 293 | T232 | 31 (0.2%) | 1897 (0.2%) | 0.654 |
| 294 | S233 | 8 (0.1%) | 7142 (0.9%) | <.001 |
| 295 | T233 | 36 (0.3%) | 374 (0%) | <.001 |
| 296 | S234 | 3 (0%) | 3285 (0.4%) | <.001 |
| 297 | T234 | 0 (0%) | 6 (0%) | 1.000 |
| 298 | S235 | 0 (0%) | 689 (0.1%) | <.001 |
| 299 | T235 | 0 (0%) | 1 (0%) | 1.000 |
| 300 | T236 | 0 (0%) | 10 (0%) | 1.000 |
| 301 | T237 | 0 (0%) | 3 (0%) | 1.000 |
| 302 | S240 | 0 (0%) | 24 (0%) | 1.000 |
| 303 | T240 | 1 (0%) | 97 (0%) | 0.858 |
| 304 | S241 | 13 (0.1%) | 210 (0%) | <.001 |
| 305 | T241 | 0 (0%) | 49 (0%) | 0.691 |
| 306 | S242 | 3 (0%) | 21 (0%) | 0.001 |
| 307 | T242 | 22 (0.2%) | 2258 (0.3%) | 0.005 |
| 308 | T243 | 42 (0.3%) | 415 (0.1%) | <.001 |
| 309 | S244 | 0 (0%) | 3 (0%) | 1.000 |
| 310 | T244 | 1 (0%) | 5 (0%) | 0.222 |
| 311 | S245 | 1 (0%) | 4 (0%) | 0.163 |
| 312 | T245 | 0 (0%) | 2 (0%) | 1.000 |
| 313 | S246 | 0 (0%) | 7 (0%) | 1.000 |
| 314 | T246 | 1 (0%) | 15 (0%) | 0.681 |
| 315 | T247 | 0 (0%) | 5 (0%) | 1.000 |
| 316 | S250 | 91 (0.7%) | 210 (0%) | <.001 |
| 317 | T250 | 2 (0%) | 54 (0%) | 0.605 |
| 318 | S251 | 6 (0%) | 24 (0%) | <.001 |
| 319 | T251 | 0 (0%) | 28 (0%) | 1.000 |
| 320 | S252 | 7 (0.1%) | 4 (0%) | <.001 |
| 321 | T252 | 15 (0.1%) | 1159 (0.2%) | 0.244 |
| 322 | S253 | 3 (0%) | 11 (0%) | <.001 |
| 323 | T253 | 23 (0.2%) | 269 (0%) | <.001 |
| 324 | S254 | 12 (0.1%) | 23 (0%) | <.001 |
| 325 | T254 | 0 (0%) | 2 (0%) | 1.000 |
| 326 | S255 | 12 (0.1%) | 57 (0%) | <.001 |
| 327 | T256 | 0 (0%) | 7 (0%) | 1.000 |
| 328 | S257 | 2 (0%) | 3 (0%) | <.001 |
| 329 | T257 | 0 (0%) | 3 (0%) | 1.000 |
| 330 | S258 | 13 (0.1%) | 56 (0%) | <.001 |
| 331 | S259 | 4 (0%) | 25 (0%) | <.001 |
| 332 | S260 | 49 (0.4%) | 146 (0%) | <.001 |
| 333 | T260 | 2 (0%) | 85 (0%) | 1.000 |
| 334 | T261 | 1 (0%) | 123 (0%) | 0.637 |
| 335 | T263 | 0 (0%) | 10 (0%) | 1.000 |
| 336 | T264 | 0 (0%) | 21 (0%) | 1.000 |
| 337 | T265 | 0 (0%) | 5 (0%) | 1.000 |
| 338 | T266 | 0 (0%) | 40 (0%) | 0.804 |
| 339 | T267 | 0 (0%) | 11 (0%) | 1.000 |
| 340 | S268 | 34 (0.2%) | 265 (0%) | <.001 |
| 341 | T268 | 0 (0%) | 4 (0%) | 1.000 |
| 342 | S269 | 29 (0.2%) | 311 (0%) | <.001 |
| 343 | T269 | 0 (0%) | 5 (0%) | 1.000 |
| 344 | S270 | 292 (2.1%) | 6017 (0.8%) | <.001 |
| 345 | T270 | 4 (0%) | 46 (0%) | 0.005 |
| 346 | S271 | 522 (3.8%) | 8077 (1.1%) | <.001 |
| 347 | T271 | 13 (0.1%) | 51 (0%) | <.001 |
| 348 | S272 | 524 (3.8%) | 6399 (0.8%) | <.001 |
| 349 | T272 | 11 (0.1%) | 51 (0%) | <.001 |
| 350 | S273 | 513 (3.7%) | 4493 (0.6%) | <.001 |
| 351 | T273 | 18 (0.1%) | 186 (0%) | <.001 |
| 352 | S274 | 6 (0%) | 12 (0%) | <.001 |
| 353 | T274 | 0 (0%) | 10 (0%) | 1.000 |
| 354 | S275 | 10 (0.1%) | 70 (0%) | <.001 |
| 355 | S276 | 1 (0%) | 54 (0%) | 1.000 |
| 356 | T276 | 0 (0%) | 3 (0%) | 1.000 |
| 357 | S277 | 1 (0%) | 21 (0%) | 0.858 |
| 358 | T277 | 1 (0%) | 5 (0%) | 0.222 |
| 359 | S278 | 99 (0.7%) | 629 (0.1%) | <.001 |
| 360 | S279 | 2 (0%) | 76 (0%) | 0.917 |
| 361 | S280 | 2 (0%) | 44 (0%) | 0.442 |
| 362 | T280 | 0 (0%) | 20 (0%) | 1.000 |
| 363 | T281 | 1 (0%) | 7 (0%) | 0.336 |
| 364 | T283 | 0 (0%) | 6 (0%) | 1.000 |
| 365 | T284 | 0 (0%) | 1 (0%) | 1.000 |
| 366 | T285 | 1 (0%) | 19 (0%) | 0.804 |
| 367 | T286 | 4 (0%) | 118 (0%) | 0.356 |
| 368 | T287 | 6 (0%) | 113 (0%) | 0.018 |
| 369 | T288 | 0 (0%) | 1 (0%) | 1.000 |
| 370 | T289 | 0 (0%) | 3 (0%) | 1.000 |
| 371 | S290 | 1 (0%) | 85 (0%) | 0.986 |
| 372 | T290 | 5 (0%) | 54 (0%) | <.001 |
| 373 | T291 | 0 (0%) | 3 (0%) | 1.000 |
| 374 | T292 | 11 (0.1%) | 243 (0%) | 0.004 |
| 375 | T293 | 53 (0.4%) | 176 (0%) | <.001 |
| 376 | T294 | 0 (0%) | 1 (0%) | 1.000 |
| 377 | T296 | 0 (0%) | 3 (0%) | 1.000 |
| 378 | S297 | 0 (0%) | 6 (0%) | 1.000 |
| 379 | T297 | 0 (0%) | 3 (0%) | 1.000 |
| 380 | S298 | 0 (0%) | 33 (0%) | 0.912 |
| 381 | S299 | 4 (0%) | 222 (0%) | 1.000 |
| 382 | S300 | 81 (0.6%) | 15623 (2%) | <.001 |
| 383 | T300 | 15 (0.1%) | 441 (0.1%) | 0.022 |
| 384 | S301 | 114 (0.8%) | 9878 (1.3%) | <.001 |
| 385 | T301 | 0 (0%) | 18 (0%) | 1.000 |
| 386 | S302 | 6 (0%) | 860 (0.1%) | 0.023 |
| 387 | T302 | 4 (0%) | 369 (0%) | 0.410 |
| 388 | T303 | 6 (0%) | 24 (0%) | <.001 |
| 389 | T304 | 1 (0%) | 29 (0%) | 1.000 |
| 390 | S307 | 1 (0%) | 63 (0%) | 1.000 |
| 391 | S308 | 12 (0.1%) | 916 (0.1%) | 0.330 |
| 392 | S309 | 2 (0%) | 201 (0%) | 0.562 |
| 393 | S310 | 18 (0.1%) | 705 (0.1%) | 0.183 |
| 394 | T310 | 9 (0.1%) | 1076 (0.1%) | 0.026 |
| 395 | S311 | 31 (0.2%) | 1380 (0.2%) | 0.262 |
| 396 | T311 | 8 (0.1%) | 375 (0%) | 0.778 |
| 397 | S312 | 0 (0%) | 147 (0%) | 0.189 |
| 398 | T312 | 10 (0.1%) | 190 (0%) | 0.001 |
| 399 | S313 | 5 (0%) | 353 (0%) | 0.739 |
| 400 | T313 | 16 (0.1%) | 71 (0%) | <.001 |
| 401 | S314 | 1 (0%) | 330 (0%) | 0.069 |
| 402 | T314 | 16 (0.1%) | 30 (0%) | <.001 |
| 403 | S315 | 0 (0%) | 23 (0%) | 1.000 |
| 404 | T315 | 8 (0.1%) | 17 (0%) | <.001 |
| 405 | T316 | 19 (0.1%) | 18 (0%) | <.001 |
| 406 | S317 | 4 (0%) | 60 (0%) | 0.025 |
| 407 | T317 | 8 (0.1%) | 5 (0%) | <.001 |
| 408 | S318 | 11 (0.1%) | 629 (0.1%) | 1.000 |
| 409 | T318 | 20 (0.1%) | 1 (0%) | <.001 |
| 410 | T319 | 23 (0.2%) | 2 (0%) | <.001 |
| 411 | S320 | 651 (4.7%) | 46209 (6%) | <.001 |
| 412 | T320 | 1 (0%) | 7 (0%) | 0.336 |
| 413 | S321 | 210 (1.5%) | 5477 (0.7%) | <.001 |
| 414 | T321 | 0 (0%) | 1 (0%) | 1.000 |
| 415 | S322 | 9 (0.1%) | 1459 (0.2%) | 0.001 |
| 416 | S323 | 145 (1.1%) | 2392 (0.3%) | <.001 |
| 417 | S324 | 172 (1.2%) | 4351 (0.6%) | <.001 |
| 418 | S325 | 320 (2.3%) | 6834 (0.9%) | <.001 |
| 419 | S326 | 0 (0%) | 1 (0%) | 1.000 |
| 420 | S327 | 122 (0.9%) | 2073 (0.3%) | <.001 |
| 421 | S328 | 649 (4.7%) | 6052 (0.8%) | <.001 |
| 422 | S330 | 1 (0%) | 910 (0.1%) | <.001 |
| 423 | S331 | 8 (0.1%) | 377 (0%) | 0.789 |
| 424 | S332 | 10 (0.1%) | 175 (0%) | <.001 |
| 425 | S333 | 3 (0%) | 63 (0%) | 0.213 |
| 426 | S334 | 8 (0.1%) | 123 (0%) | <.001 |
| 427 | S335 | 90 (0.7%) | 90393 (11.8%) | <.001 |
| 428 | S336 | 5 (0%) | 296 (0%) | 1.000 |
| 429 | S337 | 4 (0%) | 5099 (0.7%) | <.001 |
| 430 | S340 | 1 (0%) | 88 (0%) | 0.953 |
| 431 | S341 | 9 (0.1%) | 303 (0%) | 0.200 |
| 432 | S342 | 1 (0%) | 93 (0%) | 0.899 |
| 433 | S343 | 1 (0%) | 38 (0%) | 1.000 |
| 434 | S344 | 0 (0%) | 28 (0%) | 1.000 |
| 435 | S345 | 3 (0%) | 12 (0%) | <.001 |
| 436 | S346 | 0 (0%) | 3 (0%) | 1.000 |
| 437 | S348 | 1 (0%) | 7 (0%) | 0.336 |
| 438 | S350 | 29 (0.2%) | 58 (0%) | <.001 |
| 439 | S351 | 46 (0.3%) | 64 (0%) | <.001 |
| 440 | S352 | 100 (0.7%) | 474 (0.1%) | <.001 |
| 441 | S353 | 47 (0.3%) | 127 (0%) | <.001 |
| 442 | S354 | 35 (0.3%) | 117 (0%) | <.001 |
| 443 | S355 | 97 (0.7%) | 168 (0%) | <.001 |
| 444 | S357 | 14 (0.1%) | 21 (0%) | <.001 |
| 445 | S358 | 23 (0.2%) | 206 (0%) | <.001 |
| 446 | S359 | 25 (0.2%) | 150 (0%) | <.001 |
| 447 | S360 | 296 (2.2%) | 2913 (0.4%) | <.001 |
| 448 | S361 | 588 (4.3%) | 5360 (0.7%) | <.001 |
| 449 | S362 | 100 (0.7%) | 614 (0.1%) | <.001 |
| 450 | S363 | 32 (0.2%) | 210 (0%) | <.001 |
| 451 | S364 | 195 (1.4%) | 1541 (0.2%) | <.001 |
| 452 | S365 | 121 (0.9%) | 657 (0.1%) | <.001 |
| 453 | S366 | 20 (0.1%) | 169 (0%) | <.001 |
| 454 | S367 | 48 (0.3%) | 91 (0%) | <.001 |
| 455 | S368 | 604 (4.4%) | 3553 (0.5%) | <.001 |
| 456 | S369 | 34 (0.2%) | 290 (0%) | <.001 |
| 457 | S370 | 163 (1.2%) | 2280 (0.3%) | <.001 |
| 458 | S371 | 7 (0.1%) | 58 (0%) | <.001 |
| 459 | S372 | 67 (0.5%) | 404 (0.1%) | <.001 |
| 460 | S373 | 35 (0.3%) | 834 (0.1%) | <.001 |
| 461 | S374 | 1 (0%) | 5 (0%) | 0.222 |
| 462 | S375 | 0 (0%) | 1 (0%) | 1.000 |
| 463 | S376 | 0 (0%) | 12 (0%) | 1.000 |
| 464 | S377 | 6 (0%) | 10 (0%) | <.001 |
| 465 | S378 | 42 (0.3%) | 407 (0.1%) | <.001 |
| 466 | S379 | 7 (0.1%) | 44 (0%) | <.001 |
| 467 | S380 | 0 (0%) | 10 (0%) | 1.000 |
| 468 | S381 | 6 (0%) | 49 (0%) | <.001 |
| 469 | S382 | 0 (0%) | 32 (0%) | 0.930 |
| 470 | S383 | 0 (0%) | 1 (0%) | 1.000 |
| 471 | S390 | 12 (0.1%) | 293 (0%) | 0.008 |
| 472 | S396 | 5 (0%) | 14 (0%) | <.001 |
| 473 | S397 | 2 (0%) | 13 (0%) | 0.016 |
| 474 | S398 | 6 (0%) | 217 (0%) | 0.429 |
| 475 | S399 | 17 (0.1%) | 899 (0.1%) | 0.940 |
| 476 | S400 | 37 (0.3%) | 19280 (2.5%) | <.001 |
| 477 | S407 | 1 (0%) | 57 (0%) | 1.000 |
| 478 | S408 | 1 (0%) | 594 (0.1%) | 0.005 |
| 479 | S409 | 0 (0%) | 92 (0%) | 0.373 |
| 480 | S410 | 2 (0%) | 280 (0%) | 0.261 |
| 481 | S411 | 2 (0%) | 668 (0.1%) | 0.006 |
| 482 | S417 | 0 (0%) | 109 (0%) | 0.299 |
| 483 | S418 | 3 (0%) | 64 (0%) | 0.223 |
| 484 | S420 | 348 (2.5%) | 17176 (2.2%) | 0.029 |
| 485 | S421 | 168 (1.2%) | 5181 (0.7%) | <.001 |
| 486 | S422 | 120 (0.9%) | 10743 (1.4%) | <.001 |
| 487 | S423 | 161 (1.2%) | 6336 (0.8%) | <.001 |
| 488 | S424 | 36 (0.3%) | 9636 (1.3%) | <.001 |
| 489 | S427 | 4 (0%) | 68 (0%) | 0.046 |
| 490 | S428 | 0 (0%) | 97 (0%) | 0.349 |
| 491 | S429 | 5 (0%) | 153 (0%) | 0.303 |
| 492 | S430 | 17 (0.1%) | 2253 (0.3%) | <.001 |
| 493 | S431 | 1 (0%) | 1302 (0.2%) | <.001 |
| 494 | S432 | 0 (0%) | 45 (0%) | 0.738 |
| 495 | S433 | 2 (0%) | 187 (0%) | 0.642 |
| 496 | S434 | 4 (0%) | 13814 (1.8%) | <.001 |
| 497 | S435 | 1 (0%) | 1250 (0.2%) | <.001 |
| 498 | S436 | 0 (0%) | 28 (0%) | 1.000 |
| 499 | S437 | 3 (0%) | 2358 (0.3%) | <.001 |
| 500 | S440 | 0 (0%) | 63 (0%) | 0.557 |
| 501 | S441 | 0 (0%) | 45 (0%) | 0.738 |
| 502 | S442 | 0 (0%) | 252 (0%) | 0.059 |
| 503 | S443 | 0 (0%) | 49 (0%) | 0.691 |
| 504 | S444 | 0 (0%) | 25 (0%) | 1.000 |
| 505 | S445 | 0 (0%) | 13 (0%) | 1.000 |
| 506 | S447 | 1 (0%) | 10 (0%) | 0.485 |
| 507 | S448 | 0 (0%) | 25 (0%) | 1.000 |
| 508 | S449 | 0 (0%) | 12 (0%) | 1.000 |
| 509 | S450 | 2 (0%) | 20 (0%) | 0.072 |
| 510 | S451 | 2 (0%) | 138 (0%) | 1.000 |
| 511 | S452 | 2 (0%) | 36 (0%) | 0.308 |
| 512 | S453 | 0 (0%) | 29 (0%) | 0.986 |
| 513 | S457 | 0 (0%) | 7 (0%) | 1.000 |
| 514 | S458 | 1 (0%) | 46 (0%) | 1.000 |
| 515 | S459 | 2 (0%) | 21 (0%) | 0.084 |
| 516 | S460 | 1 (0%) | 2038 (0.3%) | <.001 |
| 517 | S461 | 0 (0%) | 181 (0%) | 0.128 |
| 518 | S462 | 0 (0%) | 243 (0%) | 0.065 |
| 519 | S463 | 0 (0%) | 283 (0%) | 0.042 |
| 520 | S467 | 0 (0%) | 182 (0%) | 0.126 |
| 521 | S468 | 0 (0%) | 513 (0.1%) | 0.004 |
| 522 | S469 | 1 (0%) | 207 (0%) | 0.252 |
| 523 | S480 | 0 (0%) | 6 (0%) | 1.000 |
| 524 | S481 | 3 (0%) | 26 (0%) | 0.005 |
| 525 | S488 | 0 (0%) | 1 (0%) | 1.000 |
| 526 | S489 | 1 (0%) | 18 (0%) | 0.775 |
| 527 | S497 | 0 (0%) | 10 (0%) | 1.000 |
| 528 | S498 | 0 (0%) | 76 (0%) | 0.463 |
| 529 | S499 | 1 (0%) | 165 (0%) | 0.398 |
| 530 | S500 | 20 (0.1%) | 10074 (1.3%) | <.001 |
| 531 | S501 | 7 (0.1%) | 3219 (0.4%) | <.001 |
| 532 | S507 | 3 (0%) | 179 (0%) | 1.000 |
| 533 | S508 | 6 (0%) | 1714 (0.2%) | <.001 |
| 534 | S509 | 2 (0%) | 471 (0.1%) | 0.041 |
| 535 | S510 | 18 (0.1%) | 1995 (0.3%) | 0.004 |
| 536 | S517 | 4 (0%) | 571 (0.1%) | 0.073 |
| 537 | S518 | 7 (0.1%) | 1388 (0.2%) | <.001 |
| 538 | S519 | 12 (0.1%) | 2024 (0.3%) | <.001 |
| 539 | S520 | 52 (0.4%) | 5395 (0.7%) | <.001 |
| 540 | S521 | 16 (0.1%) | 3625 (0.5%) | <.001 |
| 541 | S522 | 17 (0.1%) | 1614 (0.2%) | 0.033 |
| 542 | S523 | 16 (0.1%) | 1563 (0.2%) | 0.029 |
| 543 | S524 | 14 (0.1%) | 2183 (0.3%) | <.001 |
| 544 | S525 | 96 (0.7%) | 21215 (2.8%) | <.001 |
| 545 | S526 | 44 (0.3%) | 9091 (1.2%) | <.001 |
| 546 | S527 | 4 (0%) | 335 (0%) | 0.538 |
| 547 | S528 | 23 (0.2%) | 2267 (0.3%) | 0.007 |
| 548 | S529 | 6 (0%) | 500 (0.1%) | 0.409 |
| 549 | S530 | 1 (0%) | 138 (0%) | 0.537 |
| 550 | S531 | 7 (0.1%) | 1557 (0.2%) | <.001 |
| 551 | S532 | 0 (0%) | 632 (0.1%) | 0.001 |
| 552 | S533 | 1 (0%) | 791 (0.1%) | <.001 |
| 553 | S534 | 3 (0%) | 3069 (0.4%) | <.001 |
| 554 | S540 | 1 (0%) | 446 (0.1%) | 0.022 |
| 555 | S541 | 1 (0%) | 296 (0%) | 0.098 |
| 556 | S542 | 1 (0%) | 515 (0.1%) | 0.011 |
| 557 | S543 | 0 (0%) | 76 (0%) | 0.463 |
| 558 | S547 | 0 (0%) | 33 (0%) | 0.912 |
| 559 | S548 | 0 (0%) | 82 (0%) | 0.426 |
| 560 | S549 | 0 (0%) | 33 (0%) | 0.912 |
| 561 | S550 | 0 (0%) | 237 (0%) | 0.069 |
| 562 | S551 | 5 (0%) | 390 (0.1%) | 0.571 |
| 563 | S552 | 0 (0%) | 167 (0%) | 0.150 |
| 564 | S557 | 0 (0%) | 15 (0%) | 1.000 |
| 565 | S558 | 3 (0%) | 122 (0%) | 0.844 |
| 566 | S559 | 0 (0%) | 35 (0%) | 0.879 |
| 567 | S560 | 0 (0%) | 138 (0%) | 0.210 |
| 568 | S561 | 0 (0%) | 798 (0.1%) | <.001 |
| 569 | S562 | 4 (0%) | 1381 (0.2%) | <.001 |
| 570 | S563 | 0 (0%) | 361 (0%) | 0.019 |
| 571 | S564 | 2 (0%) | 1008 (0.1%) | <.001 |
| 572 | S565 | 0 (0%) | 937 (0.1%) | <.001 |
| 573 | S567 | 1 (0%) | 541 (0.1%) | 0.008 |
| 574 | S568 | 4 (0%) | 803 (0.1%) | 0.009 |
| 575 | S570 | 3 (0%) | 89 (0%) | 0.490 |
| 576 | S578 | 1 (0%) | 138 (0%) | 0.537 |
| 577 | S579 | 1 (0%) | 139 (0%) | 0.531 |
| 578 | S580 | 0 (0%) | 18 (0%) | 1.000 |
| 579 | S581 | 1 (0%) | 32 (0%) | 1.000 |
| 580 | S589 | 2 (0%) | 44 (0%) | 0.442 |
| 581 | S597 | 0 (0%) | 37 (0%) | 0.847 |
| 582 | S598 | 0 (0%) | 97 (0%) | 0.349 |
| 583 | S599 | 2 (0%) | 91 (0%) | 1.000 |
| 584 | S600 | 0 (0%) | 1535 (0.2%) | <.001 |
| 585 | S601 | 0 (0%) | 235 (0%) | 0.070 |
| 586 | S602 | 22 (0.2%) | 8505 (1.1%) | <.001 |
| 587 | S607 | 2 (0%) | 176 (0%) | 0.713 |
| 588 | S608 | 10 (0.1%) | 2322 (0.3%) | <.001 |
| 589 | S609 | 1 (0%) | 418 (0.1%) | 0.028 |
| 590 | S610 | 14 (0.1%) | 12077 (1.6%) | <.001 |
| 591 | S611 | 1 (0%) | 4789 (0.6%) | <.001 |
| 592 | S615 | 0 (0%) | 1 (0%) | 1.000 |
| 593 | S617 | 14 (0.1%) | 1802 (0.2%) | 0.002 |
| 594 | S618 | 29 (0.2%) | 4781 (0.6%) | <.001 |
| 595 | S619 | 28 (0.2%) | 4858 (0.6%) | <.001 |
| 596 | S620 | 4 (0%) | 1107 (0.1%) | <.001 |
| 597 | S621 | 7 (0.1%) | 2067 (0.3%) | <.001 |
| 598 | S622 | 7 (0.1%) | 1050 (0.1%) | 0.009 |
| 599 | S623 | 40 (0.3%) | 5265 (0.7%) | <.001 |
| 600 | S624 | 6 (0%) | 502 (0.1%) | 0.403 |
| 601 | S625 | 2 (0%) | 3114 (0.4%) | <.001 |
| 602 | S626 | 28 (0.2%) | 17920 (2.3%) | <.001 |
| 603 | S627 | 1 (0%) | 701 (0.1%) | 0.002 |
| 604 | S628 | 16 (0.1%) | 919 (0.1%) | 0.993 |
| 605 | S630 | 1 (0%) | 663 (0.1%) | 0.003 |
| 606 | S631 | 6 (0%) | 1057 (0.1%) | 0.004 |
| 607 | S632 | 0 (0%) | 27 (0%) | 1.000 |
| 608 | S633 | 0 (0%) | 583 (0.1%) | 0.002 |
| 609 | S634 | 0 (0%) | 1141 (0.1%) | <.001 |
| 610 | S635 | 2 (0%) | 5071 (0.7%) | <.001 |
| 611 | S636 | 1 (0%) | 1471 (0.2%) | <.001 |
| 612 | S637 | 0 (0%) | 799 (0.1%) | <.001 |
| 613 | S640 | 0 (0%) | 594 (0.1%) | 0.002 |
| 614 | S641 | 0 (0%) | 550 (0.1%) | 0.003 |
| 615 | S642 | 0 (0%) | 584 (0.1%) | 0.002 |
| 616 | S643 | 1 (0%) | 832 (0.1%) | <.001 |
| 617 | S644 | 0 (0%) | 3448 (0.5%) | <.001 |
| 618 | S647 | 0 (0%) | 139 (0%) | 0.208 |
| 619 | S648 | 0 (0%) | 228 (0%) | 0.076 |
| 620 | S649 | 0 (0%) | 119 (0%) | 0.264 |
| 621 | S650 | 0 (0%) | 455 (0.1%) | 0.007 |
| 622 | S651 | 0 (0%) | 620 (0.1%) | 0.001 |
| 623 | S652 | 0 (0%) | 46 (0%) | 0.726 |
| 624 | S653 | 1 (0%) | 26 (0%) | 0.974 |
| 625 | S654 | 1 (0%) | 376 (0%) | 0.043 |
| 626 | S655 | 0 (0%) | 1955 (0.3%) | <.001 |
| 627 | S657 | 0 (0%) | 70 (0%) | 0.503 |
| 628 | S658 | 0 (0%) | 350 (0%) | 0.021 |
| 629 | S659 | 1 (0%) | 116 (0%) | 0.690 |
| 630 | S660 | 1 (0%) | 785 (0.1%) | <.001 |
| 631 | S661 | 4 (0%) | 4306 (0.6%) | <.001 |
| 632 | S662 | 0 (0%) | 2747 (0.4%) | <.001 |
| 633 | S663 | 0 (0%) | 10561 (1.4%) | <.001 |
| 634 | S664 | 0 (0%) | 480 (0.1%) | 0.006 |
| 635 | S665 | 0 (0%) | 555 (0.1%) | 0.003 |
| 636 | S666 | 0 (0%) | 1077 (0.1%) | <.001 |
| 637 | S667 | 1 (0%) | 968 (0.1%) | <.001 |
| 638 | S668 | 0 (0%) | 1889 (0.2%) | <.001 |
| 639 | S669 | 2 (0%) | 683 (0.1%) | 0.005 |
| 640 | S670 | 2 (0%) | 4922 (0.6%) | <.001 |
| 641 | S678 | 4 (0%) | 1004 (0.1%) | 0.001 |
| 642 | S680 | 0 (0%) | 922 (0.1%) | <.001 |
| 643 | S681 | 3 (0%) | 4947 (0.6%) | <.001 |
| 644 | S682 | 0 (0%) | 610 (0.1%) | 0.002 |
| 645 | S683 | 0 (0%) | 159 (0%) | 0.164 |
| 646 | S684 | 0 (0%) | 44 (0%) | 0.750 |
| 647 | S688 | 1 (0%) | 96 (0%) | 0.868 |
| 648 | S689 | 0 (0%) | 61 (0%) | 0.574 |
| 649 | S697 | 1 (0%) | 83 (0%) | 1.000 |
| 650 | S698 | 0 (0%) | 437 (0.1%) | 0.009 |
| 651 | S699 | 2 (0%) | 561 (0.1%) | 0.017 |
| 652 | S700 | 80 (0.6%) | 10603 (1.4%) | <.001 |
| 653 | S701 | 19 (0.1%) | 5546 (0.7%) | <.001 |
| 654 | S707 | 2 (0%) | 59 (0%) | 0.682 |
| 655 | S708 | 6 (0%) | 664 (0.1%) | 0.117 |
| 656 | S709 | 2 (0%) | 198 (0%) | 0.578 |
| 657 | S710 | 2 (0%) | 227 (0%) | 0.437 |
| 658 | S711 | 24 (0.2%) | 1644 (0.2%) | 0.353 |
| 659 | S717 | 3 (0%) | 111 (0%) | 0.731 |
| 660 | S718 | 0 (0%) | 18 (0%) | 1.000 |
| 661 | S720 | 827 (6%) | 27396 (3.6%) | <.001 |
| 662 | S721 | 1205 (8.8%) | 33509 (4.4%) | <.001 |
| 663 | S722 | 98 (0.7%) | 2716 (0.4%) | <.001 |
| 664 | S723 | 201 (1.5%) | 7236 (0.9%) | <.001 |
| 665 | S724 | 99 (0.7%) | 5015 (0.7%) | 0.391 |
| 666 | S727 | 12 (0.1%) | 117 (0%) | <.001 |
| 667 | S728 | 43 (0.3%) | 1027 (0.1%) | <.001 |
| 668 | S729 | 214 (1.6%) | 3604 (0.5%) | <.001 |
| 669 | S730 | 33 (0.2%) | 1688 (0.2%) | 0.705 |
| 670 | S731 | 8 (0.1%) | 2805 (0.4%) | <.001 |
| 671 | S740 | 1 (0%) | 96 (0%) | 0.868 |
| 672 | S741 | 0 (0%) | 31 (0%) | 0.948 |
| 673 | S742 | 0 (0%) | 18 (0%) | 1.000 |
| 674 | S747 | 0 (0%) | 2 (0%) | 1.000 |
| 675 | S748 | 0 (0%) | 16 (0%) | 1.000 |
| 676 | S749 | 0 (0%) | 8 (0%) | 1.000 |
| 677 | S750 | 26 (0.2%) | 153 (0%) | <.001 |
| 678 | S751 | 5 (0%) | 56 (0%) | <.001 |
| 679 | S752 | 0 (0%) | 14 (0%) | 1.000 |
| 680 | S757 | 0 (0%) | 9 (0%) | 1.000 |
| 681 | S758 | 4 (0%) | 96 (0%) | 0.189 |
| 682 | S759 | 4 (0%) | 99 (0%) | 0.209 |
| 683 | S760 | 2 (0%) | 329 (0%) | 0.162 |
| 684 | S761 | 1 (0%) | 1860 (0.2%) | <.001 |
| 685 | S762 | 2 (0%) | 257 (0%) | 0.327 |
| 686 | S763 | 1 (0%) | 335 (0%) | 0.066 |
| 687 | S764 | 2 (0%) | 1063 (0.1%) | <.001 |
| 688 | S767 | 0 (0%) | 303 (0%) | 0.034 |
| 689 | S770 | 0 (0%) | 11 (0%) | 1.000 |
| 690 | S771 | 4 (0%) | 84 (0%) | 0.116 |
| 691 | S772 | 1 (0%) | 21 (0%) | 0.858 |
| 692 | S780 | 0 (0%) | 1 (0%) | 1.000 |
| 693 | S781 | 2 (0%) | 18 (0%) | 0.052 |
| 694 | S789 | 0 (0%) | 4 (0%) | 1.000 |
| 695 | T790 | 0 (0%) | 1 (0%) | 1.000 |
| 696 | T791 | 15 (0.1%) | 29 (0%) | <.001 |
| 697 | T792 | 4 (0%) | 9 (0%) | <.001 |
| 698 | T793 | 8 (0.1%) | 466 (0.1%) | 1.000 |
| 699 | T794 | 377 (2.7%) | 412 (0.1%) | <.001 |
| 700 | T795 | 4 (0%) | 1 (0%) | <.001 |
| 701 | T796 | 106 (0.8%) | 1250 (0.2%) | <.001 |
| 702 | S797 | 0 (0%) | 14 (0%) | 1.000 |
| 703 | T797 | 40 (0.3%) | 883 (0.1%) | <.001 |
| 704 | S798 | 0 (0%) | 40 (0%) | 0.804 |
| 705 | T798 | 2 (0%) | 87 (0%) | 1.000 |
| 706 | S799 | 3 (0%) | 93 (0%) | 0.534 |
| 707 | T799 | 1 (0%) | 4 (0%) | 0.163 |
| 708 | S800 | 45 (0.3%) | 27029 (3.5%) | <.001 |
| 709 | S801 | 16 (0.1%) | 8759 (1.1%) | <.001 |
| 710 | S807 | 5 (0%) | 363 (0%) | 0.690 |
| 711 | S808 | 21 (0.2%) | 3246 (0.4%) | <.001 |
| 712 | S809 | 2 (0%) | 572 (0.1%) | 0.015 |
| 713 | S810 | 30 (0.2%) | 5080 (0.7%) | <.001 |
| 714 | S817 | 9 (0.1%) | 680 (0.1%) | 0.438 |
| 715 | S818 | 21 (0.2%) | 2333 (0.3%) | 0.002 |
| 716 | S819 | 24 (0.2%) | 2869 (0.4%) | <.001 |
| 717 | S820 | 55 (0.4%) | 9888 (1.3%) | <.001 |
| 718 | S821 | 118 (0.9%) | 8572 (1.1%) | 0.004 |
| 719 | S822 | 252 (1.8%) | 10505 (1.4%) | <.001 |
| 720 | S823 | 100 (0.7%) | 10213 (1.3%) | <.001 |
| 721 | S824 | 71 (0.5%) | 5882 (0.8%) | <.001 |
| 722 | S825 | 51 (0.4%) | 4858 (0.6%) | <.001 |
| 723 | S826 | 33 (0.2%) | 10310 (1.3%) | <.001 |
| 724 | S827 | 8 (0.1%) | 247 (0%) | 0.155 |
| 725 | S828 | 79 (0.6%) | 13118 (1.7%) | <.001 |
| 726 | S829 | 12 (0.1%) | 348 (0%) | 0.040 |
| 727 | S830 | 0 (0%) | 414 (0.1%) | 0.011 |
| 728 | S831 | 3 (0%) | 226 (0%) | 0.783 |
| 729 | S832 | 0 (0%) | 2880 (0.4%) | <.001 |
| 730 | S833 | 0 (0%) | 150 (0%) | 0.182 |
| 731 | S834 | 7 (0.1%) | 4441 (0.6%) | <.001 |
| 732 | S835 | 6 (0%) | 4070 (0.5%) | <.001 |
| 733 | S836 | 7 (0.1%) | 9503 (1.2%) | <.001 |
| 734 | S837 | 0 (0%) | 407 (0.1%) | 0.012 |
| 735 | S840 | 0 (0%) | 133 (0%) | 0.223 |
| 736 | S841 | 0 (0%) | 469 (0.1%) | 0.006 |
| 737 | S842 | 0 (0%) | 45 (0%) | 0.738 |
| 738 | S847 | 0 (0%) | 11 (0%) | 1.000 |
| 739 | S848 | 0 (0%) | 85 (0%) | 0.409 |
| 740 | S849 | 0 (0%) | 21 (0%) | 1.000 |
| 741 | S850 | 9 (0.1%) | 103 (0%) | <.001 |
| 742 | S851 | 8 (0.1%) | 216 (0%) | 0.073 |
| 743 | S852 | 1 (0%) | 22 (0%) | 0.883 |
| 744 | S853 | 0 (0%) | 46 (0%) | 0.726 |
| 745 | S854 | 0 (0%) | 18 (0%) | 1.000 |
| 746 | S855 | 3 (0%) | 22 (0%) | 0.002 |
| 747 | S857 | 0 (0%) | 14 (0%) | 1.000 |
| 748 | S858 | 2 (0%) | 166 (0%) | 0.783 |
| 749 | S859 | 1 (0%) | 99 (0%) | 0.839 |
| 750 | S860 | 2 (0%) | 4024 (0.5%) | <.001 |
| 751 | S861 | 2 (0%) | 927 (0.1%) | <.001 |
| 752 | S862 | 0 (0%) | 855 (0.1%) | <.001 |
| 753 | S863 | 0 (0%) | 350 (0%) | 0.021 |
| 754 | S867 | 5 (0%) | 447 (0.1%) | 0.373 |
| 755 | S868 | 3 (0%) | 1236 (0.2%) | <.001 |
| 756 | S869 | 1 (0%) | 619 (0.1%) | 0.004 |
| 757 | S870 | 2 (0%) | 197 (0%) | 0.584 |
| 758 | S878 | 18 (0.1%) | 382 (0%) | <.001 |
| 759 | S880 | 4 (0%) | 19 (0%) | <.001 |
| 760 | S881 | 0 (0%) | 42 (0%) | 0.776 |
| 761 | S884 | 0 (0%) | 1 (0%) | 1.000 |
| 762 | S889 | 2 (0%) | 20 (0%) | 0.072 |
| 763 | S897 | 1 (0%) | 17 (0%) | 0.745 |
| 764 | S898 | 1 (0%) | 207 (0%) | 0.252 |
| 765 | S899 | 3 (0%) | 207 (0%) | 0.911 |
| 766 | S900 | 8 (0.1%) | 4321 (0.6%) | <.001 |
| 767 | T900 | 0 (0%) | 2 (0%) | 1.000 |
| 768 | S901 | 1 (0%) | 630 (0.1%) | 0.004 |
| 769 | T901 | 0 (0%) | 3 (0%) | 1.000 |
| 770 | S902 | 0 (0%) | 167 (0%) | 0.150 |
| 771 | T902 | 1 (0%) | 25 (0%) | 0.952 |
| 772 | S903 | 7 (0.1%) | 4377 (0.6%) | <.001 |
| 773 | T904 | 0 (0%) | 5 (0%) | 1.000 |
| 774 | T905 | 3 (0%) | 98 (0%) | 0.590 |
| 775 | S907 | 1 (0%) | 144 (0%) | 0.502 |
| 776 | S908 | 8 (0.1%) | 1186 (0.2%) | 0.006 |
| 777 | T908 | 0 (0%) | 4 (0%) | 1.000 |
| 778 | S909 | 0 (0%) | 202 (0%) | 0.101 |
| 779 | T909 | 1 (0%) | 13 (0%) | 0.609 |
| 780 | S910 | 12 (0.1%) | 1633 (0.2%) | 0.002 |
| 781 | T910 | 0 (0%) | 2 (0%) | 1.000 |
| 782 | S911 | 4 (0%) | 1023 (0.1%) | 0.001 |
| 783 | T911 | 4 (0%) | 166 (0%) | 0.774 |
| 784 | S912 | 1 (0%) | 573 (0.1%) | 0.006 |
| 785 | T912 | 2 (0%) | 32 (0%) | 0.242 |
| 786 | S913 | 16 (0.1%) | 3616 (0.5%) | <.001 |
| 787 | T913 | 1 (0%) | 50 (0%) | 1.000 |
| 788 | T914 | 0 (0%) | 1 (0%) | 1.000 |
| 789 | T915 | 0 (0%) | 5 (0%) | 1.000 |
| 790 | S917 | 2 (0%) | 368 (0%) | 0.111 |
| 791 | T918 | 0 (0%) | 2 (0%) | 1.000 |
| 792 | S919 | 0 (0%) | 1 (0%) | 1.000 |
| 793 | T919 | 0 (0%) | 1 (0%) | 1.000 |
| 794 | S920 | 51 (0.4%) | 9556 (1.3%) | <.001 |
| 795 | T920 | 0 (0%) | 4 (0%) | 1.000 |
| 796 | S921 | 12 (0.1%) | 1818 (0.2%) | <.001 |
| 797 | T921 | 0 (0%) | 16 (0%) | 1.000 |
| 798 | S922 | 18 (0.1%) | 4153 (0.5%) | <.001 |
| 799 | T922 | 0 (0%) | 8 (0%) | 1.000 |
| 800 | S923 | 47 (0.3%) | 10699 (1.4%) | <.001 |
| 801 | T923 | 0 (0%) | 3 (0%) | 1.000 |
| 802 | S924 | 7 (0.1%) | 3436 (0.4%) | <.001 |
| 803 | T924 | 0 (0%) | 2 (0%) | 1.000 |
| 804 | S925 | 7 (0.1%) | 3910 (0.5%) | <.001 |
| 805 | T926 | 0 (0%) | 12 (0%) | 1.000 |
| 806 | S927 | 4 (0%) | 607 (0.1%) | 0.053 |
| 807 | T928 | 0 (0%) | 1 (0%) | 1.000 |
| 808 | S929 | 7 (0.1%) | 520 (0.1%) | 0.547 |
| 809 | S930 | 2 (0%) | 1240 (0.2%) | <.001 |
| 810 | T930 | 0 (0%) | 7 (0%) | 1.000 |
| 811 | S931 | 1 (0%) | 506 (0.1%) | 0.012 |
| 812 | T931 | 1 (0%) | 19 (0%) | 0.804 |
| 813 | S932 | 2 (0%) | 3391 (0.4%) | <.001 |
| 814 | T932 | 0 (0%) | 18 (0%) | 1.000 |
| 815 | S933 | 1 (0%) | 859 (0.1%) | <.001 |
| 816 | T933 | 0 (0%) | 6 (0%) | 1.000 |
| 817 | S934 | 6 (0%) | 11247 (1.5%) | <.001 |
| 818 | T934 | 0 (0%) | 1 (0%) | 1.000 |
| 819 | S935 | 0 (0%) | 219 (0%) | 0.084 |
| 820 | T935 | 0 (0%) | 3 (0%) | 1.000 |
| 821 | S936 | 1 (0%) | 1523 (0.2%) | <.001 |
| 822 | T936 | 0 (0%) | 4 (0%) | 1.000 |
| 823 | T938 | 0 (0%) | 1 (0%) | 1.000 |
| 824 | T939 | 0 (0%) | 2 (0%) | 1.000 |
| 825 | S940 | 0 (0%) | 6 (0%) | 1.000 |
| 826 | S941 | 0 (0%) | 27 (0%) | 1.000 |
| 827 | T941 | 0 (0%) | 1 (0%) | 1.000 |
| 828 | S942 | 0 (0%) | 121 (0%) | 0.258 |
| 829 | S943 | 0 (0%) | 74 (0%) | 0.476 |
| 830 | S947 | 0 (0%) | 14 (0%) | 1.000 |
| 831 | S948 | 0 (0%) | 167 (0%) | 0.150 |
| 832 | S949 | 1 (0%) | 58 (0%) | 1.000 |
| 833 | S950 | 1 (0%) | 112 (0%) | 0.722 |
| 834 | T950 | 0 (0%) | 3 (0%) | 1.000 |
| 835 | S951 | 1 (0%) | 27 (0%) | 0.994 |
| 836 | T951 | 0 (0%) | 1 (0%) | 1.000 |
| 837 | S952 | 0 (0%) | 21 (0%) | 1.000 |
| 838 | T952 | 0 (0%) | 2 (0%) | 1.000 |
| 839 | T953 | 0 (0%) | 4 (0%) | 1.000 |
| 840 | S957 | 0 (0%) | 9 (0%) | 1.000 |
| 841 | S958 | 0 (0%) | 136 (0%) | 0.215 |
| 842 | T958 | 0 (0%) | 5 (0%) | 1.000 |
| 843 | S959 | 0 (0%) | 56 (0%) | 0.619 |
| 844 | S960 | 0 (0%) | 419 (0.1%) | 0.010 |
| 845 | S961 | 1 (0%) | 1881 (0.2%) | <.001 |
| 846 | S962 | 0 (0%) | 250 (0%) | 0.060 |
| 847 | S967 | 2 (0%) | 203 (0%) | 0.551 |
| 848 | S968 | 3 (0%) | 734 (0.1%) | 0.008 |
| 849 | S969 | 1 (0%) | 363 (0%) | 0.050 |
| 850 | S970 | 3 (0%) | 138 (0%) | 0.997 |
| 851 | S971 | 1 (0%) | 326 (0%) | 0.072 |
| 852 | S978 | 4 (0%) | 847 (0.1%) | 0.006 |
| 853 | S980 | 1 (0%) | 22 (0%) | 0.883 |
| 854 | T980 | 0 (0%) | 5 (0%) | 1.000 |
| 855 | S981 | 0 (0%) | 132 (0%) | 0.226 |
| 856 | T981 | 0 (0%) | 2 (0%) | 1.000 |
| 857 | S982 | 1 (0%) | 55 (0%) | 1.000 |
| 858 | T982 | 0 (0%) | 1 (0%) | 1.000 |
| 859 | S983 | 1 (0%) | 27 (0%) | 0.994 |
| 860 | T983 | 0 (0%) | 1 (0%) | 1.000 |
| 861 | S984 | 0 (0%) | 15 (0%) | 1.000 |
| 862 | S996 | 0 (0%) | 1 (0%) | 1.000 |
| 863 | S997 | 0 (0%) | 26 (0%) | 1.000 |
| 864 | S998 | 0 (0%) | 386 (0.1%) | 0.015 |
| 865 | S999 | 0 (0%) | 220 (0%) | 0.083 |
| ICD-10, international classification of disease 10th edition | | | | |

| **Table S4. Ranked normalized feature importance values from AdaBoost** | | |
| --- | --- | --- |
| **Rank** | **Name** | **Value** |
| 1 | Gender | 1 |
| 2 | Age | 0.916 |
| 3 | Unresponsive (coma) | 0.308 |
| 4 | S721 | 0.275 |
| 5 | S720 | 0.25 |
| 6 | Painful response (stupor, semi coma) | 0.25 |
| 7 | Injury mechanism - slip down | 0.25 |
| 8 | Torso procedure-chest | 0.183 |
| 9 | S224 | 0.166 |
| 10 | Head surgery | 0.15 |
| 11 | Verbal response(drowsy) | 0.15 |
| 12 | S335 | 0.15 |
| 13 | S066 | 0.15 |
| 14 | Initial KTAS level 2 | 0.15 |
| 15 | Injury mechanism - firearm/cut (sharp objects)/pierce | 0.15 |
| 16 | S060 | 0.15 |
| 17 | Initial KTAS level 4 | 0.15 |
| 18 | S134 | 0.125 |
| 19 | S065 | 0.125 |
| 20 | Torso procedure-vascular | 0.125 |
| 21 | S525 | 0.125 |
| 22 | S328 | 0.125 |
| 23 | Torso procedure-abdomen | 0.125 |
| 24 | S141 | 0.108 |
| 25 | Initial KTAS level 1 | 0.108 |
| 26 | Altered KTAS level 2 | 0.108 |
| 27 | Altered KTAS level 1 | 0.108 |
| 28 | S062 | 0.108 |
| 29 | S729 | 0.083333333 |
| 30 | Injury mechanism - machine | 0.083333333 |
| 31 | T009 | 0.083333333 |
| 32 | Injury mechanism – Fall down | 0.083333333 |
| 33 | S010 | 0.083333333 |
| 34 | Injury mechanism - TRAFFIC OTHERS | 0.083333333 |
| 35 | S020 | 0.083333333 |
| 36 | Initial KTAS level 5 | 0.083333333 |
| 37 | S320 | 0.083333333 |
| 38 | S223 | 0.083333333 |
| 39 | S368 | 0.083333333 |
| 40 | ECMO | 0.083333333 |
| 41 | S420 | 0.083333333 |
| 42 | S828 | 0.058333333 |
| 43 | S021 | 0.058333333 |
| 44 | S024 | 0.058333333 |
| 45 | S361 | 0.058333333 |
| 46 | Injury mechanism - fire, flames or heat | 0.058333333 |
| 47 | Emergency symptoms | 0.058333333 |
| 48 | S800 | 0.058333333 |
| 49 | S220 | 0.058333333 |
| 50 | S626 | 0.058333333 |
| 51 | S923 | 0.041666667 |
| 52 | S526 | 0.041666667 |
| 53 | S018 | 0.041666667 |
| 54 | S820 | 0.041666667 |
| 55 | S068 | 0.041666667 |
| 56 | S008 | 0.041666667 |
| 57 | T794 | 0.041666667 |
| 58 | Injury mechanism - traffic accident by bike | 0.041666667 |
| 59 | S022 | 0.041666667 |
| 60 | S920 | 0.041666667 |
| 61 | S823 | 0.025 |
| 62 | S063 | 0.025 |
| 63 | S028 | 0.025 |
| 64 | S610 | 0.025 |
| 65 | S826 | 0.025 |
| 66 | S064 | 0.025 |
| 67 | S300 | 0.025 |
| 68 | S822 | 0.025 |
| 69 | S400 | 0.025 |
| 70 | S722 | 0.025 |
| 71 | Injury mechanism - struck by person or object | 0.025 |
| KTAS, Korean triage and acuity scale; ECMO, extracorporeal membrane oxygenation | | |

| **Table S5. Cross-validation result comparison of DNNs using a different set of features** | | | | | |
| --- | --- | --- | --- | --- | --- |
| **Model** | **Sensitivity** | **Specificity** | **Accuracy** | **Balanced accuracy** | **AUROC** |
| **DNN with all 914 features** | **0.8599 ± 0.0151** | **0.8838 ± 0.0097** | **0.8834 ± 0.0093** | **0.8718 ± 0.0036** | **0.9513 ± 0.0023** |
| DNN with top 71 features from AdaBoost | 0.7480 ± 0.0220 | 0.9299 ± 0.0075 | 0.9265 ± 0.0074 | 0.8389 ± 0.0106 | 0.9386 ± 0.0143 |
| DNN, deep neural network; AUROC, area under the receiver operating characteristic curve | | | | | |

| **Table S6. Survival risk ratio of ICD-10 for calculation of ICISS** | | | | |
| --- | --- | --- | --- | --- |
| **Inclusive SRR** | | | | |
| **Code** | **Name** | **Total**  **(N)** | **Survivor**  **(N)** | **Inclusive**  **SRR (%)** |
| S00 | Superficial injury of head | 34947 | 34607 | 99.03 |
| S001 | Contusion of eyelid and periocular area | 1888 | 1878 | 99.47 |
| S002 | Other superficial injuries of eyelid and periocular area | 587 | 585 | 99.66 |
| S003 | Superficial injury of nose | 913 | 910 | 99.67 |
| S004 | Superficial injury of ear | 321 | 318 | 99.07 |
| S005 | Superficial injury of lip and oral cavity | 801 | 798 | 99.63 |
| S007 | Multiple superficial injuries of head | 434 | 430 | 99.08 |
| S008 | Superficial injury of other parts of head | 17038 | 16890 | 99.13 |
| S009 | Superficial injury of head, part unspecified | 3409 | 3355 | 98.42 |
| S01 | Open wound of head | 47300 | 46514 | 98.34 |
| S010 | Open wound of scalp | 15151 | 14791 | 97.62 |
| S011 | Open wound of eyelid and periocular area | 6339 | 6293 | 99.27 |
| S012 | Open wound of nose | 1651 | 1640 | 99.33 |
| S013 | Open wound of ear | 1575 | 1546 | 98.16 |
| S014 | Open wound of cheek and temporomandibular area | 2774 | 2748 | 99.06 |
| S015 | Open wound of lip | 5547 | 5510 | 99.33 |
| S017 | Multiple open wounds of head | 1057 | 1040 | 98.39 |
| S018 | Open wound of other parts of head | 11879 | 11645 | 98.03 |
| S019 | Open wound of head, part unspecified | 1222 | 1196 | 97.87 |
| S02 | Fracture of skull and facial bones | 73636 | 69453 | 94.32 |
| S020 | Fracture of vault of skull | 8744 | 7598 | 86.89 |
| S021 | Fracture of base of skull | 9912 | 8862 | 89.41 |
| S022 | Fracture of nasal bones | 11628 | 11424 | 98.25 |
| S023 | Fracture of orbital floor | 7188 | 7040 | 97.94 |
| S024 | Fracture of malar and maxillary bones | 12118 | 11680 | 96.39 |
| S025 | Fracture of tooth | 3209 | 3191 | 99.44 |
| S026 | Fracture of mandible | 7191 | 7060 | 98.18 |
| S027 | Multiple fractures involving skull and facial bones | 1025 | 905 | 88.29 |
| S028 | Fractures of other skull and facial bones | 7831 | 7533 | 96.19 |
| S029 | Fracture of skull and facial bones, part unspecified | 4711 | 4084 | 86.69 |
| S03 | Dislocation, sprain and strain of joints and ligaments of head | 2894 | 2875 | 99.34 |
| S030 | Dislocation of jaw | 46 | 43 | 93.48 |
| S031 | Dislocation of septal cartilage of nose | 46 | 46 | 100.00 |
| S032 | Dislocation of tooth | 2373 | 2357 | 99.33 |
| S033 | Dislocation of other and unspecified parts of head | 1 | 1 | 100.00 |
| S034 | Sprain and strain of jaw | 392 | 392 | 100.00 |
| S035 | Sprain and strain of joints and ligaments of other and unspecified parts of head | 34 | 34 | 100.00 |
| S04 | Injury of cranial nerve | 948 | 935 | 98.63 |
| S040 | Injury of optic nerve and pathways | 304 | 300 | 98.68 |
| S041 | Injury of oculomotor nerve | 66 | 63 | 95.45 |
| S042 | Injury of trochlear nerve | 40 | 39 | 97.50 |
| S043 | Injury of trigeminal nerve | 53 | 53 | 100.00 |
| S044 | Injury of abducent nerve | 19 | 19 | 100.00 |
| S045 | Injury of facial nerve | 376 | 375 | 99.73 |
| S046 | Injury of acoustic nerve | 23 | 23 | 100.00 |
| S047 | Injury of accessory nerve | 0 | 0 | 0.00 |
| S048 | Injury of other cranial nerves | 28 | 28 | 100.00 |
| S049 | Injury of unspecified cranial nerve | 33 | 29 | 87.88 |
| S05 | Injury of eye and orbit | 9548 | 9500 | 99.50 |
| S050 | Injury of conjunctiva and corneal abrasion without mention of foreign body | 988 | 980 | 99.19 |
| S051 | Contusion of eyeball and orbital tissues | 3270 | 3251 | 99.42 |
| S052 | Ocular laceration and rupture with prolapse or loss of intraocular tissue | 679 | 675 | 99.41 |
| S053 | Ocular laceration without prolapse or loss of intraocular tissue | 1548 | 1542 | 99.61 |
| S054 | Penetrating wound of orbit with or without foreign body | 123 | 123 | 100.00 |
| S055 | Penetrating wound of eyeball with foreign body | 634 | 634 | 100.00 |
| S056 | Penetrating wound of eyeball without foreign body | 370 | 367 | 99.19 |
| S057 | Avulsion of eye | 12 | 12 | 100.00 |
| S058 | Other injuries of eye and orbit | 1656 | 1652 | 99.76 |
| S059 | Injury of eye and orbit, unspecified | 258 | 254 | 98.45 |
| S06 | Intracranial injury | 178759 | 165144 | 92.38 |
| S060 | Concussion | 87698 | 87409 | 99.67 |
| S061 | Traumatic cerebral oedema | 541 | 243 | 44.92 |
| S062 | Diffuse brain injury | 9016 | 7664 | 85.00 |
| S063 | Focal brain injury | 7013 | 6383 | 91.02 |
| S064 | Epidural haemorrhage | 9345 | 8588 | 91.90 |
| S065 | Traumatic subdural haemorrhage | 39994 | 33992 | 84.99 |
| S066 | Traumatic subarachnoid haemorrhage | 18531 | 15391 | 83.06 |
| S067 | Intracranial injury with prolonged coma | 63 | 15 | 23.81 |
| S068 | Other intracranial injuries | 5943 | 4973 | 83.68 |
| S069 | Intracranial injury, unspecified | 492 | 374 | 76.02 |
| S07 | Crushing injury of head | 189 | 180 | 95.24 |
| S070 | Crushing injury of face | 135 | 130 | 96.30 |
| S071 | Crushing injury of skull | 17 | 15 | 88.24 |
| S078 | Crushing injury of other parts of head | 32 | 30 | 93.75 |
| S079 | Crushing injury of head, part unspecified | 4 | 4 | 100.00 |
| S08 | Traumatic amputation of part of head | 249 | 242 | 97.19 |
| S080 | Avulsion of scalp | 169 | 164 | 97.04 |
| S081 | Traumatic amputation of ear | 65 | 63 | 96.92 |
| S088 | Traumatic amputation of other parts of head | 15 | 15 | 100.00 |
| S089 | Traumatic amputation of unspecified part of head | 0 | 0 | 0.00 |
| S09 | Other and unspecified injuries of head | 4396 | 4237 | 96.38 |
| S090 | Injury of blood vessels of head, NEC | 69 | 65 | 94.20 |
| S091 | Injury of muscle and tendon of head | 1143 | 1138 | 99.56 |
| S092 | Traumatic rupture of ear drum | 247 | 244 | 98.79 |
| S097 | Multiple injuries of head | 221 | 191 | 86.43 |
| S098 | Other specified injuries of head | 1104 | 1031 | 93.39 |
| S099 | Unspecified injury of head | 1601 | 1558 | 97.31 |
| S10 | Superficial injury of neck | 1700 | 1670 | 98.24 |
| S100 | Contusion of throat | 82 | 81 | 98.78 |
| S101 | Other and unspecified superficial injuries of throat | 34 | 33 | 97.06 |
| S107 | Multiple superficial injuries of neck | 18 | 18 | 100.00 |
| S108 | Superficial injury of other parts of neck | 649 | 631 | 97.23 |
| S109 | Superficial injury of neck, part unspecified | 917 | 907 | 98.91 |
| S11 | Open wound of neck | 1172 | 1141 | 97.35 |
| S111 | Open wound involving thyroid gland | 18 | 18 | 100.00 |
| S112 | Open wound involving pharynx and cervical esophagus | 29 | 27 | 93.10 |
| S117 | Multiple open wounds of neck | 98 | 95 | 96.94 |
| S118 | Open wound of other parts of neck | 314 | 312 | 99.36 |
| S119 | Open wound of neck, part unspecified | 650 | 629 | 96.77 |
| S12 | Fracture of neck | 8339 | 7791 | 93.43 |
| S120 | Fracture of first cervical vertebra | 491 | 438 | 89.21 |
| S121 | Fracture of second cervical vertebra | 1234 | 1146 | 92.87 |
| S122 | Fracture of other specified cervical vertebra | 2733 | 2609 | 95.46 |
| S127 | Multiple fractures of cervical spine | 1097 | 997 | 90.88 |
| S128 | Fracture of other parts of neck | 159 | 154 | 96.86 |
| S129 | Fracture of neck, part unspecified | 2608 | 2430 | 93.17 |
| S13 | Dislocation, sprain and strain of joints and ligaments at neck level | 108093 | 107806 | 99.73 |
| S130 | Traumatic rupture of cervical intervertebral disc | 802 | 787 | 98.13 |
| S131 | Dislocation of cervical vertebra | 1299 | 1160 | 89.30 |
| S132 | Dislocation of other and unspecified parts of neck | 4 | 4 | 100.00 |
| S133 | Multiple dislocations of neck | 29 | 29 | 100.00 |
| S134 | Sprain and strain of cervical spine | 103629 | 103504 | 99.88 |
| S135 | Sprain and strain of thyroid region | 15 | 15 | 100.00 |
| S136 | Sprain and strain of joints and ligaments of other and unspecified parts of neck | 2294 | 2286 | 99.65 |
| S14 | Injury of nerves and spinal cord at neck level | 4450 | 4221 | 94.85 |
| S140 | Concussion and oedema of cervical spinal cord | 532 | 525 | 98.68 |
| S141 | Other and unspecified injuries of cervical spinal cord | 3291 | 3079 | 93.56 |
| S142 | Injury of nerve root of cervical spine | 188 | 185 | 98.40 |
| S143 | Injury of brachial plexus | 357 | 354 | 99.16 |
| S144 | Injury of peripheral nerves of neck | 4 | 4 | 100.00 |
| S145 | Injury of cervical sympathetic nerves | 5 | 5 | 100.00 |
| S146 | Injury of other and unspecified nerves of neck | 57 | 53 | 92.98 |
| S15 | Injury of blood vessels at neck level | 436 | 386 | 88.53 |
| S150 | Injury of carotid artery | 72 | 54 | 75.00 |
| S151 | Injury of vertebral artery | 63 | 48 | 76.19 |
| S152 | Injury of external jugular vein | 46 | 44 | 95.65 |
| S153 | Injury of internal jugular vein | 42 | 37 | 88.10 |
| S157 | Injury of multiple blood vessels at neck level | 16 | 15 | 93.75 |
| S158 | Injury of other blood vessels at neck level | 117 | 111 | 94.87 |
| S159 | Injury of unspecified blood vessels at neck level | 80 | 77 | 96.25 |
| S16 | Injury of muscle and tendon at neck level | 137 | 137 | 100.00 |
| S17 | Crushing injury of neck | 17 | 17 | 100.00 |
| S170 | Crushing injury of larynx and trachea | 4 | 4 | 100.00 |
| S178 | Crushing injury of other parts of neck | 6 | 6 | 100.00 |
| S179 | Crushing injury of neck, part unspecified | 7 | 7 | 100.00 |
| S18 | Traumatic amputation at neck level | 0 | 0 | 0.00 |
| S19 | Other and unspecified injuries of neck | 355 | 340 | 95.77 |
| S197 | Multiple injuries of neck | 12 | 10 | 83.33 |
| S198 | Other specified injuries of neck | 92 | 87 | 94.57 |
| S199 | Unspecified injury of neck | 251 | 243 | 96.81 |
| S20 | Superficial injury of thorax | 30462 | 30298 | 99.46 |
| S200 | Contusion of breast | 37 | 37 | 100.00 |
| S201 | Other and unspecified superficial injuries of breast | 28 | 28 | 100.00 |
| S202 | Contusion of thorax | 29458 | 29306 | 99.48 |
| S203 | Other superficial injuries of front wall of thorax | 190 | 188 | 98.95 |
| S204 | Other superficial injuries of back wall of thorax | 109 | 107 | 98.17 |
| S207 | Multiple superficial injuries of thorax | 37 | 36 | 97.30 |
| S208 | Superficial injury of other and unspecified parts of thorax | 598 | 591 | 98.83 |
| S21 | Open wound of thorax | 1051 | 1038 | 98.76 |
| S210 | Open wound of breast | 17 | 17 | 100.00 |
| S211 | Open wound of front wall of thorax | 344 | 337 | 97.97 |
| S212 | Open wound of back wall of thorax | 266 | 264 | 99.25 |
| S217 | Multiple open wounds of thoracic wall | 29 | 29 | 100.00 |
| S218 | Open wound of other parts of thorax | 75 | 75 | 100.00 |
| S219 | Open wound of thorax, part unspecified | 315 | 311 | 98.73 |
| S22 | Fracture of rib(s), sternum and thoracic spine | 108601 | 105829 | 97.45 |
| S220 | Fracture of thoracic vertebra | 31594 | 31059 | 98.31 |
| S221 | Multiple fractures of thoracic spine | 1554 | 1482 | 95.37 |
| S222 | Fracture of sternum | 7742 | 7558 | 97.62 |
| S223 | Fracture of rib | 15442 | 15248 | 98.74 |
| S224 | Multiple fracture of ribs | 51334 | 49653 | 96.73 |
| S225 | Flail chest | 606 | 516 | 85.15 |
| S228 | Fracture of other parts of bony thorax | 153 | 144 | 94.12 |
| S229 | Fracture of bony thorax, part unspecified | 61 | 60 | 98.36 |
| S23 | Dislocation, sprain and strain of joints and ligaments of thorax | 11391 | 11373 | 99.84 |
| S230 | Traumatic rupture of thoracic intervertebral disc | 30 | 30 | 100.00 |
| S231 | Dislocation of thoracic vertebra | 107 | 100 | 93.46 |
| S232 | Dislocation of other and unspecified parts of thorax | 21 | 21 | 100.00 |
| S233 | Sprain and strain of thoracic spine | 7150 | 7142 | 99.89 |
| S234 | Sprain and strain of ribs and sternum | 3390 | 3387 | 99.91 |
| S235 | Sprain and strain of other and unspecified parts of thorax | 689 | 689 | 100.00 |
| S24 | Injury of nerves and spinal cord at thorax level | 287 | 270 | 94.08 |
| S240 | Concussion and oedema of thoracic spinal cord | 24 | 24 | 100.00 |
| S241 | Other and unspecified injuries of thoracic spinal cord | 223 | 210 | 94.17 |
| S242 | Injury of nerve root of thoracic spine | 24 | 21 | 87.50 |
| S243 | Injury of peripheral nerves of thorax | 0 | 0 | 0.00 |
| S244 | Injury of thoracic sympathetic nerves | 3 | 3 | 100.00 |
| S245 | Injury of other nerves of thorax | 5 | 4 | 80.00 |
| S246 | Injury of unspecified nerve of thorax | 7 | 7 | 100.00 |
| S25 | Injury of blood vessels of thorax | 573 | 421 | 73.47 |
| S250 | Injury of thoracic aorta | 303 | 212 | 69.97 |
| S251 | Injury of innominate or subclavian artery | 30 | 24 | 80.00 |
| S252 | Injury of superior vena cava | 11 | 4 | 36.36 |
| S253 | Injury of innominate or subclavian vein | 14 | 11 | 78.57 |
| S254 | Injury of pulmonary blood vessels | 36 | 23 | 63.89 |
| S255 | Injury of intercostal blood vessels | 73 | 60 | 82.19 |
| S257 | Injury of multiple blood vessels of thorax | 5 | 3 | 60.00 |
| S258 | Injury of other blood vessels of thorax | 71 | 58 | 81.69 |
| S259 | Injury of unspecified blood vessel of thorax | 30 | 26 | 86.67 |
| S26 | Injury of heart | 836 | 724 | 86.60 |
| S260 | Injury of heart with hemopericardium | 197 | 148 | 75.13 |
| S268 | Other injuries of heart | 299 | 265 | 88.63 |
| S269 | Injury of heart, unspecified | 340 | 311 | 91.47 |
| S27 | injury of other and unspecified intrathoracic organs | 28033 | 26035 | 92.87 |
| S270 | Traumatic pneumothorax | 6327 | 6032 | 95.34 |
| S271 | Traumatic hemothorax | 8623 | 8097 | 93.90 |
| S272 | Traumatic hemopneumothorax | 6949 | 6423 | 92.43 |
| S273 | Other injuries of lung | 5098 | 4573 | 89.70 |
| S274 | Injury of bronchus | 18 | 12 | 66.67 |
| S275 | Injury of thoracic trachea | 81 | 70 | 86.42 |
| S276 | Injury of pleura | 55 | 54 | 98.18 |
| S277 | Multiple injuries of intrathoracic organs | 22 | 21 | 95.45 |
| S278 | Injury of other specified intrathoracic organs | 744 | 643 | 86.42 |
| S279 | Injury of unspecified intrathoracic organ | 78 | 76 | 97.44 |
| S28 | Crushing injury of thorax and traumatic amputation of part of thorax | 46 | 44 | 95.65 |
| S280 | Crushed chest | 46 | 44 | 95.65 |
| S281 | Traumatic amputation of part of thorax | 0 | 0 | 0.00 |
| S29 | Other and unspecified injuries of thorax | 351 | 346 | 98.58 |
| S290 | Injury of muscle and tendon at thorax level | 86 | 85 | 98.84 |
| S297 | Multiple injuries of thorax | 6 | 6 | 100.00 |
| S298 | Other specified injuries of thorax | 33 | 33 | 100.00 |
| S299 | Unspecified injury of thorax | 226 | 222 | 98.23 |
| S30 | Superficial injury of abdomen, lower back and pelvis | 27773 | 27557 | 99.22 |
| S300 | Contusion of lower back and pelvis | 15709 | 15628 | 99.48 |
| S301 | Contusion of abdominal wall | 9995 | 9881 | 98.86 |
| S302 | Contusion of external genital organs | 867 | 861 | 99.31 |
| S307 | Multiple superficial injuries of abdomen, lower back and pelvis | 64 | 63 | 98.44 |
| S308 | Other superficial injuries of abdomen, lower back and pelvis | 929 | 917 | 98.71 |
| S309 | Superficial injury of abdomen, lower back and pelvis, part unspecified | 203 | 201 | 99.01 |
| S31 | Open wound of abdomen, lower back and pelvis | 3714 | 3642 | 98.06 |
| S32 | Fracture of lumbar spine and pelvis | 87634 | 85098 | 97.11 |
| S320 | Fracture of lumbar vertebra | 57086 | 56186 | 98.42 |
| S321 | Fracture of sacrum | 5690 | 5480 | 96.31 |
| S322 | Fracture of coccyx | 1468 | 1459 | 99.39 |
| S323 | Fracture of ilium | 2540 | 2395 | 94.29 |
| S324 | Fracture of acetabulum | 4532 | 4360 | 96.20 |
| S325 | Fracture of pubis | 7165 | 6845 | 95.53 |
| S327 | Multiple fractures of lumbar spine and pelvis | 2198 | 2076 | 94.45 |
| S328 | Fracture of other and unspecified parts of lumbar spine and pelvis | 6809 | 6154 | 90.38 |
| S33 | Dislocation, sprain and strain of joints and ligaments of lumbar spine and pelvis | 97852 | 97722 | 99.87 |
| S330 | Traumatic rupture of lumbar intervertebral disc | 911 | 910 | 99.89 |
| S331 | Dislocation of lumbar vertebra | 461 | 453 | 98.26 |
| S332 | Dislocation of sacroiliac and sacrococcygeal joint | 185 | 175 | 94.59 |
| S333 | Dislocation of other and unspecified parts of lumbar spine and pelvis | 66 | 63 | 95.45 |
| S334 | Traumatic rupture of symphysis pubis | 132 | 124 | 93.94 |
| S335 | Sprain and strain of lumbar spine | 90676 | 90585 | 99.90 |
| S336 | Sprain and strain of sacroiliac joint | 301 | 296 | 98.34 |
| S337 | Sprain and strain of other and unspecified parts of lumbar spine and pelvis | 5105 | 5101 | 99.92 |
| S34 | Injury of nerves and lumbar spinal cord at abdomen, lower back and pelvis level | 590 | 574 | 97.29 |
| S340 | Concussion and oedema of lumbar spinal cord | 89 | 88 | 98.88 |
| S341 | Other injury of lumbar spinal cord | 312 | 303 | 97.12 |
| S342 | Injury of nerve root of lumbar and sacral spine | 94 | 93 | 98.94 |
| S343 | Injury of cauda equina | 39 | 38 | 97.44 |
| S344 | Injury of lumbosacral plexus | 28 | 28 | 100.00 |
| S345 | Injury of lumbar, sacral and pelvic sympathetic nerves | 15 | 12 | 80.00 |
| S346 | Injury of peripheral nerve(s) of abdomen, lower back and pelvis | 3 | 3 | 100.00 |
| S348 | Injury of other and unspecified nerves at abdomen, lower back and pelvis level | 8 | 7 | 87.50 |
| S35 | Injury of blood vessels at abdomen, lower back and pelvis level | 1830 | 1407 | 76.89 |
| S350 | Injury of abdominal aorta | 87 | 58 | 66.67 |
| S351 | Injury of inferior vena cava | 113 | 65 | 57.52 |
| S352 | Injury of coeliac or mesenteric artery | 585 | 484 | 82.74 |
| S353 | Injury of portal or splenic vein | 177 | 130 | 73.45 |
| S354 | Injury of renal blood vessels | 157 | 120 | 76.43 |
| S355 | Injury of iliac blood vessels | 268 | 169 | 63.06 |
| S357 | Injury of multiple blood vessels at abdomen, lower back and pelvis level | 35 | 21 | 60.00 |
| S358 | Injury of other blood vessels at abdomen, lower back and pelvis level | 231 | 208 | 90.04 |
| S359 | Injury of unspecified blood vessel at abdomen, lower back and pelvis level | 176 | 151 | 85.80 |
| S36 | Injury of intra-abdominal organs | 18005 | 15871 | 88.15 |
| S360 | Injury of spleen | 3241 | 2942 | 90.77 |
| S361 | Injury of liver or gallbladder | 6018 | 5417 | 90.01 |
| S362 | Injury of pancreas | 722 | 622 | 86.15 |
| S363 | Injury of stomach | 243 | 211 | 86.83 |
| S364 | Injury of small intestine | 1780 | 1576 | 88.54 |
| S365 | Injury of colon | 792 | 670 | 84.60 |
| S366 | Injury of rectum | 191 | 171 | 89.53 |
| S367 | Injury of multiple intra-abdominal organs | 139 | 91 | 65.47 |
| S368 | Injury of other intra-abdominal organs | 4524 | 3855 | 85.21 |
| S369 | Injury of unspecified intra-abdominal organ | 329 | 295 | 89.67 |
| S37 | Injury of urinary and pelvic organs | 4457 | 4127 | 92.60 |
| S370 | Injury of kidney | 2482 | 2319 | 93.43 |
| S371 | Injury of ureter | 66 | 59 | 89.39 |
| S372 | Injury of bladder | 476 | 409 | 85.92 |
| S373 | Injury of urethra | 882 | 846 | 95.92 |
| S374 | Injury of ovary | 6 | 5 | 83.33 |
| S375 | Injury of fallopian tube | 1 | 1 | 100.00 |
| S376 | Injury of uterus | 12 | 12 | 100.00 |
| S377 | Injury of multiple pelvic organs | 16 | 10 | 62.50 |
| S378 | Injury of other pelvic organs | 454 | 411 | 90.53 |
| S379 | Injury of unspecified pelvic organ | 51 | 44 | 86.27 |
| S38 | Crushing injury and traumatic amputation of part of abdomen, lower back and pelvis | 98 | 92 | 93.88 |
| S380 | Crushing injury of external genital organs | 10 | 10 | 100.00 |
| S381 | Crushing injury of other and unspecified parts of abdomen, lower back and pelvis | 55 | 49 | 89.09 |
| S382 | Traumatic amputation of external genital organs | 32 | 32 | 100.00 |
| S383 | Traumatic amputation of other and unspecified parts of abdomen, lower back and pelvis | 1 | 1 | 100.00 |
| S39 | Other and unspecified injuries of abdomen, lower back and pelvis | 1481 | 1439 | 97.16 |
| S390 | Injury of muscle and tendon of abdomen, lower back and pelvis | 305 | 293 | 96.07 |
| S396 | Injury of intra-abdominal organs(s) with pelvic organ(s) | 19 | 14 | 73.68 |
| S397 | Other multiple injuries of abdomen, lower back and pelvis | 15 | 13 | 86.67 |
| S398 | Other specified injuries of abdomen, lower back and pelvis | 223 | 217 | 97.31 |
| S399 | Unspecified injury of abdomen, lower back and pelvis | 916 | 899 | 98.14 |
| S40 | Superficial injury of shoulder and upper arm | 20066 | 20027 | 99.81 |
| S400 | Contusion of shoulder and upper arm | 19318 | 19281 | 99.81 |
| S407 | Multiple superficial injuries of shoulder and upper arm | 58 | 57 | 98.28 |
| S408 | Other superficial injuries of shoulder and upper arm | 597 | 596 | 99.83 |
| S409 | Superficial injury of shoulder and upper arm, unspecified | 92 | 92 | 100.00 |
| S41 | Open wound of shoulder and upper arm | 1133 | 1126 | 99.38 |
| S410 | Open wound of shoulder | 282 | 280 | 99.29 |
| S411 | Open wound of upper arm | 670 | 668 | 99.70 |
| S417 | Multiple open wounds of shoulder and upper arm | 109 | 109 | 100.00 |
| S418 | Open wound of other and unspecified parts of shoulder girdle | 67 | 64 | 95.52 |
| S42 | Fracture of shoulder and upper arm | 51625 | 50764 | 98.33 |
| S420 | Fracture of clavicle | 17742 | 17386 | 97.99 |
| S421 | Fracture of scapula | 5607 | 5435 | 96.93 |
| S422 | Fracture of upper end of humerus | 11490 | 11366 | 98.92 |
| S423 | Fracture of shaft of humerus | 6514 | 6353 | 97.53 |
| S424 | Fracture of lower end of humerus | 9886 | 9849 | 99.63 |
| S427 | Multiple fractures of clavicle, scapula and humerus | 72 | 68 | 94.44 |
| S428 | Fracture of other parts of shoulder and upper arm | 97 | 97 | 100.00 |
| S429 | Fracture of shoulder girdle, part unspecified | 158 | 153 | 96.84 |
| S43 | Dislocation, sprain and strain of joints and ligaments of shoulder girdle | 21298 | 21270 | 99.87 |
| S430 | Dislocation of shoulder joint | 2288 | 2271 | 99.26 |
| S431 | Anterior dislocation of humerus | 1305 | 1304 | 99.92 |
| S432 | Dislocation of sternoclavicular joint | 45 | 45 | 100.00 |
| S433 | Dislocation of other and unspecified parts of shoulder girdle | 189 | 187 | 98.94 |
| S434 | Sprain and strain of shoulder joint | 13818 | 13814 | 99.97 |
| S435 | Sprain and strain of acromioclavicular joint | 1252 | 1251 | 99.92 |
| S436 | Sprain and strain of sternoclavicular joint | 28 | 28 | 100.00 |
| S437 | Sprain and strain of other and unspecified | 2361 | 2358 | 99.87 |
| S439 | parts of shoulder girdle | 0 | 0 | 0.00 |
| S44 | Injury of nerves at shoulder and upper arm level | 496 | 495 | 99.80 |
| S440 | Injury of ulnar nerve at upper arm level | 63 | 63 | 100.00 |
| S441 | Injury of median nerve at upper arm level | 45 | 45 | 100.00 |
| S442 | Injury of radial nerve at upper arm level | 252 | 252 | 100.00 |
| S443 | Injury of axillary nerve | 49 | 49 | 100.00 |
| S444 | Injury of musculocutaneous nerve | 25 | 25 | 100.00 |
| S445 | Injury of cutaneous sensory nerve at shoulder and upper arm level | 13 | 13 | 100.00 |
| S447 | Injury of multiple nerves at shoulder and upper arm level | 11 | 10 | 90.91 |
| S448 | Injury of other nerves at shoulder and upper arm level | 25 | 25 | 100.00 |
| S449 | Injury of unspecified nerve at shoulder and upper arm level | 12 | 12 | 100.00 |
| S45 | Injury of blood vessels at shoulder and upper arm level | 311 | 302 | 97.11 |
| S450 | Injury of axillary artery | 22 | 20 | 90.91 |
| S451 | Injury of brachial artery | 143 | 141 | 98.60 |
| S452 | Injury of axillary or brachial vein | 38 | 36 | 94.74 |
| S453 | Injury of superficial vein at shoulder and upper arm level | 30 | 30 | 100.00 |
| S457 | Injury of multiple blood vessels at shoulder and upper arm level | 7 | 7 | 100.00 |
| S458 | Injury of other blood vessels at shoulder and upper arm level | 47 | 46 | 97.87 |
| S459 | Injury of unspecified blood vessel at shoulder and upper arm level | 23 | 21 | 91.30 |
| S46 | Injury of muscle and tendon at shoulder and upper arm level | 3663 | 3661 | 99.95 |
| S460 | Injury of muscle(s) and tendon(s) of the rotator cuff of shoulder | 2045 | 2044 | 99.95 |
| S461 | Injury of muscle and tendon of long head of biceps | 181 | 181 | 100.00 |
| S462 | Injury of muscle and tendon of other parts of biceps | 244 | 244 | 100.00 |
| S463 | Injury of muscle and tendon of triceps | 284 | 284 | 100.00 |
| S467 | Injury of multiple muscles and tendons at shoulder and upper arm level | 182 | 182 | 100.00 |
| S468 | Injury of other muscles and tendon at shoulder and upper arm level | 517 | 517 | 100.00 |
| S469 | Injury of unspecified muscle and tendon at shoulder and upper arm level | 209 | 208 | 99.52 |
| S47 | Crushing injury of shoulder and upper arm | 93 | 89 | 95.70 |
| S48 | Traumatic amputation of shoulder and upper arm | 55 | 51 | 92.73 |
| S480 | Traumatic amputation at shoulder joint | 6 | 6 | 100.00 |
| S481 | Traumatic amputation at level between shoulder and elbow | 29 | 26 | 89.66 |
| S489 | Traumatic amputation of shoulder and upper arm, level unspecified | 19 | 18 | 94.74 |
| S49 | Other and unspecified injuries of shoulder and upper arm | 252 | 251 | 99.60 |
| S497 | Multiple injuries of shoulder and upper arm | 10 | 10 | 100.00 |
| S498 | Other specified injuries of shoulder and upper arm | 76 | 76 | 100.00 |
| S499 | Unspecified injury of shoulder and upper arm | 166 | 165 | 99.40 |
| S50 | Superficial injury of forearm | 15699 | 15661 | 99.76 |
| S500 | Contusion of elbow | 10094 | 10074 | 99.80 |
| S501 | Contusion of other and unspecified parts of forearm | 3226 | 3219 | 99.78 |
| S507 | Multiple superficial injuries of forearm | 182 | 179 | 98.35 |
| S508 | Other superficial injuries of forearm | 1722 | 1716 | 99.65 |
| S509 | Superficial injury of forearm, unspecified | 473 | 471 | 99.58 |
| S51 | Open wound of forearm | 6038 | 5997 | 99.32 |
| S510 | Open wound of elbow | 2013 | 1995 | 99.11 |
| S517 | Multiple open wounds of forearm | 575 | 571 | 99.30 |
| S518 | Open wound of other parts of forearm | 1395 | 1388 | 99.50 |
| S519 | Open wound of forearm, part unspecified | 2036 | 2024 | 99.41 |
| S52 | Fracture of forearm | 48773 | 48480 | 99.40 |
| S520 | Fracture of upper end of ulna | 5728 | 5674 | 99.06 |
| S521 | Fracture of upper end of radius | 3746 | 3729 | 99.55 |
| S522 | Fracture of shaft of ulna | 1646 | 1629 | 98.97 |
| S523 | Fracture of shaft of radius | 1588 | 1572 | 98.99 |
| S524 | Fracture of shafts of both ulna and radius | 2202 | 2188 | 99.36 |
| S525 | Fracture of lower end of radius | 21532 | 21435 | 99.55 |
| S526 | Fracture of lower end of both ulna and radius | 9148 | 9104 | 99.52 |
| S527 | Multiple fracture of forearm | 343 | 339 | 98.83 |
| S528 | Fracture of other parts of forearm | 2292 | 2269 | 99.00 |
| S529 | Fracture of forearm, part unspecified | 507 | 501 | 98.82 |
| S53 | Dislocation, sprain and strain of joints and ligaments of elbow | 6268 | 6256 | 99.81 |
| S530 | Dislocation of radial head | 139 | 138 | 99.28 |
| S531 | Dislocation of elbow, unspecified | 1584 | 1577 | 99.56 |
| S532 | Traumatic rupture of radial collateral ligament | 632 | 632 | 100.00 |
| S533 | Traumatic rupture of ulnar collateral ligament | 792 | 791 | 99.87 |
| S534 | Sprain and strain of elbow | 3116 | 3113 | 99.90 |
| S54 | Injury of nerves at forearm level | 1487 | 1484 | 99.80 |
| S540 | Injury of ulnar nerve at forearm level | 447 | 446 | 99.78 |
| S541 | Injury of median nerve at forearm level | 298 | 297 | 99.66 |
| S542 | Injury of radial nerve at forearm level | 516 | 515 | 99.81 |
| S543 | Injury of cutaneous sensory nerve at forearm level | 76 | 76 | 100.00 |
| S547 | Injury of multiple nerves at forearm level | 33 | 33 | 100.00 |
| S548 | Injury of other nerves at forearm level | 82 | 82 | 100.00 |
| S549 | Injury of unspecified nerve at forearm level | 33 | 33 | 100.00 |
| S55 | Injury of blood vessels at forearm level | 981 | 971 | 98.98 |
| S550 | Injury of ulnar artery at forearm level | 237 | 237 | 100.00 |
| S551 | Injury of radial artery at forearm level | 398 | 393 | 98.74 |
| S552 | Injury of vein at forearm level | 167 | 167 | 100.00 |
| S557 | Injury of multiple blood vessels at forearm level | 15 | 15 | 100.00 |
| S558 | Injury of other blood vessels at forearm level | 127 | 122 | 96.06 |
| S559 | Injury of unspecified blood vessels at forearm level | 36 | 36 | 100.00 |
| S56 | Injury of muscle and tendon at forearm level | 5995 | 5984 | 99.82 |
| S560 | Injury of flexor muscle and tendon of thumb at forearm level | 138 | 138 | 100.00 |
| S561 | Injury of flexor muscle and tendon of other finger(s) at forearm level | 799 | 799 | 100.00 |
| S562 | Injury of other flexor muscle and tendon at forearm level | 1388 | 1384 | 99.71 |
| S563 | Injury of extensor or abductor muscles and tendons of thumb at forearm level | 362 | 362 | 100.00 |
| S564 | Injury of extensor muscle and tendon of other finger(s) at forearm level | 1010 | 1008 | 99.80 |
| S565 | Injury of other extensor muscle and tendon at forearm level | 938 | 938 | 100.00 |
| S567 | Injury of multiple muscles and tendons at forearm level | 542 | 541 | 99.82 |
| S568 | Injury of other and unspecified muscles and tendon at forearm level | 808 | 804 | 99.50 |
| S57 | Crushing injury of forearm | 373 | 368 | 98.66 |
| S570 | Crushing injury of elbow | 92 | 89 | 96.74 |
| S578 | Crushing injury of other parts of forearm | 139 | 138 | 99.28 |
| S579 | Crushing injury of forearm, part unspecified | 140 | 139 | 99.29 |
| S58 | Traumatic amputation of forearm | 97 | 94 | 96.91 |
| S580 | Traumatic amputation at elbow level | 18 | 18 | 100.00 |
| S581 | Traumatic amputation at level between elbow and wrist | 33 | 32 | 96.97 |
| S589 | Traumatic amputation of forearm, level unspecified | 46 | 44 | 95.65 |
| S59 | Other and unspecified injuries of forearm | 228 | 226 | 99.12 |
| S597 | Multiple injuries of forearm | 37 | 37 | 100.00 |
| S598 | Other specified injuries of forearm | 97 | 97 | 100.00 |
| S599 | Unspecified injury of forearm | 93 | 91 | 97.85 |
| S60 | Superficial injury of wrist and hand | 13242 | 13207 | 99.74 |
| S600 | Contusion of finger(s) without damage to nail | 1535 | 1535 | 100.00 |
| S601 | Contusion of finger(s) with damage to nail | 235 | 235 | 100.00 |
| S602 | Contusion of other parts of wrist and hand | 8527 | 8505 | 99.74 |
| S607 | Multiple superficial injuries of wrist and hand | 178 | 176 | 98.88 |
| S608 | Other superficial injuries of wrist and hand | 2338 | 2328 | 99.57 |
| S609 | Superficial injury of wrist and hand, unspecified | 419 | 418 | 99.76 |
| S61 | Open wound of wrist and hand | 28430 | 28343 | 99.69 |
| S610 | Open wound of finger(s) without damage to nail | 12092 | 12078 | 99.88 |
| S611 | Open wound of finger(s) with damage to nail | 4790 | 4789 | 99.98 |
| S617 | Multiple open wounds of wrist and hand | 1816 | 1802 | 99.23 |
| S618 | Open wound of other parts of wrist and hand | 4810 | 4781 | 99.40 |
| S619 | Open wound of wrist and hand, part unspecified | 4887 | 4859 | 99.43 |
| S62 | Fracture at wrist and hand level | 34667 | 34553 | 99.67 |
| S620 | Fracture of navicular [scaphoid] bone of hand | 1115 | 1111 | 99.64 |
| S621 | Fracture of other carpal bone(s) | 2428 | 2420 | 99.67 |
| S622 | Fracture of first metacarpal bone | 1071 | 1064 | 99.35 |
| S623 | Fracture of other metacarpal bone | 5535 | 5494 | 99.26 |
| S624 | Multiple fracture of metacarpal bones | 513 | 507 | 98.83 |
| S625 | Fracture of thumb | 3219 | 3217 | 99.94 |
| S626 | Fracture of other finger | 19108 | 19079 | 99.85 |
| S627 | Multiple fracture of fingers | 706 | 705 | 99.86 |
| S628 | Fracture of other and unspecified parts of wrist and hand | 937 | 921 | 98.29 |
| S63 | Dislocation, sprain and strain of joints and ligaments at wrist and hand level | 10910 | 10900 | 99.91 |
| S630 | Dislocation of wrist | 684 | 683 | 99.85 |
| S631 | Dislocation of finger | 1077 | 1071 | 99.44 |
| S632 | Multiple dislocations of fingers | 27 | 27 | 100.00 |
| S633 | Traumatic rupture of ligament of wrist and carpus | 583 | 583 | 100.00 |
| S634 | Traumatic rupture of ligament of finger at metacarpophalangeal and interphalangeal joint(s) | 1142 | 1142 | 100.00 |
| S635 | Sprain and strain of wrist | 5104 | 5102 | 99.96 |
| S636 | Sprain and strain of finger(s) | 1488 | 1487 | 99.93 |
| S637 | Sprain and strain of other and unspecified parts of hand | 799 | 799 | 100.00 |
| S64 | Injury of nerves at wrist and hand level | 6503 | 6502 | 99.98 |
| S640 | Injury of ulnar nerve at wrist and hand level | 595 | 595 | 100.00 |
| S641 | Injury of median nerve at wrist and hand level | 550 | 550 | 100.00 |
| S642 | Injury of radial nerve at wrist and hand level | 584 | 584 | 100.00 |
| S643 | Injury of digital nerve of thumb | 833 | 832 | 99.88 |
| S644 | Injury of digital nerve of other finger | 3449 | 3449 | 100.00 |
| S647 | Injury of multiple nerves at wrist and hand level | 139 | 139 | 100.00 |
| S648 | Injury of other nerves at wrist and hand level | 228 | 228 | 100.00 |
| S649 | Injury of unspecified nerve at wrist and hand level | 119 | 119 | 100.00 |
| S65 | Injury of blood vessels at wrist and hand level | 4030 | 4027 | 99.93 |
| S650 | Injury of ulnar artery at wrist and hand level | 458 | 458 | 100.00 |
| S651 | Injury of radial artery at wrist and hand level | 622 | 622 | 100.00 |
| S652 | Injury of superficial palmar arch | 46 | 46 | 100.00 |
| S653 | Injury of deep palmar arch | 27 | 26 | 96.30 |
| S654 | Injury of blood vessel(s) of thumb | 378 | 377 | 99.74 |
| S655 | Injury of blood vessel(s) of other finger | 1959 | 1959 | 100.00 |
| S657 | Injury of multiple blood vessels at wrist and hand level | 70 | 70 | 100.00 |
| S658 | Injury of other blood vessels at wrist and hand level | 350 | 350 | 100.00 |
| S659 | Injury of unspecified blood vessel at wrist and hand level | 117 | 116 | 99.15 |
| S66 | Injury of muscle and tendon at wrist and hand level | 24138 | 24130 | 99.97 |
| S660 | Injury of long flexor muscle and tendon of thumb at wrist and hand level | 787 | 786 | 99.87 |
| S661 | Injury of flexor muscle and tendon of other finger at wrist and hand level | 4317 | 4313 | 99.91 |
| S662 | Injury of extensor muscle and tendon of thumb at wrist and hand level | 2752 | 2752 | 100.00 |
| S663 | Injury of extensor muscle and tendon of other finger at wrist and hand level | 10591 | 10591 | 100.00 |
| S664 | Injury of intrinsic muscle and tendon of thumb at wrist and hand level | 482 | 482 | 100.00 |
| S665 | Injury of intrinsic muscle and tendon of other finger at wrist and hand level | 556 | 556 | 100.00 |
| S666 | Injury of multiple flexor muscles and tendons at wrist and hand level | 1081 | 1081 | 100.00 |
| S667 | Injury of multiple extensor muscles and tendons at wrist and hand level | 971 | 970 | 99.90 |
| S668 | Injury of other muscles and tendons at wrist and hand level | 1894 | 1894 | 100.00 |
| S669 | Injury of unspecified muscle and tendon at wrist and hand level | 686 | 684 | 99.71 |
| S67 | Crushing injury of wrist and hand | 5974 | 5968 | 99.90 |
| S670 | Crushing injury of thumb and other finger(s) | 4959 | 4957 | 99.96 |
| S678 | Crushing injury of other and unspecified parts of wrist and hand | 1008 | 1004 | 99.60 |
| S68 | Traumatic amputation of wrist and hand | 7231 | 7227 | 99.94 |
| S680 | Traumatic amputation of thumb (complete) (partial) | 925 | 925 | 100.00 |
| S681 | Traumatic amputation of other single finger (complete)(partial) | 5325 | 5322 | 99.94 |
| S682 | Traumatic amputation of two or more fingers alone (complete)(partial) | 610 | 610 | 100.00 |
| S683 | Combined traumatic amputation of (part of) finger(s) with other parts of wrist and hand | 159 | 159 | 100.00 |
| S684 | Traumatic amputation of hand at wrist level | 46 | 46 | 100.00 |
| S688 | Traumatic amputation of other parts of wrist and hand | 97 | 96 | 98.97 |
| S689 | Traumatic amputation of wrist and hand, level unspecified | 61 | 61 | 100.00 |
| S69 | Other and unspecified injuries of wrist and hand | 1085 | 1082 | 99.72 |
| S697 | Multiple injuries of wrist and hand | 84 | 83 | 98.81 |
| S698 | Other specified injuries of wrist and hand | 437 | 437 | 100.00 |
| S699 | Unspecified injury of wrist and hand | 563 | 561 | 99.64 |
| S70 | Superficial injury of hip and thigh | 17185 | 17076 | 99.37 |
| S700 | Contusion of hip | 10683 | 10603 | 99.25 |
| S701 | Contusion of thigh | 5565 | 5546 | 99.66 |
| S707 | Multiple superficial injuries of hip and thigh | 61 | 59 | 96.72 |
| S708 | Other superficial injuries of hip and thigh | 672 | 666 | 99.11 |
| S709 | Superficial injury of hip and thigh, unspecified | 200 | 198 | 99.00 |
| S71 | Open wound of hip and thigh | 2032 | 2003 | 98.57 |
| S710 | Open wound of hip | 229 | 227 | 99.13 |
| S711 | Open wound of thigh | 1668 | 1644 | 98.56 |
| S717 | Multiple open wounds of hip and thigh | 114 | 111 | 97.37 |
| S718 | Open wound of other and unspecified parts of pelvic girdle | 18 | 18 | 100.00 |
| S72 | Fracture of femur | 84194 | 81472 | 96.77 |
| S720 | Fracture of neck of femur | 28548 | 27711 | 97.07 |
| S721 | Pertrochanteric fracture | 34979 | 33767 | 96.54 |
| S722 | Subtrochanteric fracture | 2819 | 2720 | 96.49 |
| S723 | Fracture of shaft of femur | 7454 | 7252 | 97.29 |
| S724 | Fracture of lower end of femur | 5210 | 5110 | 98.08 |
| S727 | Multiple fractures of femur | 130 | 118 | 90.77 |
| S728 | Fractures of other parts of femur | 1070 | 1027 | 95.98 |
| S729 | Fracture of femur, part unspecified | 3824 | 3610 | 94.40 |
| S73 | Dislocation, sprain and strain of joint and ligaments of hip | 4565 | 4524 | 99.10 |
| S730 | Dislocation of hip | 1731 | 1698 | 98.09 |
| S731 | Sprain and strain of hip | 2833 | 2825 | 99.72 |
| S74 | Injury of nerves at hip and thigh level | 174 | 173 | 99.43 |
| S740 | Injury of sciatic nerve at hip and thigh level | 97 | 96 | 98.97 |
| S741 | Injury of femoral nerve at hip and thigh level | 31 | 31 | 100.00 |
| S742 | Injury of cutaneous sensory nerve at hip and thigh level | 18 | 18 | 100.00 |
| S747 | Injury of multiple nerves at hip and thigh level | 2 | 2 | 100.00 |
| S748 | Injury of other nerves at hip and thigh level | 16 | 16 | 100.00 |
| S749 | Injury of unspecified nerve at hip and thigh level | 8 | 8 | 100.00 |
| S75 | Injury of blood vessels at hip and thigh level | 471 | 432 | 91.72 |
| S750 | Injury of femoral artery | 182 | 156 | 85.71 |
| S751 | Injury of femoral vein at hip and thigh level | 61 | 56 | 91.80 |
| S752 | Injury of greater saphenous vein at hip and thigh level | 14 | 14 | 100.00 |
| S757 | Injury of multiple blood vessels at hip and thigh level | 9 | 9 | 100.00 |
| S758 | Injury of other blood vessels at hip and thigh level | 101 | 97 | 96.04 |
| S759 | Injury of unspecified blood vessel at hip and thigh level | 104 | 100 | 96.15 |
| S76 | Injury of muscle and tendon at hip and thigh level | 4170 | 4162 | 99.81 |
| S760 | Injury of muscle and tendon of hip | 331 | 329 | 99.40 |
| S761 | Injury of quadriceps muscle and tendon | 1867 | 1866 | 99.95 |
| S762 | Injury of adductor muscle and tendon of thigh | 259 | 257 | 99.23 |
| S763 | Injury of muscle and tendon of the posterior muscle group at thigh level | 336 | 335 | 99.70 |
| S764 | Injury of other and unspecified muscles and tendons at thigh level | 1067 | 1065 | 99.81 |
| S767 | Injury of multiple muscles and tendons at hip and thigh level | 304 | 304 | 100.00 |
| S77 | Crushing injury of hip and thigh | 121 | 116 | 95.87 |
| S770 | Crushing injury of hip | 11 | 11 | 100.00 |
| S771 | Crushing injury of thigh | 88 | 84 | 95.45 |
| S772 | Crushing injury of hip with thigh | 22 | 21 | 95.45 |
| S78 | Traumatic amputation of hip and thigh | 25 | 23 | 92.00 |
| S780 | Traumatic amputation at hip joint | 1 | 1 | 100.00 |
| S781 | Traumatic amputation at level between hip and knee | 20 | 18 | 90.00 |
| S789 | Traumatic amputation of hip and thigh, level unspecified | 4 | 4 | 100.00 |
| S79 | Other and unspecified injuries of hip and thigh | 151 | 147 | 97.35 |
| S797 | Multiple injuries of hip and thigh | 14 | 14 | 100.00 |
| S798 | Other specified injuries of hip and thigh | 40 | 40 | 100.00 |
| S799 | Unspecified injury of hip and thigh | 96 | 93 | 96.88 |
| S80 | Superficial injury of lower leg | 40076 | 39987 | 99.78 |
| S800 | Contusion of knee | 27075 | 27030 | 99.83 |
| S801 | Contusion of other and unspecified parts of lower leg | 8775 | 8759 | 99.82 |
| S807 | Multiple superficial injuries of lower leg | 368 | 363 | 98.64 |
| S808 | Other superficial injuries of lower leg | 3274 | 3253 | 99.36 |
| S809 | Superficial injury of lower leg, unspecified | 574 | 572 | 99.65 |
| S81 | Open wound of lower leg | 11078 | 10994 | 99.24 |
| S810 | Open wound of knee | 5111 | 5081 | 99.41 |
| S817 | Multiple open wounds of lower leg | 689 | 680 | 98.69 |
| S818 | Open wound of other parts of lower leg | 2354 | 2333 | 99.11 |
| S819 | Open wound of lower leg, part unspecified | 2893 | 2869 | 99.17 |
| S82 | Fracture of lower leg, including ankle | 75679 | 74889 | 98.96 |
| S820 | Fracture of patella | 9964 | 9909 | 99.45 |
| S821 | Fracture of upper end of tibia | 8823 | 8703 | 98.64 |
| S822 | Fracture of shaft of tibia | 10907 | 10653 | 97.67 |
| S823 | Fracture of lower end of tibia | 10460 | 10359 | 99.03 |
| S824 | Fracture of fibula alone | 6005 | 5933 | 98.80 |
| S825 | Fracture of medial malleolus | 4924 | 4873 | 98.96 |
| S826 | Fracture of lateral malleolus | 10362 | 10329 | 99.68 |
| S827 | Multiple fractures of lower leg | 255 | 247 | 96.86 |
| S828 | Fractures of other parts of lower leg | 13476 | 13395 | 99.40 |
| S829 | Fracture of lower leg, part unspecified | 360 | 348 | 96.67 |
| S83 | Dislocation, sprain and strain of joints and ligaments of knee | 23049 | 23026 | 99.90 |
| S830 | Dislocation of patella | 414 | 414 | 100.00 |
| S831 | Dislocation of knee | 230 | 227 | 98.70 |
| S832 | Tear of meniscus, current | 2984 | 2984 | 100.00 |
| S833 | Tear of articular cartilage of knee, current | 150 | 150 | 100.00 |
| S834 | Sprain and strain involving (fibular)(tibial) collateral ligament of knee | 4763 | 4756 | 99.85 |
| S835 | Sprain and strain involving (anterior) (posterior) cruciate ligament of knee | 4568 | 4562 | 99.87 |
| S836 | Sprain and strain of other and unspecified parts of knee | 9510 | 9503 | 99.93 |
| S837 | Injury to multiple structures of knee | 407 | 407 | 100.00 |
| S84 | Injury of nerves at lower leg level | 764 | 764 | 100.00 |
| S840 | Injury of tibial nerve at lower leg level | 133 | 133 | 100.00 |
| S841 | Injury of peroneal nerve at lower leg level | 469 | 469 | 100.00 |
| S842 | Injury of cutaneous sensory nerve at lower leg level | 45 | 45 | 100.00 |
| S847 | Injury of multiple nerves at lower leg level | 11 | 11 | 100.00 |
| S848 | Injury of other nerves at lower leg level | 85 | 85 | 100.00 |
| S849 | Injury of unspecified nerve at lower leg level | 21 | 21 | 100.00 |
| S85 | Injury of blood vessels at lower leg level | 739 | 715 | 96.75 |
| S850 | Injury of popliteal artery | 115 | 106 | 92.17 |
| S851 | Injury of (anterior)(posterior) tibial artery | 227 | 219 | 96.48 |
| S852 | Injury of peroneal artery | 24 | 23 | 95.83 |
| S853 | Injury of greater saphenous vein at lower leg level | 46 | 46 | 100.00 |
| S854 | Injury of lesser saphenous vein at lower leg level | 18 | 18 | 100.00 |
| S855 | Injury of popliteal vein | 26 | 23 | 88.46 |
| S857 | Injury of multiple blood vessels at lower leg level | 14 | 14 | 100.00 |
| S858 | Injury of other blood vessels at lower leg level | 168 | 166 | 98.81 |
| S859 | Injury of unspecified blood vessel at lower leg level | 101 | 100 | 99.01 |
| S86 | Injury of muscle and tendon at lower leg level | 8545 | 8532 | 99.85 |
| S860 | Injury of Achilles tendon | 4060 | 4058 | 99.95 |
| S861 | Injury of other muscle(s) and tendon(s) of posterior muscle group at lower leg level | 932 | 930 | 99.79 |
| S862 | Injury of muscle(s) and tendon(s) of anterior muscle group at lower leg level | 857 | 857 | 100.00 |
| S863 | Injury of muscle(s) and tendon(s) of peroneal muscle group at lower leg level | 350 | 350 | 100.00 |
| S867 | Injury of multiple muscles and tendons at lower leg level | 453 | 448 | 98.90 |
| S868 | Injury of other muscles and tendons at lower leg level | 1244 | 1241 | 99.76 |
| S869 | Injury of unspecified muscles and tendons at lower leg level | 622 | 621 | 99.84 |
| S87 | Crushing injury of lower leg | 599 | 579 | 96.66 |
| S870 | Crushing injury of knee | 199 | 197 | 98.99 |
| S878 | Crushing injury of other and unspecified parts of lower leg | 400 | 382 | 95.50 |
| S88 | Traumatic amputation of lower leg | 88 | 82 | 93.18 |
| S880 | Traumatic amputation at knee level | 23 | 19 | 82.61 |
| S881 | Traumatic amputation at level between knee and ankle | 42 | 42 | 100.00 |
| S889 | Traumatic amputation of lower leg, level unspecified | 22 | 20 | 90.91 |
| S89 | Other and unspecified injuries of lower leg | 436 | 431 | 98.85 |
| S897 | Multiple injuries of lower leg | 18 | 17 | 94.44 |
| S898 | Other specified injuries of lower leg | 208 | 207 | 99.52 |
| S899 | Unspecified injury of lower leg | 210 | 207 | 98.57 |
| S90 | Superficial injury of ankle and foot | 11061 | 11036 | 99.77 |
| S900 | Contusion of ankle | 4329 | 4321 | 99.82 |
| S901 | Contusion of toe(s) without damage to nail | 631 | 630 | 99.84 |
| S902 | Contusion of toe(s) with damage to nail | 167 | 167 | 100.00 |
| S903 | Contusion of other and unspecified parts of foot | 4384 | 4377 | 99.84 |
| S907 | Multiple superficial injuries of ankle and foot | 145 | 144 | 99.31 |
| S908 | Other superficial injuries of ankle and foot | 1199 | 1191 | 99.33 |
| S909 | Superficial injury of ankle and foot, unspecified | 202 | 202 | 100.00 |
| S91 | Open wound of ankle and foot | 7270 | 7235 | 99.52 |
| S910 | Open wound of ankle | 1645 | 1633 | 99.27 |
| S911 | Open wound of toe(s) without damage to nail | 1028 | 1024 | 99.61 |
| S912 | Open wound of toe(s) with damage to nail | 574 | 573 | 99.83 |
| S913 | Open wound of other parts of foot | 3633 | 3617 | 99.56 |
| S917 | Multiple open wounds of ankle and foot | 370 | 368 | 99.46 |
| S92 | Fracture of foot, except ankle | 36263 | 36102 | 99.56 |
| S920 | Fracture of calcaneus | 9649 | 9598 | 99.47 |
| S921 | Fracture of talus | 1833 | 1821 | 99.35 |
| S922 | Fracture of other tarsal bone(s) | 5362 | 5337 | 99.53 |
| S923 | Fracture of metatarsal bone | 10802 | 10755 | 99.56 |
| S924 | Fracture of great toe | 3457 | 3450 | 99.80 |
| S925 | Fracture of other toe | 3973 | 3966 | 99.82 |
| S927 | Multiple fractures of foot | 616 | 612 | 99.35 |
| S929 | Fracture of foot, unspecified | 527 | 520 | 98.67 |
| S93 | Dislocation, sprain and strain of joints and ligaments at ankle and foot level | 19561 | 19548 | 99.93 |
| S930 | Dislocation of ankle joint | 1242 | 1240 | 99.84 |
| S931 | Dislocation of toe(s) | 512 | 511 | 99.80 |
| S932 | Rupture of ligaments at ankle and foot level | 3393 | 3391 | 99.94 |
| S933 | Dislocation of other and unspecified parts of foot | 881 | 880 | 99.89 |
| S934 | Sprain and strain of ankle | 11765 | 11759 | 99.95 |
| S935 | Sprain and strain of toe(s) | 219 | 219 | 100.00 |
| S936 | Sprain and strain of other and unspecified parts of foot | 1524 | 1523 | 99.93 |
| S94 | Injury of nerves at ankle and foot level | 470 | 469 | 99.79 |
| S940 | Injury of lateral plantar nerve | 6 | 6 | 100.00 |
| S941 | Injury of medial plantar nerve | 27 | 27 | 100.00 |
| S942 | Injury of deep peroneal nerve at ankle and foot level | 121 | 121 | 100.00 |
| S943 | Injury of cutaneous sensory nerve at ankle and foot level | 74 | 74 | 100.00 |
| S947 | Injury of multiple nerves at ankle and foot level | 14 | 14 | 100.00 |
| S948 | Injury of other nerves at ankle and foot level | 167 | 167 | 100.00 |
| S949 | Injury of unspecified nerve at ankle and foot level | 59 | 58 | 98.31 |
| S95 | Injury of blood vessels at ankle and foot level | 365 | 363 | 99.45 |
| S950 | Injury of dorsal artery of foot | 113 | 112 | 99.12 |
| S951 | Injury of plantar artery of foot | 28 | 27 | 96.43 |
| S952 | Injury of dorsal vein of foot | 21 | 21 | 100.00 |
| S957 | Injury of multiple blood vessels at ankle and foot level | 9 | 9 | 100.00 |
| S958 | Injury of other blood vessels at ankle and foot level | 137 | 137 | 100.00 |
| S959 | Injury of unspecified blood vessel at ankle and foot level | 56 | 56 | 100.00 |
| S96 | Injury of muscle and tendon at ankle and foot level | 3873 | 3866 | 99.82 |
| S960 | Injury of muscle and tendon of long flexor muscle of toe at ankle and foot level | 422 | 422 | 100.00 |
| S961 | Injury of muscle and tendon of long extensor muscle of toe at ankle and foot level | 1888 | 1887 | 99.95 |
| S962 | Injury of intrinsic muscle and tendon at ankle and foot level | 250 | 250 | 100.00 |
| S967 | Injury of multiple muscles and tendons at ankle and foot level | 205 | 203 | 99.02 |
| S968 | Injury of other muscles and tendons at ankle and foot level | 739 | 736 | 99.59 |
| S969 | Injury of unspecified muscle tendon at ankle and foot level | 365 | 364 | 99.73 |
| S97 | Crushing injury of ankle and foot | 1326 | 1318 | 99.40 |
| S970 | Crushing injury of ankle | 141 | 138 | 97.87 |
| S971 | Crushing injury of toe(s) | 327 | 326 | 99.69 |
| S978 | Crushing injury of other parts of ankle and foot | 851 | 847 | 99.53 |
| S98 | Traumatic amputation of ankle and foot | 255 | 252 | 98.82 |
| S980 | Traumatic amputation of foot at ankle level | 23 | 22 | 95.65 |
| S981 | Traumatic amputation of one toe | 132 | 132 | 100.00 |
| S982 | Traumatic amputation of two or more toes | 56 | 55 | 98.21 |
| S983 | Traumatic amputation of other parts of foot | 28 | 27 | 96.43 |
| S984 | Traumatic amputation of foot, level unspecified | 15 | 15 | 100.00 |
| S99 | Other and unspecified injuries of ankle and foot | 637 | 637 | 100.00 |
| S997 | Multiple injuries of ankle and foot | 26 | 26 | 100.00 |
| S998 | Other specified injuries of ankle and foot | 386 | 386 | 100.00 |
| S999 | Unspecified injury of ankle and foot | 220 | 220 | 100.00 |
| T00 | Superficial injuries involving multiple body regions | 33337 | 32970 | 98.90 |
| T000 | Superficial injuries involving head with neck | 35 | 34 | 97.14 |
| T001 | Superficial injuries involving thorax with abdomen, lower back and pelvis | 8 | 8 | 100.00 |
| T002 | Superficial injuries involving multiple regions of upper limb(s) | 28 | 28 | 100.00 |
| T003 | Superficial injuries involving multiple regions of lower limb(s) | 43 | 43 | 100.00 |
| T006 | Superficial injuries involving multiple regions of upper limb(s) with lower limb(s) | 70 | 70 | 100.00 |
| T008 | Superficial injuries involving other combinations of body regions | 681 | 680 | 99.85 |
| T009 | Multiple superficial injuries, unspecified | 32471 | 32106 | 98.88 |
| T01 | Open wounds involving multiple body regions | 1060 | 1009 | 95.19 |
| T010 | Open wounds involving head with neck | 21 | 20 | 95.24 |
| T011 | Open wounds involving thorax with abdomen, lower back and pelvis | 11 | 11 | 100.00 |
| T012 | Open wounds involving multiple regions of upper limb(s) | 34 | 34 | 100.00 |
| T013 | Open wounds involving multiple regions of lower limb(s) | 29 | 27 | 93.10 |
| T016 | Open wounds involving multiple regions of upper limb(s) with lower limb(s) | 16 | 16 | 100.00 |
| T018 | Open wounds involving other combinations of body regions | 30 | 29 | 96.67 |
| T019 | Multiple open wounds, unspecified | 917 | 870 | 94.87 |
| T02 | Fractures involving multiple body regions | 314 | 293 | 93.31 |
| T020 | Fractures involving head with neck | 3 | 2 | 66.67 |
| T021 | Fractures involving thorax with lower back and pelvis | 106 | 105 | 99.06 |
| T022 | Fractures involving multiple regions of one upper limb | 13 | 13 | 100.00 |
| T023 | Fractures involving multiple regions of one lower limb | 22 | 22 | 100.00 |
| T024 | Fractures involving multiple regions of both upper limbs | 2 | 2 | 100.00 |
| T025 | Fractures involving multiple regions of both lower limbs | 16 | 15 | 93.75 |
| T026 | Fractures involving multiple regions of upper limb(s) with lower limb(s) | 19 | 18 | 94.74 |
| T027 | Fractures involving thorax with lower back and pelvis with limb(s) | 16 | 16 | 100.00 |
| T028 | Fractures involving other combinations of body regions | 68 | 62 | 91.18 |
| T029 | Multiple fractures, unspecified | 48 | 37 | 77.08 |
| T03 | Dislocations, sprains and strains involving multiple body regions | 417 | 417 | 100.00 |
| T030 | Dislocations, sprains and strains involving head with neck | 24 | 24 | 100.00 |
| T031 | Dislocations, sprains and strains involving thorax with lower back and pelvis | 91 | 91 | 100.00 |
| T032 | Dislocations, sprains and strains involving multiple regions of upper limb(s) | 6 | 6 | 100.00 |
| T033 | Dislocations, sprains and strains involving multiple regions of lower limb(s) | 8 | 8 | 100.00 |
| T034 | Dislocations, sprains and strains involving multiple regions of upper limb(s) with lower limb(s) | 1 | 1 | 100.00 |
| T038 | Dislocations, sprains and strains involving other combinations of body regions | 83 | 83 | 100.00 |
| T039 | Multiple dislocations, sprains and strains, unspecified | 204 | 204 | 100.00 |
| T04 | Crushing injuries involving multiple body regions | 119 | 113 | 94.96 |
| T040 | Crushing injuries involving head with neck | 1 | 1 | 100.00 |
| T041 | Crushing injuries involving thorax with abdomen, lower back and pelvis | 6 | 6 | 100.00 |
| T042 | Crushing injuries involving multiple regions of upper limb(s) | 34 | 33 | 97.06 |
| T043 | Crushing injuries involving multiple regions of lower limb(s) | 57 | 54 | 94.74 |
| T044 | Crushing injuries involving multiple regions of upper limb(s) with lower limb(s) | 2 | 2 | 100.00 |
| T047 | Crushing injuries of thorax with abdomen, lower back and pelvis with limb(s) | 1 | 1 | 100.00 |
| T048 | Crushing injuries involving other combinations of body regions | 3 | 3 | 100.00 |
| T049 | Multiple crushing injuries, unspecified | 15 | 13 | 86.67 |
| T05 | Traumatic amputations involving multiple body regions | 30 | 28 | 93.33 |
| T050 | Traumatic amputation of both hands | 3 | 3 | 100.00 |
| T051 | Traumatic amputation of one hand and other arm [any level, except hand] | 7 | 7 | 100.00 |
| T052 | Traumatic amputation of both arm [any level] | 1 | 1 | 100.00 |
| T053 | Traumatic amputation of both feet | 1 | 1 | 100.00 |
| T054 | Traumatic amputation of one foot and other leg [any level, except foot] | 2 | 2 | 100.00 |
| T055 | Traumatic amputation of both legs [any level] | 4 | 2 | 50.00 |
| T056 | Traumatic amputation of upper and lower limbs, any combination [any level] | 1 | 1 | 100.00 |
| T058 | Traumatic amputation involving other combinations of body regions | 1 | 1 | 100.00 |
| T059 | Multiple traumatic amputations, unspecified | 10 | 10 | 100.00 |
| T06 | Other injuries involving multiple body regions, NEC | 907 | 831 | 91.62 |
| T060 | Injuries of brain and cranial nerves with injuries of nerves and spinal cord at neck level | 10 | 8 | 80.00 |
| T061 | Injuries of nerves and spinal cord involving other multiple body regions | 26 | 24 | 92.31 |
| T062 | Injuries of nerves involving multiple body regions | 4 | 4 | 100.00 |
| T063 | Injuries of blood vessels involving multiple body regions | 2 | 2 | 100.00 |
| T064 | Injuries of muscles and tendons involving multiple body regions | 47 | 47 | 100.00 |
| T065 | Injuries of intrathoracic organs with intra-abdominal and pelvic organs | 2 | 1 | 50.00 |
| T068 | Other specified injuries involving multiple body regions | 816 | 745 | 91.30 |
| T07 | Unspecified multiple injuries | 520 | 482 | 92.69 |
| T08 | Fracture of spine, level unspecified | 1105 | 1076 | 97.38 |
| T080 | Closed | 1093 | 1065 | 97.44 |
| T081 | Open | 2 | 2 | 100.00 |
| T09 | Other injuries of spine and trunk, level unspecified | 1631 | 1576 | 96.63 |
| T090 | Superficial injury of trunk, level unspecified | 155 | 155 | 100.00 |
| T091 | Open wound of trunk, level unspecified | 58 | 58 | 100.00 |
| T092 | Dislocation, sprain and strain of unspecified joint and ligament of trunk | 515 | 513 | 99.61 |
| T093 | Injury of spinal cord, level unspecified | 730 | 677 | 92.74 |
| T094 | Injury of unspecified nerve, spinal nerve root and plexus of trunk | 65 | 65 | 100.00 |
| T095 | Injury of unspecified muscle and tendon of trunk | 16 | 16 | 100.00 |
| T096 | Traumatic amputation of trunk, level unspecified | 0 | 0 | 0.00 |
| T098 | Other specified injuries of trunk, level unspecified | 4 | 4 | 100.00 |
| T099 | Unspecified injury of trunk, level unspecified | 86 | 86 | 100.00 |
| T10 | Fracture of upper limb, level unspecified | 22 | 22 | 100.00 |
| T100 | Closed | 16 | 16 | 100.00 |
| T101 | Open | 5 | 5 | 100.00 |
| T11 | Other injuries of upper limb, level unspecified | 353 | 348 | 98.58 |
| T110 | Superficial injury of upper limb, level unspecified | 111 | 109 | 98.20 |
| T111 | Open wound of upper limb, level unspecified | 111 | 109 | 98.20 |
| T112 | Dislocation, sprain and strain of unspecified joint and ligament of trunk joint and ligament of upper limb, level unspecified | 24 | 24 | 100.00 |
| T113 | Injury of unspecified nerve of upper limb, level unspecified | 2 | 2 | 100.00 |
| T114 | Injury of unspecified blood vessel of upper limb, level unspecified | 4 | 4 | 100.00 |
| T115 | Injury of unspecified muscle and tendon of upper limb, level unspecified | 30 | 30 | 100.00 |
| T116 | Traumatic amputation of upper limb, level unspecified | 16 | 15 | 93.75 |
| T118 | Other specified injuries of upper limb, level unspecified | 3 | 3 | 100.00 |
| T119 | Unspecified injury of upper limb, level unspecified | 52 | 52 | 100.00 |
| T12 | Fracture of lower limb, level unspecified | 70 | 68 | 97.14 |
| T120 | Closed | 52 | 52 | 100.00 |
| T121 | Open | 17 | 15 | 88.24 |
| T13 | Other injuries of lower limb, level unspecified | 672 | 660 | 98.21 |
| T130 | Superficial injury of lower limb, level unspecified | 157 | 157 | 100.00 |
| T131 | Open wound of lower limb, level unspecified | 254 | 248 | 97.64 |
| T132 | Dislocation, sprain and strain of unspecified joint and ligament of lower limb, level unspecified | 70 | 69 | 98.57 |
| T133 | Injury of unspecified nerve of lower limb, level unspecified | 6 | 6 | 100.00 |
| T134 | Injury of unspecified blood vessel of lower limb, level unspecified | 12 | 12 | 100.00 |
| T135 | Injury of unspecified muscle and tendon of lower limb, level unspecified | 72 | 72 | 100.00 |
| T136 | Traumatic amputation of lower limb, level unspecified | 14 | 9 | 64.29 |
| T138 | Other specified injuries of lower limb, level unspecified | 2 | 2 | 100.00 |
| T139 | Unspecified injury of lower limb, level unspecified | 85 | 85 | 100.00 |
| T14 | Injury of unspecified body region | 12382 | 12304 | 99.37 |
| T140 | Superficial injury of unspecified body region | 6113 | 6071 | 99.31 |
| T141 | Open wound of unspecified body region | 3706 | 3690 | 99.57 |
| T142 | Fracture of unspecified body region | 118 | 117 | 99.15 |
| T143 | Dislocation, sprain and strain of unspecified body region | 841 | 835 | 99.29 |
| T144 | Injury of nerve(s) of unspecified body region | 41 | 40 | 97.56 |
| T145 | Injury of blood vessel(s) of unspecified body region | 80 | 73 | 91.25 |
| T146 | Injury of muscles and tendons of unspecified body region | 1088 | 1084 | 99.63 |
| T147 | Crushing injury and traumatic amputation of unspecified body region | 283 | 282 | 99.65 |
| T148 | Other injuries of unspecified body region | 9 | 9 | 100.00 |
| T149 | Injury, unspecified | 101 | 101 | 100.00 |
| T15 | Foreign body on external eye | 396 | 396 | 100.00 |
| T150 | Foreign body in cornea | 212 | 212 | 100.00 |
| T151 | Foreign body in conjunctival sac | 77 | 77 | 100.00 |
| T158 | Foreign body in other and multiple parts of external eye | 21 | 21 | 100.00 |
| T159 | Foreign body on external eye, part unspecified | 86 | 86 | 100.00 |
| T16 | Foreign body in ear | 156 | 155 | 99.36 |
| T17 | Foreign body in respiratory tract | 1085 | 981 | 90.41 |
| T170 | Foreign body in nasal sinus | 12 | 12 | 100.00 |
| T171 | Foreign body in nostril | 57 | 57 | 100.00 |
| T172 | Foreign body in pharynx | 402 | 395 | 98.26 |
| T173 | Foreign body in larynx | 71 | 68 | 95.77 |
| T174 | Foreign body in trachea | 56 | 51 | 91.07 |
| T175 | Foreign body in bronchus | 193 | 187 | 96.89 |
| T178 | Foreign body in other and multiple parts of respiratory tract | 37 | 31 | 83.78 |
| T179 | Foreign body in respiratory tract, part unspecified | 255 | 178 | 69.80 |
| T18 | Foreign body in alimentary tract | 3838 | 3828 | 99.74 |
| T180 | Foreign body in mouth | 19 | 19 | 100.00 |
| T181 | Foreign body in esophagus | 2087 | 2080 | 99.66 |
| T182 | Foreign body in stomach | 480 | 480 | 100.00 |
| T183 | Foreign body in small intestine | 218 | 218 | 100.00 |
| T184 | Foreign body in colon | 91 | 91 | 100.00 |
| T185 | Foreign body in anus and rectum | 180 | 180 | 100.00 |
| T188 | Foreign body in other and multiple parts of alimentary tract | 25 | 25 | 100.00 |
| T189 | Foreign body in alimentary tract, part unspecified | 734 | 731 | 99.59 |
| T19 | Foreign body in genitourinary tract | 76 | 75 | 98.68 |
| T190 | Foreign body in urethra | 16 | 16 | 100.00 |
| T191 | Foreign body in bladder | 23 | 23 | 100.00 |
| T192 | Foreign body in vulva and vagina | 16 | 16 | 100.00 |
| T193 | Foreign body in uterus [any part] | 2 | 1 | 50.00 |
| T198 | Foreign body in other and multiple parts of genitourinary tract | 12 | 12 | 100.00 |
| T199 | Foreign body in genitourinary tract, part unspecified | 7 | 7 | 100.00 |
| T20 | Burn and corrosion of head and neck | 3409 | 3295 | 96.66 |
| T200 | Burn of unspecified degree of head and neck | 179 | 177 | 98.88 |
| T201 | Burn of first degree of head and neck | 142 | 141 | 99.30 |
| T202 | Burn of second degree of head and neck | 2916 | 2838 | 97.33 |
| T203 | Burn of third degree of head and neck | 137 | 107 | 78.10 |
| T204 | Corrosion of unspecified degree of head and neck | 10 | 10 | 100.00 |
| T205 | Corrosion of first degree of head and neck | 4 | 4 | 100.00 |
| T206 | Corrosion of second degree of head and neck | 13 | 10 | 76.92 |
| T207 | Corrosion of third degree of head and neck | 4 | 4 | 100.00 |
| T21 | Burn and corrosion of trunk | 2267 | 2146 | 94.66 |
| T210 | Burn of unspecified degree of trunk | 65 | 62 | 95.38 |
| T211 | Burn of first degree of trunk | 57 | 57 | 100.00 |
| T212 | Burn of second degree of trunk | 1797 | 1739 | 96.77 |
| T213 | Burn of third degree of trunk | 328 | 269 | 82.01 |
| T214 | Corrosion of unspecified degree of trunk | 1 | 1 | 100.00 |
| T215 | Corrosion of first degree of trunk | 4 | 4 | 100.00 |
| T216 | Corrosion of second degree of trunk | 8 | 7 | 87.50 |
| T217 | Corrosion of third degree of trunk | 1 | 1 | 100.00 |
| T22 | Burn and corrosion of shoulder and upper limb, except wrist and hand | 1884 | 1832 | 97.24 |
| T220 | Burn of unspecified degree of shoulder and upper limb, except wrist and hand | 78 | 75 | 96.15 |
| T221 | Burn of first degree of shoulder and upper limb, except wrist and hand | 36 | 36 | 100.00 |
| T222 | Burn of second degree of shoulder and upper limb, except wrist and hand | 1442 | 1429 | 99.10 |
| T223 | Burn of third degree of shoulder and upper limb, except wrist and hand | 313 | 279 | 89.14 |
| T224 | Corrosion of unspecified degree of shoulder and upper limb, except wrist and hand | 1 | 1 | 100.00 |
| T225 | Corrosion of first degree of shoulder and upper limb, except wrist and hand | 1 | 1 | 100.00 |
| T226 | Corrosion of second degree of shoulder and upper limb, except wrist and hand | 9 | 8 | 88.89 |
| T227 | Corrosion of third degree of shoulder and upper limb, except wrist and hand | 3 | 2 | 66.67 |
| T23 | Burn and corrosion of wrist and hand | 2688 | 2617 | 97.36 |
| T230 | Burn of unspecified degree of wrist and hand | 166 | 163 | 98.19 |
| T231 | Burn of first degree of wrist and hand | 74 | 73 | 98.65 |
| T232 | Burn of second degree of wrist and hand | 1994 | 1963 | 98.45 |
| T233 | Burn of third degree of wrist and hand | 431 | 395 | 91.65 |
| T234 | Corrosion of unspecified degree of wrist and hand | 6 | 6 | 100.00 |
| T235 | Corrosion of first degree of wrist and hand | 1 | 1 | 100.00 |
| T236 | Corrosion of second degree of wrist and hand | 10 | 10 | 100.00 |
| T237 | Corrosion of third degree of wrist and hand | 3 | 3 | 100.00 |
| T24 | Burn and corrosion of hip and lower limb, except ankle and foot | 3068 | 2999 | 97.75 |
| T240 | Burn of unspecified degree of hip and lower limb, except ankle and foot | 104 | 103 | 99.04 |
| T241 | Burn of first degree of hip and lower limb, except ankle and foot | 50 | 50 | 100.00 |
| T242 | Burn of second degree of hip and lower limb, except ankle and foot | 2410 | 2388 | 99.09 |
| T243 | Burn of third degree of hip and lower limb, except ankle and foot | 471 | 428 | 90.87 |
| T244 | Corrosion of unspecified degree of hip and lower limb, except ankle and foot | 6 | 5 | 83.33 |
| T245 | Corrosion of first degree of hip and lower imb, except ankle and foot | 2 | 2 | 100.00 |
| T246 | Corrosion of second degree of hip and lower limb, except ankle and foot | 17 | 15 | 88.24 |
| T247 | Corrosion of third degree of hip and lower limb, except ankle and foot | 5 | 5 | 100.00 |
| T25 | Burn and corrosion of ankle and foot | 1562 | 1522 | 97.44 |
| T250 | Burn of unspecified degree of ankle and foot | 56 | 54 | 96.43 |
| T251 | Burn of first degree of ankle and foot | 28 | 28 | 100.00 |
| T252 | Burn of second degree of ankle and foot | 1174 | 1159 | 98.72 |
| T253 | Burn of third degree of ankle and foot | 292 | 269 | 92.12 |
| T254 | Corrosion unspecified degree of ankle and foot | 2 | 2 | 100.00 |
| T255 | Corrosion of first degree of ankle and foot | 0 | 0 | 0.00 |
| T256 | Corrosion of second degree of ankle and foot | 7 | 7 | 100.00 |
| T257 | Corrosion of third degree of ankle and foot | 3 | 3 | 100.00 |
| T26 | Burn and corrosion confined to eye and adnexa | 307 | 304 | 99.02 |
| T260 | Burn of eyelid and periocular area | 87 | 85 | 97.70 |
| T261 | Burn of cornea and conjunctival sac | 124 | 123 | 99.19 |
| T262 | Burn with resulting rupture and destruction of eyeball | 0 | 0 | 0.00 |
| T263 | Burn of other parts of eye and adnexa | 10 | 10 | 100.00 |
| T264 | Burn of eye and adnexa, part unspecified | 21 | 21 | 100.00 |
| T265 | Corrosion of eyelid and periocular area | 5 | 5 | 100.00 |
| T266 | Corrosion of cornea and conjunctival sac | 40 | 40 | 100.00 |
| T267 | Corrosion with resulting rupture and destruction of eyeball | 11 | 11 | 100.00 |
| T268 | Corrosion of other parts of eye and adnexa | 4 | 4 | 100.00 |
| T269 | Corrosion of eye and adnexa, part unspecified | 5 | 5 | 100.00 |
| T27 | Burn and corrosion of respiratory tract | 403 | 356 | 88.34 |
| T270 | Burn of larynx and trachea | 50 | 46 | 92.00 |
| T271 | Burn involving larynx and trachea with lung | 64 | 51 | 79.69 |
| T272 | Burn of other parts of respiratory tract | 62 | 51 | 82.26 |
| T273 | Burn of respiratory tract, part unspecified | 205 | 187 | 91.22 |
| T274 | Corrosion of larynx and trachea | 10 | 10 | 100.00 |
| T275 | Corrosion involving larynx and trachea with lung | 0 | 0 | 0.00 |
| T276 | Corrosion of other parts of respiratory tract | 3 | 3 | 100.00 |
| T277 | Corrosion of respiratory tract, part unspecified | 6 | 5 | 83.33 |
| T28 | Burn and corrosion of other internal organs | 301 | 289 | 96.01 |
| T280 | Burn of mouth and pharynx | 20 | 20 | 100.00 |
| T281 | Burn of esophagus | 8 | 7 | 87.50 |
| T282 | Burn of other parts of alimentary tract | 0 | 0 | 0.00 |
| T283 | Burn of internal genitourinary organs | 6 | 6 | 100.00 |
| T284 | Burn of other and unspecified internal organs | 1 | 1 | 100.00 |
| T285 | Corrosion of mouth and pharynx | 21 | 20 | 95.24 |
| T286 | Corrosion of esophagus | 122 | 118 | 96.72 |
| T287 | Corrosion of other parts of alimentary tract | 119 | 113 | 94.96 |
| T288 | Corrosion of internal genitourinary organs | 1 | 1 | 100.00 |
| T289 | Corrosion of other and unspecified internal organs | 3 | 3 | 100.00 |
| T29 | Burns and corrosions of multiple body regions | 552 | 483 | 87.50 |
| T290 | Burns of multiple regions, unspecified degree | 59 | 54 | 91.53 |
| T291 | Burns of multiple regions, no more than first-degree burns mentioned | 3 | 3 | 100.00 |
| T292 | Burns of multiple regions, no more than second-degree burns mentioned | 254 | 243 | 95.67 |
| T293 | Burns of multiple regions, at least one burn of third degree mentioned | 229 | 176 | 76.86 |
| T294 | Corrosions of multiple regions, unspecified degree | 1 | 1 | 100.00 |
| T295 | Corrosions of multiple regions, no more than first-degree corrosions mentioned | 0 | 0 | 0.00 |
| T296 | Corrosions of multiple regions, no more than second-degree corrosions mentioned | 3 | 3 | 100.00 |
| T297 | Corrosions of multiple regions, at least one corrosion of third degree mentioned | 3 | 3 | 100.00 |
| T30 | Burn and corrosion, body region unspecified | 907 | 881 | 97.13 |
| T300 | Burn of unspecified body region, unspecified degree | 456 | 441 | 96.71 |
| T301 | Burn of first degree, body region unspecified | 18 | 18 | 100.00 |
| T302 | Burn of second degree, body region unspecified | 373 | 369 | 98.93 |
| T303 | Burn of third degree, body region unspecified | 30 | 24 | 80.00 |
| T304 | Corrosion of unspecified body region, unspecified degree | 30 | 29 | 96.67 |
| T305 | Corrosion of first degree, body region unspecified | 0 | 0 | 0.00 |
| T306 | Corrosion of second degree, body region unspecified | 0 | 0 | 0.00 |
| T307 | Corrosion of third degree, body region unspecified | 0 | 0 | 0.00 |
| T31 | Burns classified according to extent of body surface involved | 1932 | 1792 | 92.75 |
| T310 | Burns involving less than 10％ of body surface | 1087 | 1078 | 99.17 |
| T311 | Burns involving 10-19％ of body surface | 383 | 375 | 97.91 |
| T312 | Burns involving 20-29％ of body surface | 202 | 192 | 95.05 |
| T313 | Burns involving 30-39％ of body surface | 89 | 72 | 80.90 |
| T314 | Burns involving 40-49％ of body surface | 47 | 31 | 65.96 |
| T315 | Burns involving 50-59％ of body surface | 26 | 18 | 69.23 |
| T316 | Burns involving 60-69％ of body surface | 37 | 18 | 48.65 |
| T317 | Burns involving 70-79％ of body surface | 13 | 5 | 38.46 |
| T318 | Burns involving 80-89％ of body surface | 22 | 1 | 4.55 |
| T319 | Burns involving 90％ or more of body surface | 26 | 2 | 7.69 |
| T32 | Corrosions classified according to extent of body surface involved | 9 | 8 | 88.89 |
| T320 | Corrosions involving less than 10％ of body Corrosions involving 10-19％ of body surface | 8 | 7 | 87.50 |
| T321 | Corrosions involving 10-19％ of body surface | 1 | 1 | 100.00 |
| T322 | Corrosions involving 20-29％ of body surface | 0 | 0 | 0.00 |
| T323 | Corrosions involving 30-39％ of body surface | 0 | 0 | 0.00 |
| T324 | Corrosions involving 40-49％ of body surface | 0 | 0 | 0.00 |
| T325 | Corrosions involving 50-59％ of body surface | 0 | 0 | 0.00 |
| T326 | Corrosions involving 60-69％ of body surface | 0 | 0 | 0.00 |
| T327 | Corrosions involving 70-79％ of body surface | 0 | 0 | 0.00 |
| T328 | Corrosions involving 80-89％ of body surface | 0 | 0 | 0.00 |
| T329 | Corrosions involving 90％ or more of body surface | 0 | 0 | 0.00 |
| T33 | Superficial frostbite | 0 | 0 | excluded |
| T34 | Frostbite with tissue necrosis | 0 | 0 | excluded |
| T35 | Frostbite involving multiple body regions and unspecified frostbite | 0 | 0 | excluded |
| T36 | Poisoning by systemic antibiotics | 0 | 0 | excluded |
| T37 | Poisoning by other systemic anti-infectives and antiparasitic | 0 | 0 | excluded |
| T38 | Poisoning by hormones and their synthetic substitutes and antagonists, NEC | 0 | 0 | excluded |
| T39 | Poisoning by nonopioid analgesics, antipyretics and antirheumatics | 0 | 0 | excluded |
| T40 | Poisoning by narcotics and psychodysleptics [hallucinogens] | 0 | 0 | excluded |
| T41 | Poisoning by anesthetics and therapeutic gases | 0 | 0 | excluded |
| T42 | Poisoning by antiepileptic, sedative-hypnotic and antiparkinsonism drugs | 0 | 0 | excluded |
| T43 | Poisoning by psychotropic drugs, NEC | 0 | 0 | excluded |
| T44 | Poisoning by drugs primarily affecting the autonomic nervous system | 0 | 0 | excluded |
| T45 | Poisoning by primarily systemic and hematological agents, NEC | 0 | 0 | excluded |
| T46 | Poisoning by agents primarily affecting the cardiovascular system | 0 | 0 | excluded |
| T47 | Poisoning by agents primarily affecting the gastrointestinal system | 0 | 0 | excluded |
| T48 | Poisoning by agents primarily acting on smooth and skeletal muscles and the respiratory system | 0 | 0 | excluded |
| T49 | Poisoning by topical agents primarily affecting skin and mucous membrane and by ophthalmological, otorhinolaryngological and dental drugs | 0 | 0 | excluded |
| T50 | Poisoning by diuretics and other and unspecified drugs, medicaments and biological substances | 0 | 0 | excluded |
| T51 | Toxic effect of alcohol | 0 | 0 | excluded |
| T52 | Toxic effect of organic solvents | 0 | 0 | excluded |
| T53 | Toxic effect of halogen derivatives of aliphatic and aromatic hydrocarbons | 0 | 0 | excluded |
| T54 | Toxic effect of corrosive substances | 0 | 0 | excluded |
| T55 | Toxic effect of soaps and detergents | 0 | 0 | excluded |
| T56 | Toxic effect of metals | 0 | 0 | excluded |
| T57 | Toxic effect of other inorganic substances | 0 | 0 | excluded |
| T58 | Toxic effect of carbon monoxide | 0 | 0 | excluded |
| T59 | Toxic effect of other gases, fumes and vapours | 0 | 0 | excluded |
| T60 | Toxic effect of pesticides | 0 | 0 | excluded |
| T61 | Toxic effect of noxious substances eaten as seafood | 0 | 0 | excluded |
| T62 | Toxic effect of other noxious substances eaten as food | 0 | 0 | excluded |
| T63 | Toxic effect of contact with venomous animals | 0 | 0 | excluded |
| T64 | Toxic effect of aflatoxin and other mycotoxin food contaminants | 0 | 0 | excluded |
| T65 | Toxic effect of other and unspecified substances | 0 | 0 | excluded |
| T66 | Unspecified effects of radiation | 0 | 0 | excluded |
| T67 | Effects of heat and light | 0 | 0 | excluded |
| T68 | Hypothermia | 0 | 0 | excluded |
| T69 | Other effects of reduced temperature | 0 | 0 | excluded |
| T70 | Effects of air pressure and water pressure | 0 | 0 | excluded |
| T71 | Asphyxiation | 0 | 0 | excluded |
| T73 | Effects of other deprivation | 0 | 0 | excluded |
| T74 | Maltreatment syndromes | 0 | 0 | excluded |
| T75 | Effects of other external causes | 0 | 0 | excluded |
| T76 | Unspecified effects of external causes | 0 | 0 | excluded |
| T77 |  | 0 | 0 | excluded |
| T78 | Adverse effects, NEC | 0 | 0 | excluded |
| T79 | Certain early complications of trauma, NEC | 3702 | 3144 | 84.93 |
| T790 | Air embolism (traumatic) | 1 | 1 | 100.00 |
| T791 | Fat embolism (traumatic) | 44 | 29 | 65.91 |
| T792 | Traumatic secondary and recurrent hemorrhage | 13 | 9 | 69.23 |
| T793 | Post-traumatic wound infection, NEC | 474 | 466 | 98.31 |
| T794 | Traumatic shock | 789 | 412 | 52.22 |
| T795 | Traumatic anuria | 5 | 1 | 20.00 |
| T796 | Traumatic ischemia of muscle | 1357 | 1250 | 92.11 |
| T797 | Traumatic subcutaneous emphysema | 923 | 883 | 95.67 |
| T798 | Other early complications of trauma | 89 | 87 | 97.75 |
| T799 | Unspecified early complication of trauma | 5 | 4 | 80.00 |
| T80 | Complications following infusion, transfusion and therapeutic injection | 0 | 0 | excluded |
| T81 | Complications of procedures, NEC | 0 | 0 | excluded |
| T82 | Complications of cardiac and vascular prosthetic devices, implants and grafts | 0 | 0 | excluded |
| T83 | Complications of genitourinary prosthetic devices, implants and grafts | 0 | 0 | excluded |
| T84 | Complications of internal orthopedic prosthetic devices, implants and grafts | 0 | 0 | excluded |
| T85 | Complications of other internal prosthetic devices, implants and grafts | 0 | 0 | excluded |
| T86 | Failure and rejection of transplanted organs and tissues | 0 | 0 | excluded |
| T87 | Complications peculiar to reattachment and amputation | 0 | 0 | excluded |
| T88 | Other complications of surgical and medical care, NEC | 0 | 0 | excluded |
| T90 | Sequelae of injuries of head | 158 | 153 | 96.84 |
| T900 | Sequelae of superficial injury of head | 2 | 2 | 100.00 |
| T901 | Sequelae of open wound of head | 3 | 3 | 100.00 |
| T902 | Sequelae of fracture of skull and facial bones | 26 | 25 | 96.15 |
| T903 | Sequelae of injury of cranial nerves | 0 | 0 | 0.00 |
| T904 | Sequelae of injury of eye and orbit | 5 | 5 | 100.00 |
| T905 | Sequelae of intracranial injury | 102 | 99 | 97.06 |
| T908 | Sequelae of other specified injuries of head | 4 | 4 | 100.00 |
| T909 | Sequelae of unspecified injury of head | 14 | 13 | 92.86 |
| T91 | Sequelae of injuries of neck and trunk | 266 | 259 | 97.37 |
| T910 | Sequelae of superficial injury and open wound of neck and trunk | 2 | 2 | 100.00 |
| T911 | Sequelae of fracture of spine | 170 | 166 | 97.65 |
| T912 | Sequelae of other fracture of thorax and pelvis | 34 | 32 | 94.12 |
| T913 | Sequelae of injury of spinal cord | 51 | 50 | 98.04 |
| T914 | Sequelae of injury of intrathoracic organs | 1 | 1 | 100.00 |
| T915 | Sequelae of injury of intra-abdominal and pelvic organs | 5 | 5 | 100.00 |
| T918 | Sequelae of other specified injuries of neck and trunk | 2 | 2 | 100.00 |
| T919 | Sequelae of unspecified injury of neck and trunk | 1 | 1 | 100.00 |
| T92 | Sequelae of injuries of upper limb | 46 | 46 | 100.00 |
| T920 | Sequelae of open wound of upper limb | 4 | 4 | 100.00 |
| T921 | Sequelae of fracture of arm | 16 | 16 | 100.00 |
| T922 | Sequelae of fracture at wrist and hand level | 8 | 8 | 100.00 |
| T923 | Sequelae of dislocation, sprain and strain of upper limb | 3 | 3 | 100.00 |
| T924 | Sequelae of injury of nerve of upper limb | 2 | 2 | 100.00 |
| T925 | Sequelae of injury of muscle and tendon of upper limb | 0 | 0 | 0.00 |
| T926 | Sequelae of crushing injury and traumatic amputation of upper limb | 12 | 12 | 100.00 |
| T928 | Sequelae of other specified injuries of upper limb | 1 | 1 | 100.00 |
| T929 | Sequelae of unspecified injury of upper limb | 0 | 0 | 0.00 |
| T93 | Sequelae of injuries of lower limb | 62 | 61 | 98.39 |
| T930 | Sequelae of open wound of lower limb | 7 | 7 | 100.00 |
| T931 | Sequelae of fracture of femur | 20 | 19 | 95.00 |
| T932 | Sequelae of other fractures of lower limb | 18 | 18 | 100.00 |
| T933 | Sequelae of dislocation, sprain and strain of lower limb | 6 | 6 | 100.00 |
| T934 | Sequelae of injury of nerve of lower limb | 1 | 1 | 100.00 |
| T935 | Sequelae of injury of muscle and tendon of lower limb | 3 | 3 | 100.00 |
| T936 | Sequelae of crushing injury and traumatic amputation of lower limb | 4 | 4 | 100.00 |
| T938 | Sequelae of other specified injuries of lower limb | 1 | 1 | 100.00 |
| T939 | Sequelae of unspecified injury of lower limb | 2 | 2 | 100.00 |
| T94 | Sequelae of injuries involving multiple and unspecified body regions | 1 | 1 | 100.00 |
| T940 | Sequelae of injuries involving multiple body regions | 0 | 0 | 0.00 |
| T941 | Sequelae of injuries, not specified by body region | 1 | 1 | 100.00 |
| T95 | Sequelae of burns, corrosions and frostbite | 15 | 15 | 100.00 |
| T950 | Sequelae of burn, corrosion and frostbite of head and neck | 3 | 3 | 100.00 |
| T951 | Sequelae of burn, corrosion and frostbite of trunk | 1 | 1 | 100.00 |
| T952 | Sequelae of burn, corrosion and frostbite of upper limb | 2 | 2 | 100.00 |
| T953 | Sequelae of burn, corrosion and frostbite of lower limb | 4 | 4 | 100.00 |
| T954 | Sequelae of burn and corrosion classifiable only according to extent of body surface involved | 0 | 0 | 0.00 |
| T958 | Sequelae of other specified burn, corrosion and frostbite | 5 | 5 | 100.00 |
| T959 | Sequelae of unspecified burn, corrosion and frostbite | 0 | 0 | 0.00 |
| T96 | Sequelae of poisoning by drugs, medicaments and biological substances | 21 | 20 | 95.24 |
| T97 | Sequelae of toxic effects of substances chiefly nonmedicinal as to source | 14 | 13 | 92.86 |
| T98 | Sequelae of other and unspecified effects of external causes | 9 | 9 | 100.00 |
| T980 | Sequelae of effects of foreign body entering through natural orifice | 5 | 5 | 100.00 |
| T981 | Sequelae of other and unspecified effects of external causes | 2 | 2 | 100.00 |
| T982 | Sequelae of certain early complications of trauma | 1 | 1 | 100.00 |
| T983 | Sequelae of complications of surgical and medical care, NEC | 1 | 1 | 100.00 |
| **Exclusive SRR** | | | | |
| **Code** | **Name** | **Total (N)** | **Survivor**  **(N)** | **Exclusive**  **SRR (%)** |
| S00 | superficial injury of head | 753 | 752 | 99.87 |
| S001 | Contusion of eyelid and periocular area | 30 | 30 | 100.00 |
| S002 | Other superficial injuries of eyelid and periocular area | 8 | 8 | 100.00 |
| S003 | Superficial injury of nose | 16 | 16 | 100.00 |
| S004 | Superficial injury of ear | 10 | 10 | 100.00 |
| S005 | Superficial injury of lip and oral cavity | 13 | 13 | 100.00 |
| S007 | Multiple superficial injuries of head | 6 | 6 | 100.00 |
| S008 | Superficial injury of other parts of head | 351 | 351 | 100.00 |
| S009 | Superficial injury of head, part unspecified | 152 | 152 | 100.00 |
| S01 | open wound of head | 2642 | 2641 | 99.96 |
| S010 | Open wound of scalp | 600 | 599 | 99.83 |
| S011 | Open wound of eyelid and periocular area | 335 | 335 | 100.00 |
| S012 | Open wound of nose | 79 | 79 | 100.00 |
| S013 | Open wound of ear | 158 | 158 | 100.00 |
| S014 | Open wound of cheek and temporomandibular area | 161 | 161 | 100.00 |
| S015 | Open wound of lip | 297 | 297 | 100.00 |
| S017 | Multiple open wounds of head | 46 | 46 | 100.00 |
| S018 | Open wound of other parts of head | 875 | 875 | 100.00 |
| S019 | Open wound of head, part unspecified | 67 | 67 | 100.00 |
| S02 | fracture of skull and facial bones | 5103 | 5078 | 99.51 |
| S020 | Fracture of vault of skull | 449 | 445 | 99.11 |
| S021 | Fracture of base of skull | 358 | 355 | 99.16 |
| S022 | Fracture of nasal bones | 988 | 987 | 99.90 |
| S023 | Fracture of orbital floor | 536 | 536 | 100.00 |
| S024 | Fracture of malar and maxillary bones | 633 | 632 | 99.84 |
| S025 | Fracture of tooth | 6 | 6 | 100.00 |
| S026 | Fracture of mandible | 1209 | 1207 | 99.83 |
| S027 | Multiple fractures involving skull and facial bones | 25 | 25 | 100.00 |
| S028 | Fractures of other skull and facial bones | 468 | 463 | 98.93 |
| S029 | Fracture of skull and facial bones, part unspecified | 418 | 409 | 97.85 |
| S03 | Dislocation, sprain and strain of joints and ligaments of head | 10 | 10 | 100.00 |
| S030 | Dislocation of jaw | 3 | 3 | 100.00 |
| S031 | Dislocation of septal cartilage of nose | 0 | 0 | 0.00 |
| S032 | Dislocation of tooth | 6 | 6 | 100.00 |
| S033 | Dislocation of other and unspecified parts of head | 0 | 0 | 0.00 |
| S034 | Sprain and strain of jaw | 1 | 1 | 100.00 |
| S035 | Sprain and strain of joints and ligaments of other and unspecified parts of head | 0 | 0 | 0.00 |
| S04 | Injury of cranial nerve | 49 | 49 | 100.00 |
| S040 | Injury of optic nerve and pathways | 29 | 29 | 100.00 |
| S041 | Injury of oculomotor nerve | 1 | 1 | 100.00 |
| S042 | Injury of trochlear nerve | 1 | 1 | 100.00 |
| S043 | Injury of trigeminal nerve | 2 | 2 | 100.00 |
| S044 | Injury of abducent nerve | 0 | 0 | 0.00 |
| S045 | Injury of facial nerve | 15 | 15 | 100.00 |
| S046 | Injury of acoustic nerve | 1 | 1 | 100.00 |
| S047 | Injury of accessory nerve | 0 | 0 | 0.00 |
| S048 | Injury of other cranial nerves | 0 | 0 | 0.00 |
| S049 | Injury of unspecified cranial nerve | 0 | 0 | 0.00 |
| S05 | Injury of eye and orbit | 1583 | 1583 | 100.00 |
| S050 | Injury of conjunctiva and corneal abrasion without mention of foreign body | 28 | 28 | 100.00 |
| S051 | Contusion of eyeball and orbital tissues | 344 | 344 | 100.00 |
| S052 | Ocular laceration and rupture with prolapse or loss of intraocular tissue | 104 | 104 | 100.00 |
| S053 | Ocular laceration without prolapse or loss of intraocular tissue | 408 | 408 | 100.00 |
| S054 | Penetrating wound of orbit with or without foreign body | 23 | 23 | 100.00 |
| S055 | Penetrating wound of eyeball with foreign body | 162 | 162 | 100.00 |
| S056 | Penetrating wound of eyeball without foreign body | 83 | 83 | 100.00 |
| S057 | Avulsion of eye | 2 | 2 | 100.00 |
| S058 | Other injuries of eye and orbit | 359 | 359 | 100.00 |
| S059 | Injury of eye and orbit, unspecified | 66 | 66 | 100.00 |
| S06 | Intracranial injury | 16275 | 14995 | 92.14 |
| S060 | Concussion | 3893 | 3889 | 99.90 |
| S061 | Traumatic cerebral oedema | 11 | 4 | 36.36 |
| S062 | Diffuse brain injury | 671 | 629 | 93.74 |
| S063 | Focal brain injury | 555 | 536 | 96.58 |
| S064 | Epidural haemorrhage | 1140 | 1106 | 97.02 |
| S065 | Traumatic subdural haemorrhage | 7653 | 6682 | 87.31 |
| S066 | Traumatic subarachnoid haemorrhage | 1802 | 1653 | 91.73 |
| S067 | Intracranial injury with prolonged coma | 0 | 0 | 0.00 |
| S068 | Other intracranial injuries | 474 | 436 | 91.98 |
| S069 | Intracranial injury, unspecified | 33 | 21 | 63.64 |
| S07 | Crushing injury of head | 14 | 14 | 100.00 |
| S070 | Crushing injury of face | 14 | 14 | 100.00 |
| S071 | Crushing injury of skull | 0 | 0 | 0.00 |
| S078 | Crushing injury of other parts of head | 0 | 0 | 0.00 |
| S079 | Crushing injury of head, part unspecified | 0 | 0 | 0.00 |
| S08 | Traumatic amputation of part of head | 31 | 31 | 100.00 |
| S080 | Avulsion of scalp | 9 | 9 | 100.00 |
| S081 | Traumatic amputation of ear | 18 | 18 | 100.00 |
| S088 | Traumatic amputation of other parts of head | 4 | 4 | 100.00 |
| S089 | Traumatic amputation of unspecified part of head | 0 | 0 | 0.00 |
| S09 | Other and unspecified injuries of head | 122 | 119 | 97.54 |
| S090 | Injury of blood vessels of head, NEC | 2 | 2 | 100.00 |
| S091 | Injury of muscle and tendon of head | 19 | 19 | 100.00 |
| S092 | Traumatic rupture of ear drum | 6 | 6 | 100.00 |
| S097 | Multiple injuries of head | 1 | 1 | 100.00 |
| S098 | Other specified injuries of head | 22 | 22 | 100.00 |
| S099 | Unspecified injury of head | 72 | 69 | 95.83 |
| S10 | Superficial injury of neck | 106 | 106 | 100.00 |
| S100 | Contusion of throat | 14 | 14 | 100.00 |
| S101 | Other and unspecified superficial injuries of throat | 10 | 10 | 100.00 |
| S107 | Multiple superficial injuries of neck | 3 | 3 | 100.00 |
| S108 | Superficial injury of other parts of neck | 34 | 34 | 100.00 |
| S109 | Superficial injury of neck, part unspecified | 45 | 45 | 100.00 |
| S11 | Open wound of neck | 245 | 243 | 99.18 |
| S111 | Open wound involving thyroid gland | 0 | 0 | 0.00 |
| S112 | Open wound involving pharynx and cervical esophagus | 6 | 6 | 100.00 |
| S117 | Multiple open wounds of neck | 14 | 14 | 100.00 |
| S118 | Open wound of other parts of neck | 65 | 65 | 100.00 |
| S119 | Open wound of neck, part unspecified | 149 | 147 | 98.66 |
| S12 | Fracture of neck | 925 | 912 | 98.59 |
| S120 | Fracture of first cervical vertebra | 42 | 41 | 97.62 |
| S121 | Fracture of second cervical vertebra | 194 | 192 | 98.97 |
| S122 | Fracture of other specified cervical vertebra | 212 | 210 | 99.06 |
| S127 | Multiple fractures of cervical spine | 73 | 70 | 95.89 |
| S128 | Fracture of other parts of neck | 36 | 36 | 100.00 |
| S129 | Fracture of neck, part unspecified | 366 | 361 | 98.63 |
| S13 | Dislocation, sprain and strain of joints and ligaments at neck level | 4891 | 4882 | 99.82 |
| S130 | Traumatic rupture of cervical intervertebra disc | 33 | 32 | 96.97 |
| S131 | Dislocation of cervical vertebra | 102 | 95 | 93.14 |
| S132 | Dislocation of other and unspecified parts of neck | 2 | 2 | 100.00 |
| S133 | Multiple dislocations of neck | 2 | 2 | 100.00 |
| S134 | Sprain and strain of cervical spine | 4613 | 4612 | 99.98 |
| S135 | Sprain and strain of thyroid region | 2 | 2 | 100.00 |
| S136 | Sprain and strain of joints and ligaments of other and unspecified parts of neck | 132 | 132 | 100.00 |
| S14 | Injury of nerves and spinal cord at neck level | 383 | 375 | 97.91 |
| S140 | Concussion and oedema of cervical spinal cord | 49 | 49 | 100.00 |
| S141 | Other and unspecified injuries of cervical spinal cord | 296 | 288 | 97.30 |
| S142 | Injury of nerve root of cervical spine | 7 | 7 | 100.00 |
| S143 | Injury of brachial plexus | 16 | 16 | 100.00 |
| S144 | Injury of peripheral nerves of neck | 0 | 0 | 0.00 |
| S145 | Injury of cervical sympathetic nerves | 0 | 0 | 0.00 |
| S146 | Injury of other and unspecified nerves of neck | 9 | 9 | 100.00 |
| S15 | Injury of blood vessels at neck level | 48 | 48 | 100.00 |
| S150 | Injury of carotid artery | 5 | 5 | 100.00 |
| S151 | Injury of vertebral artery | 1 | 1 | 100.00 |
| S152 | Injury of external jugular vein | 4 | 4 | 100.00 |
| S153 | Injury of internal jugular vein | 2 | 2 | 100.00 |
| S157 | Injury of multiple blood vessels at neck level | 0 | 0 | 0.00 |
| S158 | Injury of other blood vessels at neck level | 21 | 21 | 100.00 |
| S159 | Injury of unspecified blood vessels at neck level | 15 | 15 | 100.00 |
| S16 | Injury of muscle and tendon at neck level | 9 | 9 | 100.00 |
| S17 | Crushing injury of neck | 1 | 1 | 100.00 |
| S170 | Crushing injury of larynx and trachea | 0 | 0 | 0.00 |
| S178 | Crushing injury of other parts of neck | 0 | 0 | 0.00 |
| S179 | Crushing injury of neck, part unspecified | 1 | 1 | 100.00 |
| S18 | Traumatic amputation at neck level | 0 | 0 | 0.00 |
| S19 | Other and unspecified injuries of neck | 37 | 37 | 100.00 |
| S197 | Multiple injuries of neck | 1 | 1 | 100.00 |
| S198 | Other specified injuries of neck | 16 | 16 | 100.00 |
| S199 | Unspecified injury of neck | 20 | 20 | 100.00 |
| S20 | Superficial injury of thorax | 1670 | 1668 | 99.88 |
| S200 | Contusion of breast | 3 | 3 | 100.00 |
| S201 | Other and unspecified superficial injuries of breast | 2 | 2 | 100.00 |
| S202 | Contusion of thorax | 1628 | 1627 | 99.94 |
| S203 | Other superficial injuries of front wall of thorax | 8 | 8 | 100.00 |
| S204 | Other superficial injuries of back wall of thorax | 1 | 1 | 100.00 |
| S207 | Multiple superficial injuries of thorax | 0 | 0 | 0.00 |
| S208 | Superficial injury of other and unspecified parts of thorax | 28 | 27 | 96.43 |
| S21 | Open wound of thorax | 216 | 216 | 100.00 |
| S210 | Open wound of breast | 3 | 3 | 100.00 |
| S211 | Open wound of front wall of thorax | 76 | 76 | 100.00 |
| S212 | Open wound of back wall of thorax | 55 | 55 | 100.00 |
| S217 | Multiple open wounds of thoracic wall | 3 | 3 | 100.00 |
| S218 | Open wound of other parts of thorax | 16 | 16 | 100.00 |
| S219 | Open wound of thorax, part unspecified | 63 | 63 | 100.00 |
| S22 | Fracture of rib(s), sternum and thoracic spine | 12208 | 12178 | 99.75 |
| S220 | Fracture of thoracic vertebra | 4152 | 4140 | 99.71 |
| S221 | Multiple fractures of thoracic spine | 91 | 91 | 100.00 |
| S222 | Fracture of sternum | 972 | 969 | 99.69 |
| S223 | Fracture of rib | 1590 | 1585 | 99.69 |
| S224 | Multiple fracture of ribs | 5351 | 5341 | 99.81 |
| S225 | Flail chest | 13 | 13 | 100.00 |
| S228 | Fracture of other parts of bony thorax | 11 | 11 | 100.00 |
| S229 | Fracture of bony thorax, part unspecified | 7 | 7 | 100.00 |
| S23 | Dislocation, sprain and strain of joints and ligaments of thorax | 219 | 219 | 100.00 |
| S230 | Traumatic rupture of thoracic intervertebral disc | 0 | 0 | 0.00 |
| S231 | Dislocation of thoracic vertebra | 5 | 5 | 100.00 |
| S232 | Dislocation of other and unspecified parts of thorax | 2 | 2 | 100.00 |
| S233 | Sprain and strain of thoracic spine | 79 | 79 | 100.00 |
| S234 | Sprain and strain of ribs and sternum | 105 | 105 | 100.00 |
| S235 | Sprain and strain of other and unspecified parts of thorax | 27 | 27 | 100.00 |
| S24 | Injury of nerves and spinal cord at thorax level | 14 | 13 | 92.86 |
| S240 | Concussion and oedema of thoracic spinal cord | 2 | 2 | 100.00 |
| S241 | Other and unspecified injuries of thoracic spinal cord | 9 | 8 | 88.89 |
| S242 | Injury of nerve root of thoracic spine | 1 | 1 | 100.00 |
| S243 | Injury of peripheral nerves of thorax | 0 | 0 | 0.00 |
| S244 | Injury of thoracic sympathetic nerves | 1 | 1 | 100.00 |
| S245 | Injury of other nerves of thorax | 0 | 0 | 0.00 |
| S246 | Injury of unspecified nerve of thorax | 1 | 1 | 100.00 |
| S25 | Injury of blood vessels of thorax | 27 | 20 | 74.07 |
| S250 | Injury of thoracic aorta | 16 | 9 | 56.25 |
| S251 | Injury of innominate or subclavian artery | 0 | 0 | 0.00 |
| S252 | Injury of superior vena cava | 0 | 0 | 0.00 |
| S253 | Injury of innominate or subclavian vein | 2 | 2 | 100.00 |
| S254 | Injury of pulmonary blood vessels | 0 | 0 | 0.00 |
| S255 | Injury of intercostal blood vessels | 0 | 0 | 0.00 |
| S257 | Injury of multiple blood vessels of thorax | 0 | 0 | 0.00 |
| S258 | Injury of other blood vessels of thorax | 5 | 5 | 100.00 |
| S259 | Injury of unspecified blood vessel of thorax | 4 | 4 | 100.00 |
| S26 | Injury of heart | 26 | 20 | 76.92 |
| S260 | Injury of heart with hemopericardium | 15 | 12 | 80.00 |
| S268 | Other injuries of heart | 5 | 5 | 100.00 |
| S269 | Injury of heart, unspecified | 6 | 3 | 50.00 |
| S27 | Injury of other and unspecified intrathoracic organs | 937 | 914 | 97.55 |
| S270 | Traumatic pneumothorax | 399 | 395 | 99.00 |
| S271 | Traumatic hemothorax | 202 | 194 | 96.04 |
| S272 | Traumatic hemopneumothorax | 189 | 182 | 96.30 |
| S273 | Other injuries of lung | 76 | 75 | 98.68 |
| S274 | Injury of bronchus | 2 | 2 | 100.00 |
| S275 | Injury of thoracic trachea | 3 | 3 | 100.00 |
| S276 | Injury of pleura | 3 | 3 | 100.00 |
| S277 | Multiple injuries of intrathoracic organs | 0 | 0 | 0.00 |
| S278 | Injury of other specified intrathoracic organs | 56 | 53 | 94.64 |
| S279 | Injury of unspecified intrathoracic organ | 4 | 4 | 100.00 |
| S28 | Crushing injury of thorax and traumatic amputation of part of thorax | 0 | 0 | 0.00 |
| S280 | Crushed chest | 0 | 0 | 0.00 |
| S281 | Traumatic amputation of part of thorax | 0 | 0 | 0.00 |
| S29 | Other and unspecified injuries of thorax | 15 | 15 | 100.00 |
| S290 | Injury of muscle and tendon at thorax level | 7 | 7 | 100.00 |
| S297 | Multiple injuries of thorax | 0 | 0 | 0.00 |
| S298 | Other specified injuries of thorax | 3 | 3 | 100.00 |
| S299 | Unspecified injury of thorax | 5 | 5 | 100.00 |
| S30 | Superficial injury of abdomen, lower back and pelvis | 1715 | 1713 | 99.88 |
| S300 | Contusion of lower back and pelvis | 696 | 695 | 99.86 |
| S301 | Contusion of abdominal wall | 602 | 601 | 99.83 |
| S302 | Contusion of external genital organs | 299 | 299 | 100.00 |
| S307 | Multiple superficial injuries of abdomen, lower back and pelvis | 3 | 3 | 100.00 |
| S308 | Other superficial injuries of abdomen, lower back and pelvis | 91 | 91 | 100.00 |
| S309 | Superficial injury of abdomen, lower back and pelvis, part unspecified | 23 | 23 | 100.00 |
| S31 | Open wound of abdomen, lower back and pelvis | 1161 | 1159 | 99.83 |
| S32 | Fracture of lumbar spine and pelvis | 11385 | 11354 | 99.73 |
| S320 | Fracture of lumbar vertebra | 8219 | 8211 | 99.90 |
| S321 | Fracture of sacrum | 365 | 364 | 99.73 |
| S322 | Fracture of coccyx | 187 | 187 | 100.00 |
| S323 | Fracture of ilium | 225 | 222 | 98.67 |
| S324 | Fracture of acetabulum | 501 | 500 | 99.80 |
| S325 | Fracture of pubis | 621 | 619 | 99.68 |
| S327 | Multiple fractures of lumbar spine and pelvis | 149 | 149 | 100.00 |
| S328 | Fracture of other and unspecified parts of lumbar spine and pelvis | 1079 | 1063 | 98.52 |
| S33 | Dislocation, sprain and strain of joints and ligaments of lumbar spine and pelvis | 6489 | 6486 | 99.95 |
| S330 | Traumatic rupture of lumbar intervertebral disc | 86 | 86 | 100.00 |
| S331 | Dislocation of lumbar vertebra | 63 | 62 | 98.41 |
| S332 | Dislocation of sacroiliac and sacrococcygeal joint | 3 | 3 | 100.00 |
| S333 | Dislocation of other and unspecified parts of lumbar spine and pelvis | 7 | 7 | 100.00 |
| S334 | Traumatic rupture of symphysis pubis | 4 | 4 | 100.00 |
| S335 | Sprain and strain of lumbar spine | 6042 | 6040 | 99.97 |
| S336 | Sprain and strain of sacroiliac joint | 8 | 8 | 100.00 |
| S337 | Sprain and strain of other and unspecified parts of lumbar spine and pelvis | 274 | 274 | 100.00 |
| S34 | Injury of nerves and lumbar spinal cord at abdomen, lower back and pelvis level | 20 | 20 | 100.00 |
| S340 | Concussion and oedema of lumbar spinal cord | 0 | 0 | 0.00 |
| S341 | Other injury of lumbar spinal cord | 18 | 18 | 100.00 |
| S342 | Injury of nerve root of lumbar and sacral spine | 1 | 1 | 100.00 |
| S343 | Injury of cauda equina | 0 | 0 | 0.00 |
| S344 | Injury of lumbosacral plexus | 1 | 1 | 100.00 |
| S345 | Injury of lumbar, sacral and pelvic sympathetic nerves | 0 | 0 | 0.00 |
| S346 | Injury of peripheral nerve(s) of abdomen, lower back and pelvis | 0 | 0 | 0.00 |
| S348 | Injury of other and unspecified nerves at abdomen, lower back and pelvis level | 0 | 0 | 0.00 |
| S35 | Injury of blood vessels at abdomen, lower back and pelvis level | 137 | 124 | 90.51 |
| S350 | Injury of abdominal aorta | 8 | 5 | 62.50 |
| S351 | Injury of inferior vena cava | 6 | 6 | 100.00 |
| S352 | Injury of coeliac or mesenteric artery | 44 | 42 | 95.45 |
| S353 | Injury of portal or splenic vein | 8 | 8 | 100.00 |
| S354 | Injury of renal blood vessels | 9 | 9 | 100.00 |
| S355 | Injury of iliac blood vessels | 12 | 12 | 100.00 |
| S357 | Injury of multiple blood vessels at abdomen, lower back and pelvis level | 2 | 0 | 0.00 |
| S358 | Injury of other blood vessels at abdomen, lower back and pelvis level | 29 | 26 | 89.66 |
| S359 | Injury of unspecified blood vessel at abdomen, lower back and pelvis level | 18 | 15 | 83.33 |
| S36 | Injury of intra-abdominal organs | 1650 | 1591 | 96.42 |
| S360 | Injury of spleen | 445 | 436 | 97.98 |
| S361 | Injury of liver or gallbladder | 551 | 530 | 96.19 |
| S362 | Injury of pancreas | 69 | 66 | 95.65 |
| S363 | Injury of stomach | 14 | 14 | 100.00 |
| S364 | Injury of small intestine | 146 | 140 | 95.89 |
| S365 | Injury of colon | 35 | 35 | 100.00 |
| S366 | Injury of rectum | 37 | 37 | 100.00 |
| S367 | Injury of multiple intra-abdominal organs | 3 | 3 | 100.00 |
| S368 | Injury of other intra-abdominal organs | 292 | 274 | 93.84 |
| S369 | Injury of unspecified intra-abdominal organ | 52 | 50 | 96.15 |
| S37 | Injury of urinary and pelvic organs | 662 | 658 | 99.40 |
| S370 | Injury of kidney | 338 | 335 | 99.11 |
| S371 | Injury of ureter | 9 | 9 | 100.00 |
| S372 | Injury of bladder | 54 | 54 | 100.00 |
| S373 | Injury of urethra | 220 | 220 | 100.00 |
| S374 | Injury of ovary | 0 | 0 | 0.00 |
| S375 | Injury of fallopian tube | 0 | 0 | 0.00 |
| S376 | Injury of uterus | 2 | 2 | 100.00 |
| S377 | Injury of multiple pelvic organs | 2 | 2 | 100.00 |
| S378 | Injury of other pelvic organs | 29 | 29 | 100.00 |
| S379 | Injury of unspecified pelvic organ | 3 | 2 | 66.67 |
| S38 | Crushing injury and traumatic amputation of part of abdomen, lower back and pelvis | 21 | 21 | 100.00 |
| S380 | Crushing injury of external genital organs | 7 | 7 | 100.00 |
| S381 | Crushing injury of other and unspecified parts of abdomen, lower back and pelvis | 2 | 2 | 100.00 |
| S382 | Traumatic amputation of external genital organs | 12 | 12 | 100.00 |
| S383 | Traumatic amputation of other and unspecified parts of abdomen, lower back and pelvis | 0 | 0 | 0.00 |
| S39 | Other and unspecified injuries of abdomen, lower back and pelvis | 427 | 424 | 99.30 |
| S390 | Injury of muscle and tendon of abdomen, lower back and pelvis | 26 | 25 | 96.15 |
| S396 | Injury of intra-abdominal organs(s) with pelvic organ(s) | 0 | 0 | 0.00 |
| S397 | Other multiple injuries of abdomen, lower back and pelvis | 3 | 2 | 66.67 |
| S398 | Other specified injuries of abdomen, lower back and pelvis | 78 | 78 | 100.00 |
| S399 | Unspecified injury of abdomen, lower back and pelvis | 320 | 319 | 99.69 |
| S40 | Superficial injury of shoulder and upper arm | 404 | 404 | 100.00 |
| S400 | Contusion of shoulder and upper arm | 397 | 397 | 100.00 |
| S407 | Multiple superficial injuries of shoulder and upper arm | 0 | 0 | 0.00 |
| S408 | Other superficial injuries of shoulder and upper arm | 6 | 6 | 100.00 |
| S409 | Superficial injury of shoulder and upper arm, unspecified | 1 | 1 | 100.00 |
| S41 | Open wound of shoulder and upper arm | 140 | 140 | 100.00 |
| S410 | Open wound of shoulder | 28 | 28 | 100.00 |
| S411 | Open wound of upper arm | 92 | 92 | 100.00 |
| S417 | Multiple open wounds of shoulder and upper arm | 9 | 9 | 100.00 |
| S418 | Open wound of other and unspecified parts of shoulder girdle | 11 | 11 | 100.00 |
| S42 | Fracture of shoulder and upper arm | 15333 | 15324 | 99.94 |
| S420 | Fracture of clavicle | 4464 | 4462 | 99.96 |
| S421 | Fracture of scapula | 238 | 238 | 100.00 |
| S422 | Fracture of upper end of humerus | 3006 | 3003 | 99.90 |
| S423 | Fracture of shaft of humerus | 2363 | 2362 | 99.96 |
| S424 | Fracture of lower end of humerus | 5192 | 5189 | 99.94 |
| S427 | Multiple fractures of clavicle, scapula and humerus | 1 | 1 | 100.00 |
| S428 | Fracture of other parts of shoulder and upper arm | 13 | 13 | 100.00 |
| S429 | Fracture of shoulder girdle, part unspecified | 25 | 25 | 100.00 |
| S43 | Dislocation, sprain and strain of joints and ligaments of shoulder girdle | 909 | 909 | 100.00 |
| S430 | Dislocation of shoulder joint | 299 | 299 | 100.00 |
| S431 | Anterior dislocation of humerus | 233 | 233 | 100.00 |
| S432 | Dislocation of sternoclavicular joint | 7 | 7 | 100.00 |
| S433 | Dislocation of other and unspecified parts of shoulder girdle | 25 | 25 | 100.00 |
| S434 | Sprain and strain of shoulder joint | 215 | 215 | 100.00 |
| S435 | Sprain and strain of acromioclavicular joint | 84 | 84 | 100.00 |
| S436 | Sprain and strain of sternoclavicular joint | 1 | 1 | 100.00 |
| S437 | Sprain and strain of other and unspecified | 40 | 40 | 100.00 |
| S439 | parts of shoulder girdle | 0 | 0 | 0.00 |
| S44 | Injury of nerves at shoulder and upper arm level | 9 | 9 | 100.00 |
| S440 | Injury of ulnar nerve at upper arm level | 3 | 3 | 100.00 |
| S441 | Injury of median nerve at upper arm level | 0 | 0 | 0.00 |
| S442 | Injury of radial nerve at upper arm level | 4 | 4 | 100.00 |
| S443 | Injury of axillary nerve | 1 | 1 | 100.00 |
| S444 | Injury of musculocutaneous nerve | 0 | 0 | 0.00 |
| S445 | Injury of cutaneous sensory nerve at shoulder and upper arm level | 0 | 0 | 0.00 |
| S447 | Injury of multiple nerves at shoulder and upper arm level | 0 | 0 | 0.00 |
| S448 | Injury of other nerves at shoulder and upper arm level | 0 | 0 | 0.00 |
| S449 | Injury of unspecified nerve at shoulder and upper arm level | 0 | 0 | 0.00 |
| S45 | Injury of blood vessels at shoulder and upper arm level | 16 | 16 | 100.00 |
| S450 | Injury of axillary artery | 0 | 0 | 0.00 |
| S451 | Injury of brachial artery | 7 | 7 | 100.00 |
| S452 | Injury of axillary or brachial vein | 1 | 1 | 100.00 |
| S453 | Injury of superficial vein at shoulder and upper arm level | 2 | 2 | 100.00 |
| S457 | Injury of multiple blood vessels at shoulder and upper arm level | 0 | 0 | 0.00 |
| S458 | Injury of other blood vessels at shoulder and upper arm level | 6 | 6 | 100.00 |
| S459 | Injury of unspecified blood vessel at shoulder and upper arm level | 0 | 0 | 0.00 |
| S46 | Injury of muscle and tendon at shoulder and upper arm level | 197 | 197 | 100.00 |
| S460 | Injury of muscle(s) and tendon(s) of the rotator cuff of shoulder | 58 | 58 | 100.00 |
| S461 | Injury of muscle and tendon of long head of biceps | 5 | 5 | 100.00 |
| S462 | Injury of muscle and tendon of other parts of biceps | 20 | 20 | 100.00 |
| S463 | Injury of muscle and tendon of triceps | 35 | 35 | 100.00 |
| S467 | Injury of multiple muscles and tendons at shoulder and upper arm level | 15 | 15 | 100.00 |
| S468 | Injury of other muscles and tendon at shoulder and upper arm level | 39 | 39 | 100.00 |
| S469 | Injury of unspecified muscle and tendon at shoulder and upper arm level | 25 | 25 | 100.00 |
| S47 | Crushing injury of shoulder and upper arm | 5 | 5 | 100.00 |
| S48 | Traumatic amputation of shoulder and upper arm | 5 | 5 | 100.00 |
| S480 | Traumatic amputation at shoulder joint | 1 | 1 | 100.00 |
| S481 | Traumatic amputation at level between shoulder and elbow | 4 | 4 | 100.00 |
| S489 | Traumatic amputation of shoulder and upper arm, level unspecified | 0 | 0 | 0.00 |
| S49 | Other and unspecified injuries of shoulder and upper arm | 5 | 5 | 100.00 |
| S497 | Multiple injuries of shoulder and upper arm | 0 | 0 | 0.00 |
| S498 | Other specified injuries of shoulder and upper arm | 1 | 1 | 100.00 |
| S499 | Unspecified injury of shoulder and upper arm | 4 | 4 | 100.00 |
| S50 | Superficial injury of forearm | 234 | 234 | 100.00 |
| S500 | Contusion of elbow | 135 | 135 | 100.00 |
| S501 | Contusion of other and unspecified parts of forearm | 68 | 68 | 100.00 |
| S507 | Multiple superficial injuries of forearm | 0 | 0 | 0.00 |
| S508 | Other superficial injuries of forearm | 28 | 28 | 100.00 |
| S509 | Superficial injury of forearm, unspecified | 3 | 3 | 100.00 |
| S51 | Open wound of forearm | 784 | 784 | 100.00 |
| S510 | Open wound of elbow | 130 | 130 | 100.00 |
| S517 | Multiple open wounds of forearm | 54 | 54 | 100.00 |
| S518 | Open wound of other parts of forearm | 212 | 212 | 100.00 |
| S519 | Open wound of forearm, part unspecified | 385 | 385 | 100.00 |
| S52 | Fracture of forearm | 13341 | 13338 | 99.98 |
| S520 | Fracture of upper end of ulna | 1179 | 1179 | 100.00 |
| S521 | Fracture of upper end of radius | 708 | 708 | 100.00 |
| S522 | Fracture of shaft of ulna | 384 | 384 | 100.00 |
| S523 | Fracture of shaft of radius | 432 | 432 | 100.00 |
| S524 | Fracture of shafts of both ulna and radius | 1005 | 1005 | 100.00 |
| S525 | Fracture of lower end of radius | 6219 | 6217 | 99.97 |
| S526 | Fracture of lower end of both ulna and radius | 2879 | 2879 | 100.00 |
| S527 | Multiple fracture of forearm | 121 | 121 | 100.00 |
| S528 | Fracture of other parts of forearm | 210 | 210 | 100.00 |
| S529 | Fracture of forearm, part unspecified | 195 | 194 | 99.49 |
| S53 | Dislocation, sprain and strain of joints and ligaments of elbow | 280 | 280 | 100.00 |
| S530 | Dislocation of radial head | 4 | 4 | 100.00 |
| S531 | Dislocation of elbow, unspecified | 212 | 212 | 100.00 |
| S532 | Traumatic rupture of radial collateral ligament | 7 | 7 | 100.00 |
| S533 | Traumatic rupture of ulnar collateral ligament | 13 | 13 | 100.00 |
| S534 | Sprain and strain of elbow | 43 | 43 | 100.00 |
| S54 | Injury of nerves at forearm level | 22 | 22 | 100.00 |
| S540 | Injury of ulnar nerve at forearm level | 7 | 7 | 100.00 |
| S541 | Injury of median nerve at forearm level | 3 | 3 | 100.00 |
| S542 | Injury of radial nerve at forearm level | 8 | 8 | 100.00 |
| S543 | Injury of cutaneous sensory nerve at forearm level | 2 | 2 | 100.00 |
| S547 | Injury of multiple nerves at forearm level | 0 | 0 | 0.00 |
| S548 | Injury of other nerves at forearm level | 2 | 2 | 100.00 |
| S549 | Injury of unspecified nerve at forearm level | 0 | 0 | 0.00 |
| S55 | Injury of blood vessels at forearm level | 51 | 51 | 100.00 |
| S550 | Injury of ulnar artery at forearm level | 8 | 8 | 100.00 |
| S551 | Injury of radial artery at forearm level | 24 | 24 | 100.00 |
| S552 | Injury of vein at forearm level | 4 | 4 | 100.00 |
| S557 | Injury of multiple blood vessels at forearm level | 0 | 0 | 0.00 |
| S558 | Injury of other blood vessels at forearm level | 13 | 13 | 100.00 |
| S559 | Injury of unspecified blood vessels at forearm level | 2 | 2 | 100.00 |
| S56 | Injury of muscle and tendon at forearm level | 1062 | 1061 | 99.91 |
| S560 | Injury of flexor muscle and tendon of thumb at forearm level | 29 | 29 | 100.00 |
| S561 | Injury of flexor muscle and tendon of other finger(s) at forearm level | 133 | 133 | 100.00 |
| S562 | Injury of other flexor muscle and tendon at forearm level | 181 | 181 | 100.00 |
| S563 | Injury of extensor or abductor muscles and tendons of thumb at forearm level | 86 | 86 | 100.00 |
| S564 | Injury of extensor muscle and tendon of other finger(s) at forearm level | 287 | 287 | 100.00 |
| S565 | Injury of other extensor muscle and tendon at forearm level | 145 | 145 | 100.00 |
| S567 | Injury of multiple muscles and tendons at forearm level | 68 | 67 | 98.53 |
| S568 | Injury of other and unspecified muscles and tendon at forearm level | 132 | 132 | 100.00 |
| S57 | Crushing injury of forearm | 30 | 30 | 100.00 |
| S570 | Crushing injury of elbow | 6 | 6 | 100.00 |
| S578 | Crushing injury of other parts of forearm | 16 | 16 | 100.00 |
| S579 | Crushing injury of forearm, part unspecified | 8 | 8 | 100.00 |
| S58 | Traumatic amputation of forearm | 26 | 26 | 100.00 |
| S580 | Traumatic amputation at elbow level | 3 | 3 | 100.00 |
| S581 | Traumatic amputation at level between elbow and wrist | 8 | 8 | 100.00 |
| S589 | Traumatic amputation of forearm, level unspecified | 15 | 15 | 100.00 |
| S59 | Other and unspecified injuries of forearm | 39 | 39 | 100.00 |
| S597 | Multiple injuries of forearm | 3 | 3 | 100.00 |
| S598 | Other specified injuries of forearm | 13 | 13 | 100.00 |
| S599 | Unspecified injury of forearm | 22 | 22 | 100.00 |
| S60 | Superficial injury of wrist and hand | 252 | 252 | 100.00 |
| S600 | Contusion of finger(s) without damage to nail | 33 | 33 | 100.00 |
| S601 | Contusion of finger(s) with damage to nail | 14 | 14 | 100.00 |
| S602 | Contusion of other parts of wrist and hand | 101 | 101 | 100.00 |
| S607 | Multiple superficial injuries of wrist and hand | 3 | 3 | 100.00 |
| S608 | Other superficial injuries of wrist and hand | 86 | 86 | 100.00 |
| S609 | Superficial injury of wrist and hand, unspecified | 15 | 15 | 100.00 |
| S61 | Open wound of wrist and hand | 5107 | 5106 | 99.98 |
| S610 | Open wound of finger(s) without damage to nail | 2432 | 2431 | 99.96 |
| S611 | Open wound of finger(s) with damage to nail | 700 | 700 | 100.00 |
| S617 | Multiple open wounds of wrist and hand | 229 | 229 | 100.00 |
| S618 | Open wound of other parts of wrist and hand | 826 | 826 | 100.00 |
| S619 | Open wound of wrist and hand, part unspecified | 910 | 910 | 100.00 |
| S62 | Fracture at wrist and hand level | 6345 | 6343 | 99.97 |
| S620 | Fracture of navicular [scaphoid] bone of hand | 86 | 85 | 98.84 |
| S621 | Fracture of other carpal bone(s) | 67 | 67 | 100.00 |
| S622 | Fracture of first metacarpal bone | 138 | 138 | 100.00 |
| S623 | Fracture of other metacarpal bone | 1124 | 1124 | 100.00 |
| S624 | Multiple fracture of metacarpal bones | 98 | 98 | 100.00 |
| S625 | Fracture of thumb | 567 | 567 | 100.00 |
| S626 | Fracture of other finger | 3925 | 3924 | 99.97 |
| S627 | Multiple fracture of fingers | 121 | 121 | 100.00 |
| S628 | Fracture of other and unspecified parts of wrist and hand | 205 | 205 | 100.00 |
| S63 | Dislocation, sprain and strain of joints and ligaments at wrist and hand level | 276 | 276 | 100.00 |
| S630 | Dislocation of wrist | 27 | 27 | 100.00 |
| S631 | Dislocation of finger | 90 | 90 | 100.00 |
| S632 | Multiple dislocations of fingers | 0 | 0 | 0.00 |
| S633 | Traumatic rupture of ligament of wrist and carpus | 9 | 9 | 100.00 |
| S634 | Traumatic rupture of ligament of finger at metacarpophalangeal and interphalangeal joint(s) | 60 | 60 | 100.00 |
| S635 | Sprain and strain of wrist | 62 | 62 | 100.00 |
| S636 | Sprain and strain of finger(s) | 23 | 23 | 100.00 |
| S637 | Sprain and strain of other and unspecified parts of hand | 5 | 5 | 100.00 |
| S64 | Injury of nerves at wrist and hand level | 286 | 286 | 100.00 |
| S640 | Injury of ulnar nerve at wrist and hand level | 18 | 18 | 100.00 |
| S641 | Injury of median nerve at wrist and hand level | 9 | 9 | 100.00 |
| S642 | Injury of radial nerve at wrist and hand level | 17 | 17 | 100.00 |
| S643 | Injury of digital nerve of thumb | 42 | 42 | 100.00 |
| S644 | Injury of digital nerve of other finger | 183 | 183 | 100.00 |
| S647 | Injury of multiple nerves at wrist and hand level | 0 | 0 | 0.00 |
| S648 | Injury of other nerves at wrist and hand level | 6 | 6 | 100.00 |
| S649 | Injury of unspecified nerve at wrist and hand level | 10 | 10 | 100.00 |
| S65 | Injury of blood vessels at wrist and hand level | 173 | 173 | 100.00 |
| S650 | Injury of ulnar artery at wrist and hand level | 8 | 8 | 100.00 |
| S651 | Injury of radial artery at wrist and hand level | 29 | 29 | 100.00 |
| S652 | Injury of superficial palmar arch | 4 | 4 | 100.00 |
| S653 | Injury of deep palmar arch | 3 | 3 | 100.00 |
| S654 | Injury of blood vessel(s) of thumb | 11 | 11 | 100.00 |
| S655 | Injury of blood vessel(s) of other finger | 68 | 68 | 100.00 |
| S657 | Injury of multiple blood vessels at wrist and hand level | 0 | 0 | 0.00 |
| S658 | Injury of other blood vessels at wrist and hand level | 36 | 36 | 100.00 |
| S659 | Injury of unspecified blood vessel at wrist and hand level | 14 | 14 | 100.00 |
| S66 | Injury of muscle and tendon at wrist and hand level | 5247 | 5247 | 100.00 |
| S660 | Injury of long flexor muscle and tendon of thumb at wrist and hand level | 119 | 119 | 100.00 |
| S661 | Injury of flexor muscle and tendon of other finger at wrist and hand level | 735 | 735 | 100.00 |
| S662 | Injury of extensor muscle and tendon of thumb at wrist and hand level | 762 | 762 | 100.00 |
| S663 | Injury of extensor muscle and tendon of other finger at wrist and hand level | 2684 | 2684 | 100.00 |
| S664 | Injury of intrinsic muscle and tendon of thumb at wrist and hand level | 71 | 71 | 100.00 |
| S665 | Injury of intrinsic muscle and tendon of other finger at wrist and hand level | 108 | 108 | 100.00 |
| S666 | Injury of multiple flexor muscles and tendons at wrist and hand level | 129 | 129 | 100.00 |
| S667 | Injury of multiple extensor muscles and tendons at wrist and hand level | 170 | 170 | 100.00 |
| S668 | Injury of other muscles and tendons at wrist and hand level | 295 | 295 | 100.00 |
| S669 | Injury of unspecified muscle and tendon at wrist and hand level | 171 | 171 | 100.00 |
| S67 | Crushing injury of wrist and hand | 952 | 952 | 100.00 |
| S670 | Crushing injury of thumb and other finger(s) | 830 | 830 | 100.00 |
| S678 | Crushing injury of other and unspecified parts of wrist and hand | 119 | 119 | 100.00 |
| S68 | Traumatic amputation of wrist and hand | 2530 | 2530 | 100.00 |
| S680 | Traumatic amputation of thumb (complete) (partial) | 327 | 327 | 100.00 |
| S681 | Traumatic amputation of other single finger (complete)(partial) | 1860 | 1860 | 100.00 |
| S682 | Traumatic amputation of two or more fingers alone (complete)(partial) | 196 | 196 | 100.00 |
| S683 | Combined traumatic amputation of (part of) finger(s) with other parts of wrist and hand | 62 | 62 | 100.00 |
| S684 | Traumatic amputation of hand at wrist level | 14 | 14 | 100.00 |
| S688 | Traumatic amputation of other parts of wrist and hand | 45 | 45 | 100.00 |
| S689 | Traumatic amputation of wrist and hand, level unspecified | 21 | 21 | 100.00 |
| S69 | Other and unspecified injuries of wrist and hand | 187 | 187 | 100.00 |
| S697 | Multiple injuries of wrist and hand | 3 | 3 | 100.00 |
| S698 | Other specified injuries of wrist and hand | 32 | 32 | 100.00 |
| S699 | Unspecified injury of wrist and hand | 152 | 152 | 100.00 |
| S70 | Superficial injury of hip and thigh | 927 | 927 | 100.00 |
| S700 | Contusion of hip | 555 | 555 | 100.00 |
| S701 | Contusion of thigh | 281 | 281 | 100.00 |
| S707 | Multiple superficial injuries of hip and thigh | 3 | 3 | 100.00 |
| S708 | Other superficial injuries of hip and thigh | 59 | 59 | 100.00 |
| S709 | Superficial injury of hip and thigh, unspecified | 28 | 28 | 100.00 |
| S71 | Open wound of hip and thigh | 371 | 371 | 100.00 |
| S710 | Open wound of hip | 60 | 60 | 100.00 |
| S711 | Open wound of thigh | 299 | 299 | 100.00 |
| S717 | Multiple open wounds of hip and thigh | 8 | 8 | 100.00 |
| S718 | Open wound of other and unspecified parts of pelvic girdle | 3 | 3 | 100.00 |
| S72 | Fracture of femur | 26714 | 26505 | 99.22 |
| S720 | Fracture of neck of femur | 9618 | 9563 | 99.43 |
| S721 | Pertrochanteric fracture | 11236 | 11124 | 99.00 |
| S722 | Subtrochanteric fracture | 770 | 765 | 99.35 |
| S723 | Fracture of shaft of femur | 2212 | 2203 | 99.59 |
| S724 | Fracture of lower end of femur | 1172 | 1167 | 99.57 |
| S727 | Multiple fractures of femur | 8 | 8 | 100.00 |
| S728 | Fractures of other parts of femur | 321 | 316 | 98.44 |
| S729 | Fracture of femur, part unspecified | 1339 | 1321 | 98.66 |
| S73 | Dislocation, sprain and strain of joint and ligaments of hip | 588 | 588 | 100.00 |
| S730 | Dislocation of hip | 420 | 420 | 100.00 |
| S731 | Sprain and strain of hip | 168 | 168 | 100.00 |
| S74 | Injury of nerves at hip and thigh level | 3 | 3 | 100.00 |
| S740 | Injury of sciatic nerve at hip and thigh level | 3 | 3 | 100.00 |
| S741 | Injury of femoral nerve at hip and thigh level | 0 | 0 | 0.00 |
| S742 | Injury of cutaneous sensory nerve at hip and thigh level | 0 | 0 | 0.00 |
| S747 | Injury of multiple nerves at hip and thigh level | 0 | 0 | 0.00 |
| S748 | Injury of other nerves at hip and thigh level | 0 | 0 | 0.00 |
| S749 | Injury of unspecified nerve at hip and thigh level | 0 | 0 | 0.00 |
| S75 | Injury of blood vessels at hip and thigh level | 43 | 42 | 97.67 |
| S750 | Injury of femoral artery | 17 | 16 | 94.12 |
| S751 | Injury of femoral vein at hip and thigh level | 5 | 5 | 100.00 |
| S752 | Injury of greater saphenous vein at hip and thigh level | 0 | 0 | 0.00 |
| S757 | Injury of multiple blood vessels at hip and thigh level | 0 | 0 | 0.00 |
| S758 | Injury of other blood vessels at hip and thigh level | 14 | 14 | 100.00 |
| S759 | Injury of unspecified blood vessel at hip and thigh level | 7 | 7 | 100.00 |
| S76 | Injury of muscle and tendon at hip and thigh level | 626 | 626 | 100.00 |
| S760 | Injury of muscle and tendon of hip | 42 | 42 | 100.00 |
| S761 | Injury of quadriceps muscle and tendon | 270 | 270 | 100.00 |
| S762 | Injury of adductor muscle and tendon of thigh | 36 | 36 | 100.00 |
| S763 | Injury of muscle and tendon of the posterior muscle group at thigh level | 72 | 72 | 100.00 |
| S764 | Injury of other and unspecified muscles and tendons at thigh level | 162 | 162 | 100.00 |
| S767 | Injury of multiple muscles and tendons at hip and thigh level | 43 | 43 | 100.00 |
| S77 | Crushing injury of hip and thigh | 6 | 6 | 100.00 |
| S770 | Crushing injury of hip | 2 | 2 | 100.00 |
| S771 | Crushing injury of thigh | 2 | 2 | 100.00 |
| S772 | Crushing injury of hip with thigh | 2 | 2 | 100.00 |
| S78 | Traumatic amputation of hip and thigh | 0 | 0 | 0.00 |
| S780 | Traumatic amputation at hip joint | 0 | 0 | 0.00 |
| S781 | Traumatic amputation at level between hip and knee | 0 | 0 | 0.00 |
| S789 | Traumatic amputation of hip and thigh, level unspecified | 0 | 0 | 0.00 |
| S79 | Other and unspecified injuries of hip and thigh | 27 | 27 | 100.00 |
| S797 | Multiple injuries of hip and thigh | 2 | 2 | 100.00 |
| S798 | Other specified injuries of hip and thigh | 10 | 10 | 100.00 |
| S799 | Unspecified injury of hip and thigh | 15 | 15 | 100.00 |
| S80 | Superficial injury of lower leg | 1426 | 1426 | 100.00 |
| S800 | Contusion of knee | 963 | 963 | 100.00 |
| S801 | Contusion of other and unspecified parts of lower leg | 381 | 381 | 100.00 |
| S807 | Multiple superficial injuries of lower leg | 2 | 2 | 100.00 |
| S808 | Other superficial injuries of lower leg | 57 | 57 | 100.00 |
| S809 | Superficial injury of lower leg, unspecified | 23 | 23 | 100.00 |
| S81 | Open wound of lower leg | 1662 | 1661 | 99.94 |
| S810 | Open wound of knee | 696 | 695 | 99.86 |
| S817 | Multiple open wounds of lower leg | 82 | 82 | 100.00 |
| S818 | Open wound of other parts of lower leg | 363 | 363 | 100.00 |
| S819 | Open wound of lower leg, part unspecified | 517 | 517 | 100.00 |
| S82 | Fracture of lower leg, including ankle | 20339 | 20325 | 99.93 |
| S820 | Fracture of patella | 2969 | 2969 | 100.00 |
| S821 | Fracture of upper end of tibia | 1727 | 1725 | 99.88 |
| S822 | Fracture of shaft of tibia | 3606 | 3602 | 99.89 |
| S823 | Fracture of lower end of tibia | 3022 | 3019 | 99.90 |
| S824 | Fracture of fibula alone | 612 | 611 | 99.84 |
| S825 | Fracture of medial malleolus | 837 | 836 | 99.88 |
| S826 | Fracture of lateral malleolus | 2719 | 2719 | 100.00 |
| S827 | Multiple fractures of lower leg | 44 | 43 | 97.73 |
| S828 | Fractures of other parts of lower leg | 4600 | 4599 | 99.98 |
| S829 | Fracture of lower leg, part unspecified | 139 | 138 | 99.28 |
| S83 | Dislocation, sprain and strain of joints and ligaments of knee | 1179 | 1178 | 99.92 |
| S830 | Dislocation of patella | 108 | 108 | 100.00 |
| S831 | Dislocation of knee | 38 | 38 | 100.00 |
| S832 | Tear of meniscus, current | 170 | 170 | 100.00 |
| S833 | Tear of articular cartilage of knee, current | 4 | 4 | 100.00 |
| S834 | Sprain and strain involving (fibular)(tibial) collateral ligament of knee | 181 | 181 | 100.00 |
| S835 | Sprain and strain involving (anterior) (posterior) cruciate ligament of knee | 228 | 228 | 100.00 |
| S836 | Sprain and strain of other and unspecified parts of knee | 421 | 420 | 99.76 |
| S837 | Injury to multiple structures of knee | 25 | 25 | 100.00 |
| S84 | Injury of nerves at lower leg level | 9 | 9 | 100.00 |
| S840 | Injury of tibial nerve at lower leg level | 1 | 1 | 100.00 |
| S841 | Injury of peroneal nerve at lower leg level | 7 | 7 | 100.00 |
| S842 | Injury of cutaneous sensory nerve at lower leg level | 0 | 0 | 0.00 |
| S847 | Injury of multiple nerves at lower leg level | 0 | 0 | 0.00 |
| S848 | Injury of other nerves at lower leg level | 1 | 1 | 100.00 |
| S849 | Injury of unspecified nerve at lower leg level | 0 | 0 | 0.00 |
| S85 | Injury of blood vessels at lower leg level | 37 | 37 | 100.00 |
| S850 | Injury of popliteal artery | 10 | 10 | 100.00 |
| S851 | Injury of (anterior)(posterior) tibial artery | 7 | 7 | 100.00 |
| S852 | Injury of peroneal artery | 0 | 0 | 0.00 |
| S853 | Injury of greater saphenous vein at lower leg level | 0 | 0 | 0.00 |
| S854 | Injury of lesser saphenous vein at lower leg level | 1 | 1 | 100.00 |
| S855 | Injury of popliteal vein | 0 | 0 | 0.00 |
| S857 | Injury of multiple blood vessels at lower leg level | 1 | 1 | 100.00 |
| S858 | Injury of other blood vessels at lower leg level | 7 | 7 | 100.00 |
| S859 | Injury of unspecified blood vessel at lower leg level | 11 | 11 | 100.00 |
| S86 | Injury of muscle and tendon at lower leg level | 2888 | 2886 | 99.93 |
| S860 | Injury of Achilles tendon | 2116 | 2115 | 99.95 |
| S861 | Injury of other muscle(s) and tendon(s) of posterior muscle group at lower leg level | 158 | 158 | 100.00 |
| S862 | Injury of muscle(s) and tendon(s) of anterior muscle group at lower leg level | 127 | 127 | 100.00 |
| S863 | Injury of muscle(s) and tendon(s) of peroneal muscle group at lower leg level | 58 | 58 | 100.00 |
| S867 | Injury of multiple muscles and tendons at lower leg level | 63 | 62 | 98.41 |
| S868 | Injury of other muscles and tendons at lower leg level | 221 | 221 | 100.00 |
| S869 | Injury of unspecified muscles and tendons at lower leg level | 133 | 133 | 100.00 |
| S87 | Crushing injury of lower leg | 36 | 36 | 100.00 |
| S870 | Crushing injury of knee | 10 | 10 | 100.00 |
| S878 | Crushing injury of other and unspecified parts of lower leg | 26 | 26 | 100.00 |
| S88 | Traumatic amputation of lower leg | 7 | 7 | 100.00 |
| S880 | Traumatic amputation at knee level | 1 | 1 | 100.00 |
| S881 | Traumatic amputation at level between knee and ankle | 5 | 5 | 100.00 |
| S889 | Traumatic amputation of lower leg, level unspecified | 1 | 1 | 100.00 |
| S89 | Other and unspecified injuries of lower leg | 46 | 46 | 100.00 |
| S897 | Multiple injuries of lower leg | 0 | 0 | 0.00 |
| S898 | Other specified injuries of lower leg | 15 | 15 | 100.00 |
| S899 | Unspecified injury of lower leg | 31 | 31 | 100.00 |
| S90 | Superficial injury of ankle and foot | 595 | 595 | 100.00 |
| S900 | Contusion of ankle | 146 | 146 | 100.00 |
| S901 | Contusion of toe(s) without damage to nail | 32 | 32 | 100.00 |
| S902 | Contusion of toe(s) with damage to nail | 12 | 12 | 100.00 |
| S903 | Contusion of other and unspecified parts of foot | 354 | 354 | 100.00 |
| S907 | Multiple superficial injuries of ankle and foot | 1 | 1 | 100.00 |
| S908 | Other superficial injuries of ankle and foot | 44 | 44 | 100.00 |
| S909 | Superficial injury of ankle and foot, unspecified | 6 | 6 | 100.00 |
| S91 | Open wound of ankle and foot | 1022 | 1022 | 100.00 |
| S910 | Open wound of ankle | 174 | 174 | 100.00 |
| S911 | Open wound of toe(s) without damage to nail | 141 | 141 | 100.00 |
| S912 | Open wound of toe(s) with damage to nail | 85 | 85 | 100.00 |
| S913 | Open wound of other parts of foot | 572 | 572 | 100.00 |
| S917 | Multiple open wounds of ankle and foot | 48 | 48 | 100.00 |
| S92 | Fracture of foot, except ankle | 6583 | 6580 | 99.95 |
| S920 | Fracture of calcaneus | 2658 | 2657 | 99.96 |
| S921 | Fracture of talus | 173 | 172 | 99.42 |
| S922 | Fracture of other tarsal bone(s) | 172 | 172 | 100.00 |
| S923 | Fracture of metatarsal bone | 1955 | 1954 | 99.95 |
| S924 | Fracture of great toe | 627 | 627 | 100.00 |
| S925 | Fracture of other toe | 787 | 787 | 100.00 |
| S927 | Multiple fractures of foot | 83 | 83 | 100.00 |
| S929 | Fracture of foot, unspecified | 110 | 110 | 100.00 |
| S93 | Dislocation, sprain and strain of joints and ligaments at ankle and foot level | 968 | 968 | 100.00 |
| S930 | Dislocation of ankle joint | 47 | 47 | 100.00 |
| S931 | Dislocation of toe(s) | 46 | 46 | 100.00 |
| S932 | Rupture of ligaments at ankle and foot level | 148 | 148 | 100.00 |
| S933 | Dislocation of other and unspecified parts of foot | 28 | 28 | 100.00 |
| S934 | Sprain and strain of ankle | 600 | 600 | 100.00 |
| S935 | Sprain and strain of toe(s) | 10 | 10 | 100.00 |
| S936 | Sprain and strain of other and unspecified parts of foot | 86 | 86 | 100.00 |
| S94 | Injury of nerves at ankle and foot level | 12 | 12 | 100.00 |
| S940 | Injury of lateral plantar nerve | 0 | 0 | 0.00 |
| S941 | Injury of medial plantar nerve | 0 | 0 | 0.00 |
| S942 | Injury of deep peroneal nerve at ankle and foot level | 3 | 3 | 100.00 |
| S943 | Injury of cutaneous sensory nerve at ankle and foot level | 2 | 2 | 100.00 |
| S947 | Injury of multiple nerves at ankle and foot level | 0 | 0 | 0.00 |
| S948 | Injury of other nerves at ankle and foot level | 4 | 4 | 100.00 |
| S949 | Injury of unspecified nerve at ankle and foot level | 3 | 3 | 100.00 |
| S95 | Injury of blood vessels at ankle and foot level | 21 | 21 | 100.00 |
| S950 | Injury of dorsal artery of foot | 6 | 6 | 100.00 |
| S951 | Injury of plantar artery of foot | 0 | 0 | 0.00 |
| S952 | Injury of dorsal vein of foot | 1 | 1 | 100.00 |
| S957 | Injury of multiple blood vessels at ankle and foot level | 0 | 0 | 0.00 |
| S958 | Injury of other blood vessels at ankle and foot level | 6 | 6 | 100.00 |
| S959 | Injury of unspecified blood vessel at ankle and foot level | 8 | 8 | 100.00 |
| S96 | Injury of muscle and tendon at ankle and foot level | 725 | 725 | 100.00 |
| S960 | Injury of muscle and tendon of long flexor muscle of toe at ankle and foot level | 69 | 69 | 100.00 |
| S961 | Injury of muscle and tendon of long extensor muscle of toe at ankle and foot level | 398 | 398 | 100.00 |
| S962 | Injury of intrinsic muscle and tendon at ankle and foot level | 31 | 31 | 100.00 |
| S967 | Injury of multiple muscles and tendons at ankle and foot level | 32 | 32 | 100.00 |
| S968 | Injury of other muscles and tendons at ankle and foot level | 116 | 116 | 100.00 |
| S969 | Injury of unspecified muscle tendon at ankle and foot level | 79 | 79 | 100.00 |
| S97 | Crushing injury of ankle and foot | 78 | 78 | 100.00 |
| S970 | Crushing injury of ankle | 7 | 7 | 100.00 |
| S971 | Crushing injury of toe(s) | 20 | 20 | 100.00 |
| S978 | Crushing injury of other parts of ankle and foot | 50 | 50 | 100.00 |
| S98 | Traumatic amputation of ankle and foot | 57 | 56 | 98.25 |
| S980 | Traumatic amputation of foot at ankle level | 3 | 3 | 100.00 |
| S981 | Traumatic amputation of one toe | 39 | 39 | 100.00 |
| S982 | Traumatic amputation of two or more toes | 6 | 6 | 100.00 |
| S983 | Traumatic amputation of other parts of foot | 6 | 5 | 83.33 |
| S984 | Traumatic amputation of foot, level unspecified | 3 | 3 | 100.00 |
| S99 | Other and unspecified injuries of ankle and foot | 42 | 42 | 100.00 |
| S997 | Multiple injuries of ankle and foot | 2 | 2 | 100.00 |
| S998 | Other specified injuries of ankle and foot | 8 | 8 | 100.00 |
| S999 | Unspecified injury of ankle and foot | 32 | 32 | 100.00 |
| T00 | Superficial injuries involving multiple body regions | 2456 | 2452 | 99.84 |
| T000 | Superficial injuries involving head with neck | 3 | 3 | 100.00 |
| T001 | Superficial injuries involving thorax with abdomen, lower back and pelvis | 0 | 0 | 0.00 |
| T002 | Superficial injuries involving multiple regions of upper limb(s) | 0 | 0 | 0.00 |
| T003 | Superficial injuries involving multiple regions of lower limb(s) | 1 | 1 | 100.00 |
| T006 | Superficial injuries involving multiple regions of upper limb(s) with lower limb(s) | 0 | 0 | 0.00 |
| T008 | Superficial injuries involving other combinations of body regions | 47 | 47 | 100.00 |
| T009 | Multiple superficial injuries, unspecified | 2405 | 2401 | 99.83 |
| T01 | Open wounds involving multiple body regions | 61 | 61 | 100.00 |
| T010 | Open wounds involving head with neck | 4 | 4 | 100.00 |
| T011 | Open wounds involving thorax with abdomen, lower back and pelvis | 2 | 2 | 100.00 |
| T012 | Open wounds involving multiple regions of upper limb(s) | 7 | 7 | 100.00 |
| T013 | Open wounds involving multiple regions of lower limb(s) | 1 | 1 | 100.00 |
| T016 | Open wounds involving multiple regions of upper limb(s) with lower limb(s) | 1 | 1 | 100.00 |
| T018 | Open wounds involving other combinations of body regions | 4 | 4 | 100.00 |
| T019 | Multiple open wounds, unspecified | 42 | 42 | 100.00 |
| T02 | Fractures involving multiple body regions | 32 | 30 | 93.75 |
| T020 | Fractures involving head with neck | 0 | 0 | 0.00 |
| T021 | Fractures involving thorax with lower back and pelvis | 7 | 7 | 100.00 |
| T022 | Fractures involving multiple regions of one upper limb | 0 | 0 | 0.00 |
| T023 | Fractures involving multiple regions of one lower limb | 2 | 2 | 100.00 |
| T024 | Fractures involving multiple regions of both upper limbs | 0 | 0 | 0.00 |
| T025 | Fractures involving multiple regions of both lower limbs | 0 | 0 | 0.00 |
| T026 | Fractures involving multiple regions of upper limb(s) with lower limb(s) | 0 | 0 | 0.00 |
| T027 | Fractures involving thorax with lower back and pelvis with limb(s) | 1 | 1 | 100.00 |
| T028 | Fractures involving other combinations of body regions | 18 | 18 | 100.00 |
| T029 | Multiple fractures, unspecified | 4 | 2 | 50.00 |
| T03 | Dislocations, sprains and strains involving multiple body regions | 28 | 28 | 100.00 |
| T030 | Dislocations, sprains and strains involving head with neck | 3 | 3 | 100.00 |
| T031 | Dislocations, sprains and strains involving thorax with lower back and pelvis | 3 | 3 | 100.00 |
| T032 | Dislocations, sprains and strains involving multiple regions of upper limb(s) | 0 | 0 | 0.00 |
| T033 | Dislocations, sprains and strains involving multiple regions of lower limb(s) | 0 | 0 | 0.00 |
| T034 | Dislocations, sprains and strains involving multiple regions of upper limb(s) with lower limb(s) | 0 | 0 | 0.00 |
| T038 | Dislocations, sprains and strains involving other combinations of body regions | 5 | 5 | 100.00 |
| T039 | Multiple dislocations, sprains and strains, unspecified | 17 | 17 | 100.00 |
| T04 | Crushing injuries involving multiple body regions | 10 | 10 | 100.00 |
| T040 | Crushing injuries involving head with neck | 0 | 0 | 0.00 |
| T041 | Crushing injuries involving thorax with abdomen, lower back and pelvis | 0 | 0 | 0.00 |
| T042 | Crushing injuries involving multiple regions of upper limb(s) | 2 | 2 | 100.00 |
| T043 | Crushing injuries involving multiple regions of lower limb(s) | 6 | 6 | 100.00 |
| T044 | Crushing injuries involving multiple regions of upper limb(s) with lower limb(s) | 1 | 1 | 100.00 |
| T047 | Crushing injuries of thorax with abdomen, lower back and pelvis with limb(s) | 0 | 0 | 0.00 |
| T048 | Crushing injuries involving other combinations of body regions | 0 | 0 | 0.00 |
| T049 | Multiple crushing injuries, unspecified | 1 | 1 | 100.00 |
| T05 | Traumatic amputations involving multiple body regions | 9 | 9 | 100.00 |
| T050 | Traumatic amputation of both hands | 1 | 1 | 100.00 |
| T051 | Traumatic amputation of one hand and other arm [any level, except hand] | 2 | 2 | 100.00 |
| T052 | Traumatic amputation of both arm [any level] | 0 | 0 | 0.00 |
| T053 | Traumatic amputation of both feet | 0 | 0 | 0.00 |
| T054 | Traumatic amputation of one foot and other leg [any level, except foot] | 2 | 2 | 100.00 |
| T055 | Traumatic amputation of both legs [any level] | 0 | 0 | 0.00 |
| T056 | Traumatic amputation of upper and lower limbs, any combination [any level] | 0 | 0 | 0.00 |
| T058 | Traumatic amputation involving other combinations of body regions | 0 | 0 | 0.00 |
| T059 | Multiple traumatic amputations, unspecified | 4 | 4 | 100.00 |
| T06 | Other injuries involving multiple body regions, NEC | 12 | 11 | 91.67 |
| T060 | Injuries of brain and cranial nerves with injuries of nerves and spinal cord at neck level | 2 | 2 | 100.00 |
| T061 | Injuries of nerves and spinal cord involving other multiple body regions | 2 | 2 | 100.00 |
| T062 | Injuries of nerves involving multiple body regions | 0 | 0 | 0.00 |
| T063 | Injuries of blood vessels involving multiple body regions | 0 | 0 | 0.00 |
| T064 | Injuries of muscles and tendons involving multiple body regions | 1 | 1 | 100.00 |
| T065 | Injuries of intrathoracic organs with intra-abdominal and pelvic organs | 0 | 0 | 0.00 |
| T068 | Other specified injuries involving multiple body regions | 7 | 6 | 85.71 |
| T07 | Unspecified multiple injuries | 36 | 34 | 94.44 |
| T08 | Fracture of spine, level unspecified | 213 | 213 | 100.00 |
| T080 | Closed | 210 | 210 | 100.00 |
| T081 | Open | 0 | 0 | 0.00 |
| T09 | Other injuries of spine and trunk, level unspecified | 243 | 238 | 97.94 |
| T090 | Superficial injury of trunk, level unspecified | 9 | 9 | 100.00 |
| T091 | Open wound of trunk, level unspecified | 19 | 19 | 100.00 |
| T092 | Dislocation, sprain and strain of unspecified joint and ligament of trunk | 70 | 70 | 100.00 |
| T093 | Injury of spinal cord, level unspecified | 124 | 119 | 95.97 |
| T094 | Injury of unspecified nerve, spinal nerve root and plexus of trunk | 12 | 12 | 100.00 |
| T095 | Injury of unspecified muscle and tendon of trunk | 2 | 2 | 100.00 |
| T096 | Traumatic amputation of trunk, level unspecified | 0 | 0 | 0.00 |
| T098 | Other specified injuries of trunk, level unspecified | 1 | 1 | 100.00 |
| T099 | Unspecified injury of trunk, level unspecified | 5 | 5 | 100.00 |
| T10 | Fracture of upper limb, level unspecified | 5 | 5 | 100.00 |
| T100 | Closed | 2 | 2 | 100.00 |
| T101 | Open | 2 | 2 | 100.00 |
| T11 | Other injuries of upper limb, level unspecified | 34 | 34 | 100.00 |
| T110 | Superficial injury of upper limb, level unspecified | 4 | 4 | 100.00 |
| T111 | Open wound of upper limb, level unspecified | 17 | 17 | 100.00 |
| T112 | Dislocation, sprain and strain of unspecified joint and ligament of trunk joint and ligament of upper limb, level unspecified | 1 | 1 | 100.00 |
| T113 | Injury of unspecified nerve of upper limb, level unspecified | 0 | 0 | 0.00 |
| T114 | Injury of unspecified blood vessel of upper limb, level unspecified | 0 | 0 | 0.00 |
| T115 | Injury of unspecified muscle and tendon of upper limb, level unspecified | 7 | 7 | 100.00 |
| T116 | Traumatic amputation of upper limb, level unspecified | 3 | 3 | 100.00 |
| T118 | Other specified injuries of upper limb, level unspecified | 1 | 1 | 100.00 |
| T119 | Unspecified injury of upper limb, level unspecified | 1 | 1 | 100.00 |
| T12 | Fracture of lower limb, level unspecified | 9 | 9 | 100.00 |
| T120 | Closed | 6 | 6 | 100.00 |
| T121 | Open | 2 | 2 | 100.00 |
| T13 | Other injuries of lower limb, level unspecified | 77 | 77 | 100.00 |
| T130 | Superficial injury of lower limb, level unspecified | 4 | 4 | 100.00 |
| T131 | Open wound of lower limb, level unspecified | 39 | 39 | 100.00 |
| T132 | Dislocation, sprain and strain of unspecified joint and ligament of lower limb, level unspecified | 14 | 14 | 100.00 |
| T133 | Injury of unspecified nerve of lower limb, level unspecified | 0 | 0 | 0.00 |
| T134 | Injury of unspecified blood vessel of lower limb, level unspecified | 1 | 1 | 100.00 |
| T135 | Injury of unspecified muscle and tendon of lower limb, level unspecified | 11 | 11 | 100.00 |
| T136 | Traumatic amputation of lower limb, level unspecified | 1 | 1 | 100.00 |
| T138 | Other specified injuries of lower limb, level unspecified | 0 | 0 | 0.00 |
| T139 | Unspecified injury of lower limb, level unspecified | 7 | 7 | 100.00 |
| T14 | Injury of unspecified body region | 2151 | 2151 | 100.00 |
| T140 | Superficial injury of unspecified body region | 798 | 798 | 100.00 |
| T141 | Open wound of unspecified body region | 942 | 942 | 100.00 |
| T142 | Fracture of unspecified body region | 28 | 28 | 100.00 |
| T143 | Dislocation, sprain and strain of unspecified body region | 30 | 30 | 100.00 |
| T144 | Injury of nerve(s) of unspecified body region | 2 | 2 | 100.00 |
| T145 | Injury of blood vessel(s) of unspecified body region | 3 | 3 | 100.00 |
| T146 | Injury of muscles and tendons of unspecified body region | 211 | 211 | 100.00 |
| T147 | Crushing injury and traumatic amputation of unspecified body region | 127 | 127 | 100.00 |
| T148 | Other injuries of unspecified body region | 0 | 0 | 0.00 |
| T149 | Injury, unspecified | 9 | 9 | 100.00 |
| T15 | Foreign body on external eye | 79 | 79 | 100.00 |
| T150 | Foreign body in cornea | 49 | 49 | 100.00 |
| T151 | Foreign body in conjunctival sac | 5 | 5 | 100.00 |
| T158 | Foreign body in other and multiple parts of external eye | 1 | 1 | 100.00 |
| T159 | Foreign body on external eye, part unspecified | 24 | 24 | 100.00 |
| T16 | Foreign body in ear | 63 | 63 | 100.00 |
| T17 | Foreign body in respiratory tract | 438 | 436 | 99.54 |
| T170 | Foreign body in nasal sinus | 8 | 8 | 100.00 |
| T171 | Foreign body in nostril | 35 | 35 | 100.00 |
| T172 | Foreign body in pharynx | 191 | 191 | 100.00 |
| T173 | Foreign body in larynx | 29 | 29 | 100.00 |
| T174 | Foreign body in trachea | 16 | 16 | 100.00 |
| T175 | Foreign body in bronchus | 96 | 96 | 100.00 |
| T178 | Foreign body in other and multiple parts of respiratory tract | 7 | 7 | 100.00 |
| T179 | Foreign body in respiratory tract, part unspecified | 55 | 53 | 96.36 |
| T18 | Foreign body in alimentary tract | 1987 | 1987 | 100.00 |
| T180 | Foreign body in mouth | 5 | 5 | 100.00 |
| T181 | Foreign body in esophagus | 1048 | 1048 | 100.00 |
| T182 | Foreign body in stomach | 233 | 233 | 100.00 |
| T183 | Foreign body in small intestine | 113 | 113 | 100.00 |
| T184 | Foreign body in colon | 48 | 48 | 100.00 |
| T185 | Foreign body in anus and rectum | 124 | 124 | 100.00 |
| T188 | Foreign body in other and multiple parts of alimentary tract | 7 | 7 | 100.00 |
| T189 | Foreign body in alimentary tract, part unspecified | 408 | 408 | 100.00 |
| T19 | Foreign body in genitourinary tract | 40 | 40 | 100.00 |
| T190 | Foreign body in urethra | 14 | 14 | 100.00 |
| T191 | Foreign body in bladder | 9 | 9 | 100.00 |
| T192 | Foreign body in vulva and vagina | 9 | 9 | 100.00 |
| T193 | Foreign body in uterus[any part] | 0 | 0 | 0.00 |
| T198 | Foreign body in other and multiple parts of genitourinary tract | 5 | 5 | 100.00 |
| T199 | Foreign body in genitourinary tract, part unspecified | 3 | 3 | 100.00 |
| T20 | Burn and corrosion of head and neck | 682 | 674 | 98.83 |
| T200 | Burn of unspecified degree of head and neck | 18 | 18 | 100.00 |
| T201 | Burn of first degree of head and neck | 15 | 15 | 100.00 |
| T202 | Burn of second degree of head and neck | 641 | 633 | 98.75 |
| T203 | Burn of third degree of head and neck | 6 | 6 | 100.00 |
| T204 | Corrosion of unspecified degree of head and neck | 0 | 0 | 0.00 |
| T205 | Corrosion of first degree of head and neck | 0 | 0 | 0.00 |
| T206 | Corrosion of second degree of head and neck | 0 | 0 | 0.00 |
| T207 | Corrosion of third degree of head and neck | 2 | 2 | 100.00 |
| T21 | Burn and corrosion of trunk | 311 | 296 | 95.18 |
| T210 | Burn of unspecified degree of trunk | 5 | 4 | 80.00 |
| T211 | Burn of first degree of trunk | 3 | 3 | 100.00 |
| T212 | Burn of second degree of trunk | 293 | 281 | 95.90 |
| T213 | Burn of third degree of trunk | 10 | 8 | 80.00 |
| T214 | Corrosion of unspecified degree of trunk | 0 | 0 | 0.00 |
| T215 | Corrosion of first degree of trunk | 0 | 0 | 0.00 |
| T216 | Corrosion of second degree of trunk | 0 | 0 | 0.00 |
| T217 | Corrosion of third degree of trunk | 0 | 0 | 0.00 |
| T22 | Burn and corrosion of shoulder and upper limb, except wrist and hand | 164 | 163 | 99.39 |
| T220 | Burn of unspecified degree of shoulder and upper limb, except wrist and hand | 5 | 5 | 100.00 |
| T221 | Burn of first degree of shoulder and upper limb, except wrist and hand | 1 | 1 | 100.00 |
| T222 | Burn of second degree of shoulder and upper limb, except wrist and hand | 145 | 144 | 99.31 |
| T223 | Burn of third degree of shoulder and upper limb, except wrist and hand | 12 | 12 | 100.00 |
| T224 | Corrosion of unspecified degree of shoulder and upper limb, except wrist and hand | 0 | 0 | 0.00 |
| T225 | Corrosion of first degree of shoulder and upper limb, except wrist and hand | 0 | 0 | 0.00 |
| T226 | Corrosion of second degree of shoulder and upper limb, except wrist and hand | 1 | 1 | 100.00 |
| T227 | Corrosion of third degree of shoulder and upper limb, except wrist and hand | 0 | 0 | 0.00 |
| T23 | Burn and corrosion of wrist and hand | 357 | 355 | 99.44 |
| T230 | Burn of unspecified degree of wrist and hand | 19 | 19 | 100.00 |
| T231 | Burn of first degree of wrist and hand | 3 | 3 | 100.00 |
| T232 | Burn of second degree of wrist and hand | 302 | 300 | 99.34 |
| T233 | Burn of third degree of wrist and hand | 31 | 31 | 100.00 |
| T234 | Corrosion of unspecified degree of wrist and hand | 0 | 0 | 0.00 |
| T235 | Corrosion of first degree of wrist and hand | 0 | 0 | 0.00 |
| T236 | Corrosion of second degree of wrist and hand | 1 | 1 | 100.00 |
| T237 | Corrosion of third degree of wrist and hand | 0 | 0 | 0.00 |
| T24 | Burn and corrosion of hip and lower limb, except ankle and foot | 592 | 589 | 99.49 |
| T240 | Burn of unspecified degree of hip and lower limb, except ankle and foot | 8 | 8 | 100.00 |
| T241 | Burn of first degree of hip and lower limb, except ankle and foot | 4 | 4 | 100.00 |
| T242 | Burn of second degree of hip and lower limb, except ankle and foot | 552 | 549 | 99.46 |
| T243 | Burn of third degree of hip and lower limb, except ankle and foot | 24 | 24 | 100.00 |
| T244 | Corrosion of unspecified degree of hip and lower limb, except ankle and foot | 0 | 0 | 0.00 |
| T245 | Corrosion of first degree of hip and lower limb, except ankle and foot | 0 | 0 | 0.00 |
| T246 | Corrosion of second degree of hip and lower limb, except ankle and foot | 2 | 2 | 100.00 |
| T247 | Corrosion of third degree of hip and lower limb, except ankle and foot | 2 | 2 | 100.00 |
| T25 | Burn and corrosion of ankle and foot | 313 | 310 | 99.04 |
| T250 | Burn of unspecified degree of ankle and foot | 9 | 9 | 100.00 |
| T251 | Burn of first degree of ankle and foot | 4 | 4 | 100.00 |
| T252 | Burn of second degree of ankle and foot | 279 | 277 | 99.28 |
| T253 | Burn of third degree of ankle and foot | 18 | 17 | 94.44 |
| T254 | Corrosion unspecified degree of ankle and foot | 0 | 0 | 0.00 |
| T255 | Corrosion of first degree of ankle and foot | 0 | 0 | 0.00 |
| T256 | Corrosion of second degree of ankle and foot | 1 | 1 | 100.00 |
| T257 | Corrosion of third degree of ankle and foot | 2 | 2 | 100.00 |
| T26 | Burn and corrosion confined to eye and adnexa | 53 | 53 | 100.00 |
| T260 | Burn of eyelid and periocular area | 2 | 2 | 100.00 |
| T261 | Burn of cornea and conjunctival sac | 31 | 31 | 100.00 |
| T262 | Burn with resulting rupture and destruction of eyeball | 0 | 0 | 0.00 |
| T263 | Burn of other parts of eye and adnexa | 1 | 1 | 100.00 |
| T264 | Burn of eye and adnexa, part unspecified | 4 | 4 | 100.00 |
| T265 | Corrosion of eyelid and periocular area | 1 | 1 | 100.00 |
| T266 | Corrosion of cornea and conjunctival sac | 10 | 10 | 100.00 |
| T267 | Corrosion with resulting rupture and destruction of eyeball | 3 | 3 | 100.00 |
| T268 | Corrosion of other parts of eye and adnexa | 0 | 0 | 0.00 |
| T269 | Corrosion of eye and adnexa, part unspecified | 1 | 1 | 100.00 |
| T27 | Burn and corrosion of respiratory tract | 67 | 67 | 100.00 |
| T270 | Burn of larynx and trachea | 7 | 7 | 100.00 |
| T271 | Burn involving larynx and trachea with lung | 9 | 9 | 100.00 |
| T272 | Burn of other parts of respiratory tract | 7 | 7 | 100.00 |
| T273 | Burn of respiratory tract, part unspecified | 39 | 39 | 100.00 |
| T274 | Corrosion of larynx and trachea | 0 | 0 | 0.00 |
| T275 | Corrosion involving larynx and trachea with lung | 0 | 0 | 0.00 |
| T276 | Corrosion of other parts of respiratory tract | 1 | 1 | 100.00 |
| T277 | Corrosion of respiratory tract, part unspecified | 2 | 2 | 100.00 |
| T28 | Burn and corrosion of other internal organs | 28 | 28 | 100.00 |
| T280 | Burn of mouth and pharynx | 3 | 3 | 100.00 |
| T281 | Burn of esophagus | 2 | 2 | 100.00 |
| T282 | Burn of other parts of alimentary tract | 0 | 0 | 0.00 |
| T283 | Burn of internal genitourinary organs | 0 | 0 | 0.00 |
| T284 | Burn of other and unspecified internal organs | 0 | 0 | 0.00 |
| T285 | Corrosion of mouth and pharynx | 3 | 3 | 100.00 |
| T286 | Corrosion of esophagus | 9 | 9 | 100.00 |
| T287 | Corrosion of other parts of alimentary tract | 10 | 10 | 100.00 |
| T288 | Corrosion of internal genitourinary organs | 0 | 0 | 0.00 |
| T289 | Corrosion of other and unspecified internal organs | 1 | 1 | 100.00 |
| T29 | Burns and corrosions of multiple body regions | 65 | 64 | 98.46 |
| T290 | Burns of multiple regions, unspecified degree | 17 | 16 | 94.12 |
| T291 | Burns of multiple regions, no more than first-degree burns mentioned | 0 | 0 | 0.00 |
| T292 | Burns of multiple regions, no more than second-degree burns mentioned | 44 | 44 | 100.00 |
| T293 | Burns of multiple regions, at least one burn of third degree mentioned | 4 | 4 | 100.00 |
| T294 | Corrosions of multiple regions, unspecified degree | 0 | 0 | 0.00 |
| T295 | Corrosions of multiple regions, no more than first-degree corrosions mentioned | 0 | 0 | 0.00 |
| T296 | Corrosions of multiple regions, no more than second-degree corrosions mentioned | 0 | 0 | 0.00 |
| T297 | Corrosions of multiple regions, at least one corrosion of third degree mentioned | 0 | 0 | 0.00 |
| T30 | Burn and corrosion, body region unspecified | 270 | 268 | 99.26 |
| T300 | Burn of unspecified body region, unspecified degree | 108 | 108 | 100.00 |
| T301 | Burn of first degree, body region unspecified | 1 | 1 | 100.00 |
| T302 | Burn of second degree, body region unspecified | 148 | 147 | 99.32 |
| T303 | Burn of third degree, body region unspecified | 1 | 0 | 0.00 |
| T304 | Corrosion of unspecified body region, unspecified degree | 12 | 12 | 100.00 |
| T305 | Corrosion of first degree, body region unspecified | 0 | 0 | 0.00 |
| T306 | Corrosion of second degree, body region unspecified | 0 | 0 | 0.00 |
| T307 | Corrosion of third degree, body region unspecified | 0 | 0 | 0.00 |
| T31 | Burns classified according to extent of body surface involved | 49 | 45 | 91.84 |
| T310 | Burns involving less than 10％ of body surface | 15 | 15 | 100.00 |
| T311 | Burns involving 10-19％ of body surface | 18 | 17 | 94.44 |
| T312 | Burns involving 20-29％ of body surface | 7 | 7 | 100.00 |
| T313 | Burns involving 30-39％ of body surface | 4 | 4 | 100.00 |
| T314 | Burns involving 40-49％ of body surface | 1 | 1 | 100.00 |
| T315 | Burns involving 50-59％ of body surface | 0 | 0 | 0.00 |
| T316 | Burns involving 60-69％ of body surface | 0 | 0 | 0.00 |
| T317 | Burns involving 70-79％ of body surface | 1 | 1 | 100.00 |
| T318 | Burns involving 80-89％ of body surface | 2 | 0 | 0.00 |
| T319 | Burns involving 90％ or more of body surface | 1 | 0 | 0.00 |
| T32 | Corrosions classified according to extent of body surface involved | 0 | 0 | 0.00 |
| T320 | Corrosions involving less than 10％ of body Corrosions involving 10-19％ of body surface | 0 | 0 | 0.00 |
| T321 | Corrosions involving 10-19％ of body surface | 0 | 0 | 0.00 |
| T322 | Corrosions involving 20-29％ of body surface | 0 | 0 | 0.00 |
| T323 | Corrosions involving 30-39％ of body surface | 0 | 0 | 0.00 |
| T324 | Corrosions involving 40-49％ of body surface | 0 | 0 | 0.00 |
| T325 | Corrosions involving 50-59％ of body surface | 0 | 0 | 0.00 |
| T326 | Corrosions involving 60-69％ of body surface | 0 | 0 | 0.00 |
| T327 | Corrosions involving 70-79％ of body surface | 0 | 0 | 0.00 |
| T328 | Corrosions involving 80-89％ of body surface | 0 | 0 | 0.00 |
| T329 | Corrosions involving 90％ or more of body surface | 0 | 0 | 0.00 |
| T33 | Superficial frostbite | 0 | 0 | excluded |
| T34 | Frostbite with tissue necrosis | 0 | 0 | excluded |
| T35 | Frostbite involving multiple body regions and unspecified frostbite | 0 | 0 | excluded |
| T36 | Poisoning by systemic antibiotics | 0 | 0 | excluded |
| T37 | Poisoning by other systemic anti-infectives and antiparasitic | 0 | 0 | excluded |
| T38 | Poisoning by hormones and their synthetic substitutes and antagonists, NEC | 0 | 0 | excluded |
| T39 | Poisoning by nonopioid analgesics, antipyretics and antirheumatics | 0 | 0 | excluded |
| T40 | Poisoning by narcotics and psychodysleptics [hallucinogens] | 0 | 0 | excluded |
| T41 | Poisoning by anesthetics and therapeutic gases | 0 | 0 | excluded |
| T42 | Poisoning by antiepileptic, sedative-hypnotic and antiparkinsonism drugs | 0 | 0 | excluded |
| T43 | Poisoning by psychotropic drugs, NEC | 0 | 0 | excluded |
| T44 | Poisoning by drugs primarily affecting the autonomic nervous system | 0 | 0 | excluded |
| T45 | Poisoning by primarily systemic and hematological agents, NEC | 0 | 0 | excluded |
| T46 | Poisoning by agents primarily affecting the cardiovascular system | 0 | 0 | excluded |
| T47 | Poisoning by agents primarily affecting the gastrointestinal system | 0 | 0 | excluded |
| T48 | Poisoning by agents primarily acting on smooth and skeletal muscles and the respiratory system | 0 | 0 | excluded |
| T49 | Poisoning by topical agents primarily affecting skin and mucous membrane and by ophthalmological, otorhinolaryngological and dental drugs | 0 | 0 | excluded |
| T50 | Poisoning by diuretics and other and unspecified drugs, medicaments and biological substances | 0 | 0 | excluded |
| T51 | Toxic effect of alcohol | 0 | 0 | excluded |
| T52 | Toxic effect of organic solvents | 0 | 0 | excluded |
| T53 | Toxic effect of halogen derivatives of aliphatic and aromatic hydrocarbons | 0 | 0 | excluded |
| T54 | Toxic effect of corrosive substances | 0 | 0 | excluded |
| T55 | Toxic effect of soaps and detergents | 0 | 0 | excluded |
| T56 | Toxic effect of metals | 0 | 0 | excluded |
| T57 | Toxic effect of other inorganic substances | 0 | 0 | excluded |
| T58 | Toxic effect of carbon monoxide | 0 | 0 | excluded |
| T59 | Toxic effect of other gases, fumes and vapours | 0 | 0 | excluded |
| T60 | Toxic effect of pesticides | 0 | 0 | excluded |
| T61 | Toxic effect of noxious substances eaten as seafood | 0 | 0 | excluded |
| T62 | Toxic effect of other noxious substances eaten as food | 0 | 0 | excluded |
| T63 | Toxic effect of contact with venomous animals | 0 | 0 | excluded |
| T64 | Toxic effect of aflatoxin and other mycotoxin food contaminants | 0 | 0 | excluded |
| T65 | Toxic effect of other and unspecified substances | 0 | 0 | excluded |
| T66 | Unspecified effects of radiation | 0 | 0 | excluded |
| T67 | Effects of heat and light | 0 | 0 | excluded |
| T68 | Hypothermia | 0 | 0 | excluded |
| T69 | Other effects of reduced temperature | 0 | 0 | excluded |
| T70 | Effects of air pressure and water pressure | 0 | 0 | excluded |
| T71 | Asphyxiation | 0 | 0 | excluded |
| T73 | Effects of other deprivation | 0 | 0 | excluded |
| T74 | Maltreatment syndromes | 0 | 0 | excluded |
| T75 | Effects of other external causes | 0 | 0 | excluded |
| T76 | Unspecified effects of external causes | 0 | 0 | excluded |
| T77 |  | 0 | 0 | excluded |
| T78 | Adverse effects, NEC | 0 | 0 | excluded |
| T79 | Certain early complications of trauma, NEC | 155 | 150 | 96.77 |
| T790 | Air embolism (traumatic) | 1 | 1 | 100.00 |
| T791 | Fat embolism (traumatic) | 0 | 0 | 0.00 |
| T792 | Traumatic secondary and recurrent hemorrhage | 1 | 1 | 100.00 |
| T793 | Post-traumatic wound infection, NEC | 28 | 28 | 100.00 |
| T794 | Traumatic shock | 6 | 1 | 16.67 |
| T795 | Traumatic anuria | 0 | 0 | 0.00 |
| T796 | Traumatic ischemia of muscle | 99 | 99 | 100.00 |
| T797 | Traumatic subcutaneous emphysema | 17 | 17 | 100.00 |
| T798 | Other early complications of trauma | 3 | 3 | 100.00 |
| T799 | Unspecified early complication of trauma | 0 | 0 | 0.00 |
| T80 | Complications following infusion, transfusion and therapeutic injection | 0 | 0 | excluded |
| T81 | Complications of procedures, NEC | 0 | 0 | excluded |
| T82 | Complications of cardiac and vascular prosthetic devices, implants and grafts | 0 | 0 | excluded |
| T83 | Complications of genitourinary prosthetic devices, implants and grafts | 0 | 0 | excluded |
| T84 | Complications of internal orthopedic prosthetic devices, implants and grafts | 0 | 0 | excluded |
| T85 | Complications of other internal prosthetic devices, implants and grafts | 0 | 0 | excluded |
| T86 | Failure and rejection of transplanted organs and tissues | 0 | 0 | excluded |
| T87 | Complications peculiar to reattachment and amputation | 0 | 0 | excluded |
| T88 | Other complications of surgical and medical care, NEC | 0 | 0 | excluded |
| T90 | Sequelae of injuries of head | 4 | 4 | 100.00 |
| T900 | Sequelae of superficial injury of head | 0 | 0 | 0.00 |
| T901 | Sequelae of open wound of head | 0 | 0 | 0.00 |
| T902 | Sequelae of fracture of skull and facial bones | 1 | 1 | 100.00 |
| T903 | Sequelae of injury of cranial nerves | 0 | 0 | 0.00 |
| T904 | Sequelae of injury of eye and orbit | 0 | 0 | 0.00 |
| T905 | Sequelae of intracranial injury | 1 | 1 | 100.00 |
| T908 | Sequelae of other specified injuries of head | 0 | 0 | 0.00 |
| T909 | Sequelae of unspecified injury of head | 2 | 2 | 100.00 |
| T91 | Sequelae of injuries of neck and trunk | 3 | 3 | 100.00 |
| T910 | Sequelae of superficial injury and open wound of neck and trunk | 0 | 0 | 0.00 |
| T911 | Sequelae of fracture of spine | 1 | 1 | 100.00 |
| T912 | Sequelae of other fracture of thorax and pelvis | 1 | 1 | 100.00 |
| T913 | Sequelae of injury of spinal cord | 1 | 1 | 100.00 |
| T914 | Sequelae of injury of intrathoracic organs | 0 | 0 | 0.00 |
| T915 | Sequelae of injury of intra-abdominal and pelvic organs | 0 | 0 | 0.00 |
| T918 | Sequelae of other specified injuries of neck and trunk | 0 | 0 | 0.00 |
| T919 | Sequelae of unspecified injury of neck and trunk | 0 | 0 | 0.00 |
| T92 | Sequelae of injuries of upper limb | 1 | 1 | 100.00 |
| T920 | Sequelae of open wound of upper limb | 0 | 0 | 0.00 |
| T921 | Sequelae of fracture of arm | 0 | 0 | 0.00 |
| T922 | Sequelae of fracture at wrist and hand level | 0 | 0 | 0.00 |
| T923 | Sequelae of dislocation, sprain and strain of upper limb | 0 | 0 | 0.00 |
| T924 | Sequelae of injury of nerve of upper limb | 0 | 0 | 0.00 |
| T925 | Sequelae of injury of muscle and tendon of upper limb | 0 | 0 | 0.00 |
| T926 | Sequelae of crushing injury and traumatic amputation of upper limb | 1 | 1 | 100.00 |
| T928 | Sequelae of other specified injuries of upper limb | 0 | 0 | 0.00 |
| T929 | Sequelae of unspecified injury of upper limb | 0 | 0 | 0.00 |
| T93 | Sequelae of injuries of lower limb | 3 | 3 | 100.00 |
| T930 | Sequelae of open wound of lower limb | 1 | 1 | 100.00 |
| T931 | Sequelae of fracture of femur | 0 | 0 | 0.00 |
| T932 | Sequelae of other fractures of lower limb | 1 | 1 | 100.00 |
| T933 | Sequelae of dislocation, sprain and strain of lower limb | 0 | 0 | 0.00 |
| T934 | Sequelae of injury of nerve of lower limb | 0 | 0 | 0.00 |
| T935 | Sequelae of injury of muscle and tendon of lower limb | 1 | 1 | 100.00 |
| T936 | Sequelae of crushing injury and traumatic amputation of lower limb | 0 | 0 | 0.00 |
| T938 | Sequelae of other specified injuries of lower limb | 0 | 0 | 0.00 |
| T939 | Sequelae of unspecified injury of lower limb | 0 | 0 | 0.00 |
| T94 | Sequelae of injuries involving multiple and unspecified body regions | 0 | 0 | 0.00 |
| T940 | Sequelae of injuries involving multiple body regions | 0 | 0 | 0.00 |
| T941 | Sequelae of injuries, not specified by body region | 0 | 0 | 0.00 |
| T95 | Sequelae of burns, corrosions and frostbite | 0 | 0 | 0.00 |
| T950 | Sequelae of burn, corrosion and frostbite of head and neck | 0 | 0 | 0.00 |
| T951 | Sequelae of burn, corrosion and frostbite of trunk | 0 | 0 | 0.00 |
| T952 | Sequelae of burn, corrosion and frostbite of upper limb | 0 | 0 | 0.00 |
| T953 | Sequelae of burn, corrosion and frostbite of lower limb | 0 | 0 | 0.00 |
| T954 | Sequelae of burn and corrosion classifiable only according to extent of body surface involved | 0 | 0 | 0.00 |
| T958 | Sequelae of other specified burn, corrosion and frostbite | 0 | 0 | 0.00 |
| T959 | Sequelae of unspecified burn, corrosion and frostbite | 0 | 0 | 0.00 |
| T96 | Sequelae of poisoning by drugs, medicaments and biological substances | 10 | 10 | 100.00 |
| T97 | Sequelae of toxic effects of substances chiefly nonmedicinal as to source | 1 | 1 | 100.00 |
| T98 | Sequelae of other and unspecified effects of external causes | 1 | 1 | 100.00 |
| T980 | Sequelae of effects of foreign body entering through natural orifice | 0 | 0 | 0.00 |
| T981 | Sequelae of other and unspecified effects of external causes | 0 | 0 | 0.00 |
| T982 | Sequelae of certain early complications of trauma | 0 | 0 | 0.00 |
| T983 | Sequelae of complications of surgical and medical care, NEC | 1 | 1 | 100.00 |
| ICD-10, international classification of diseases 10th version; ICISS, international classification of disease-based injury severity score, SRR, survival risk ratio | | | | |


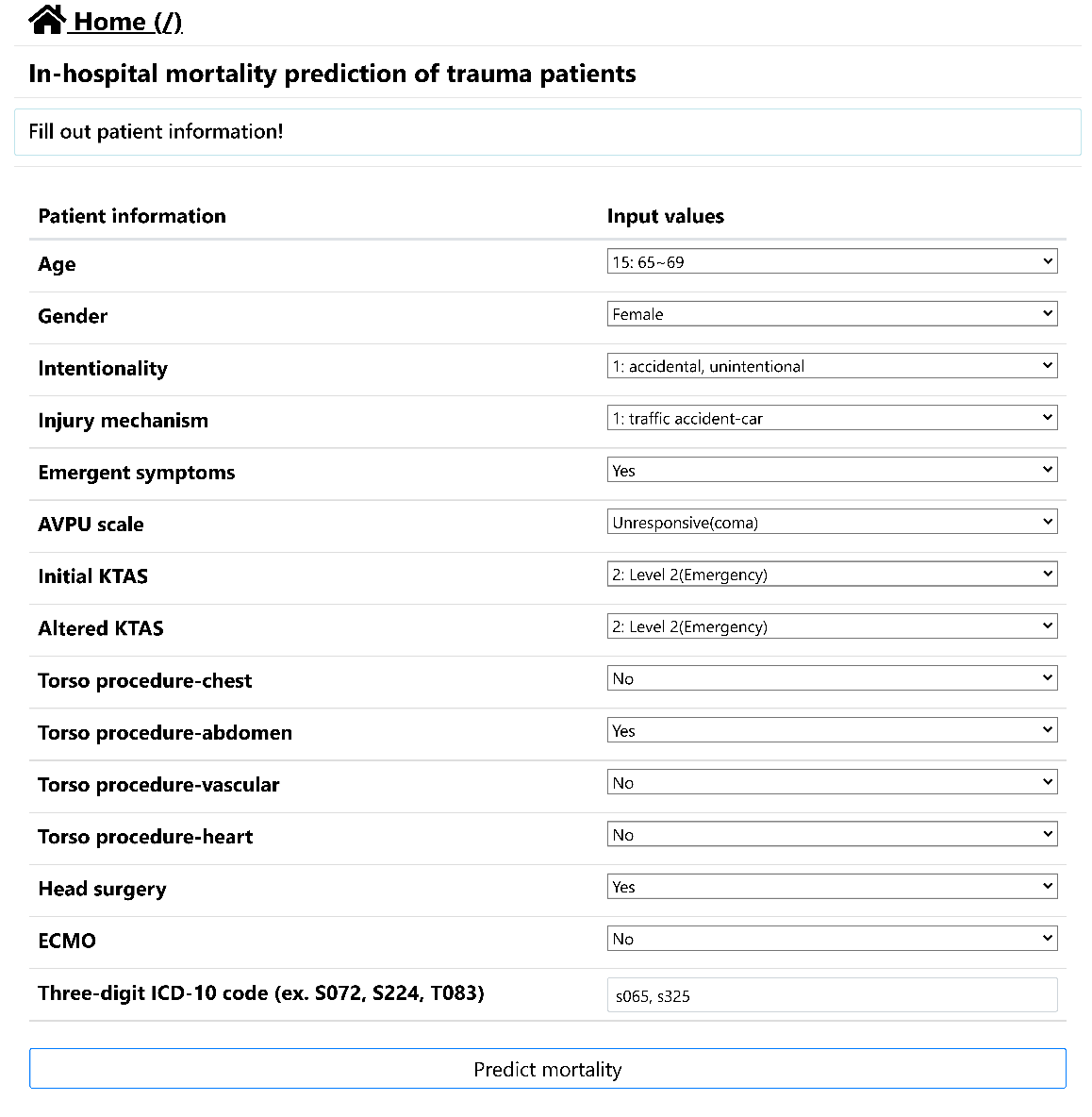


(a)

**
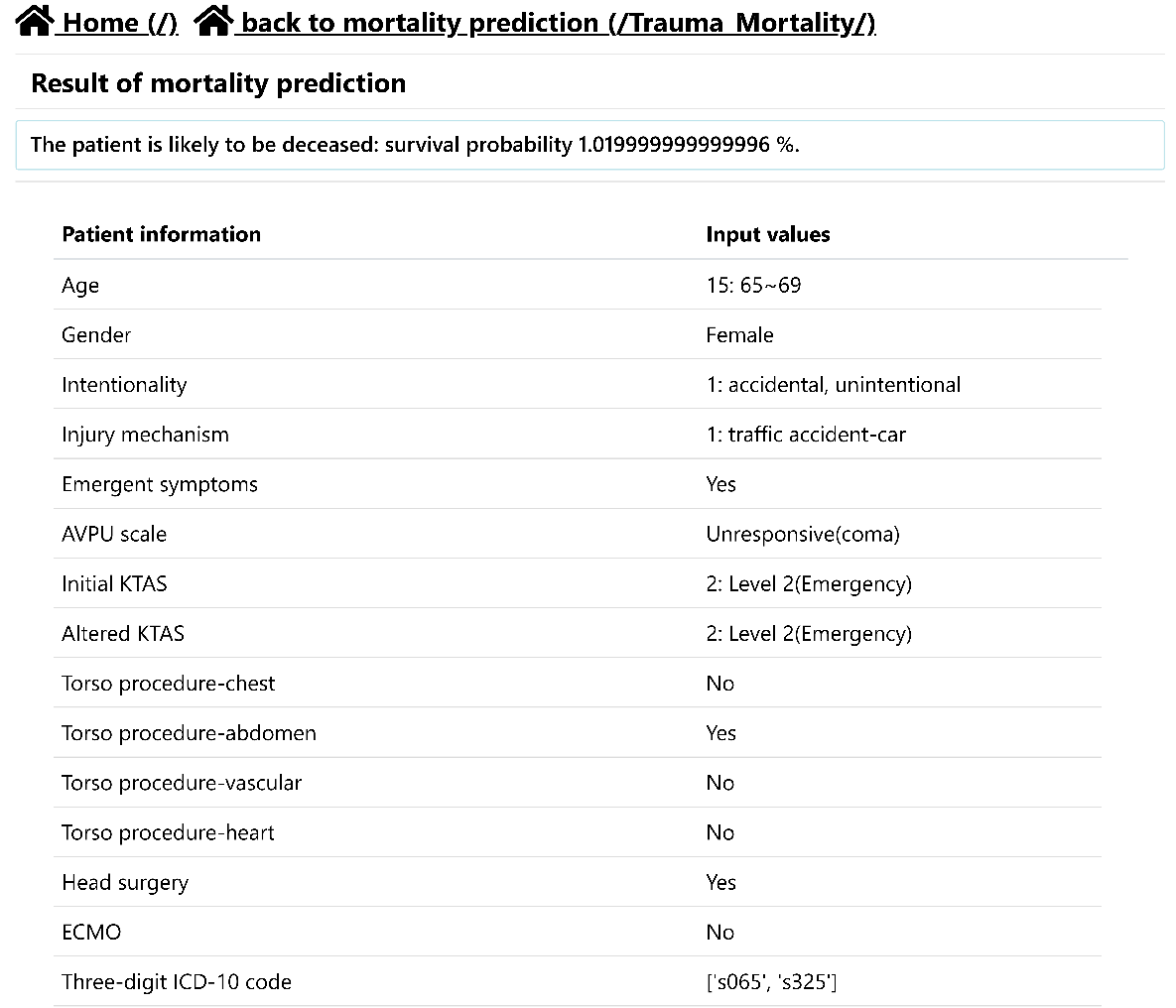
**
(b)

**Figure S1.** Deployed web application to provide mortality prediction in trauma patients: (a) a user's web interface to enter information, (b) the prediction results with the probability of mortality.
